# Supplementary material for: Adaptive RAG-Assisted MRI Platform (ARAMP) for Brain Metastasis Detection and Reporting: A Retrospective Evaluation Using Post-Contrast T1-Weighted Imaging
Source: Bioengineering (Basel). 2025 Jun 26;12(7):698. doi: 10.3390/bioengineering12070698 (PMC12292328; doi:10.3390/bioengineering12070698)
Supplement: Supplementary file 1 [file bioengineering-12-00698-s001.zip › bioengineering-3673052-supplementary.pdf]

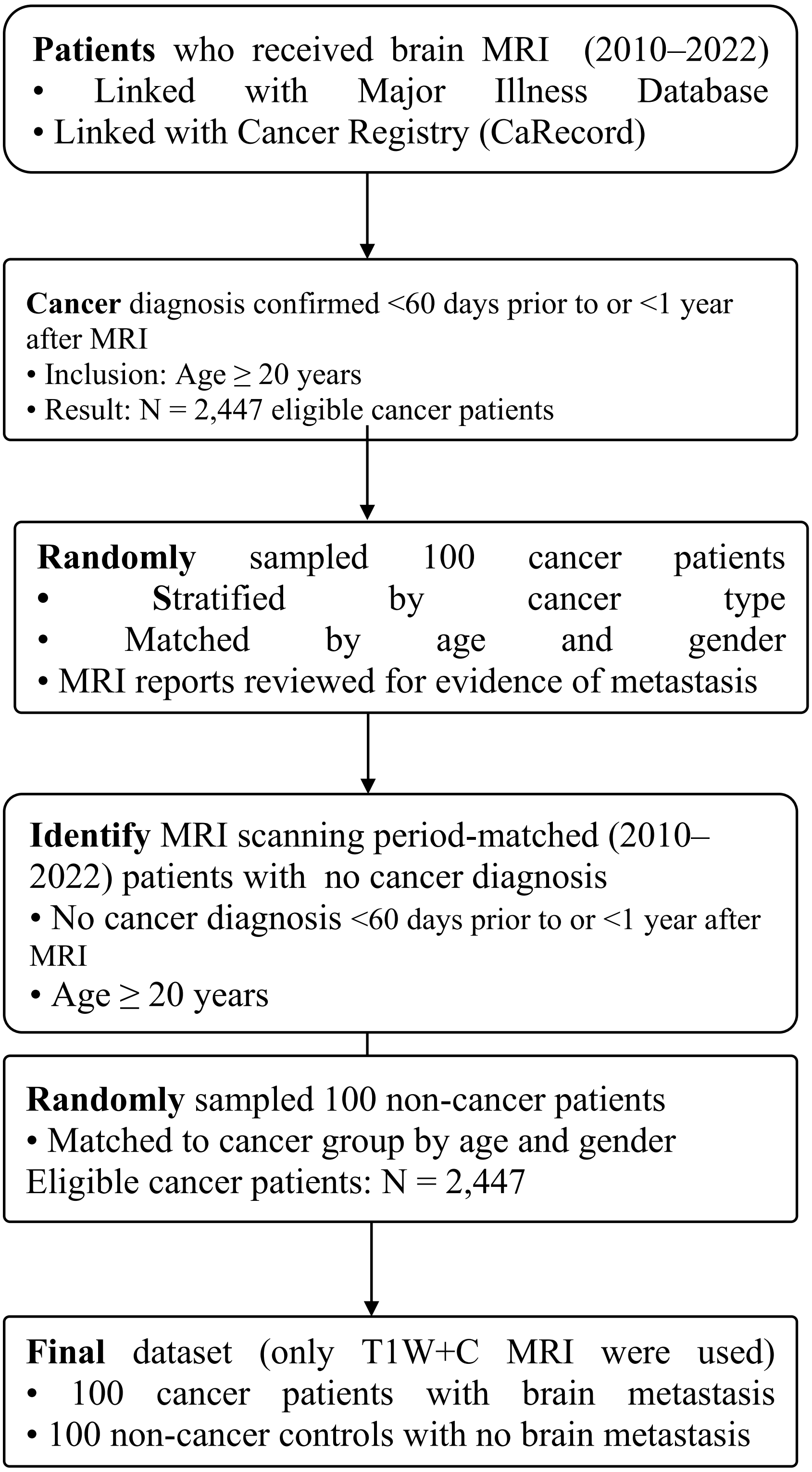

Figure S1. Flowchart of patient selection and group allocation. Patients who received AX_T1+C brain MRI between 2010 and 2022 were linked to major illness and cancer registry databases. Cancer and non-cancer cohorts were sampled according to diagnosis timing, age, sex, and cancer type distribution.

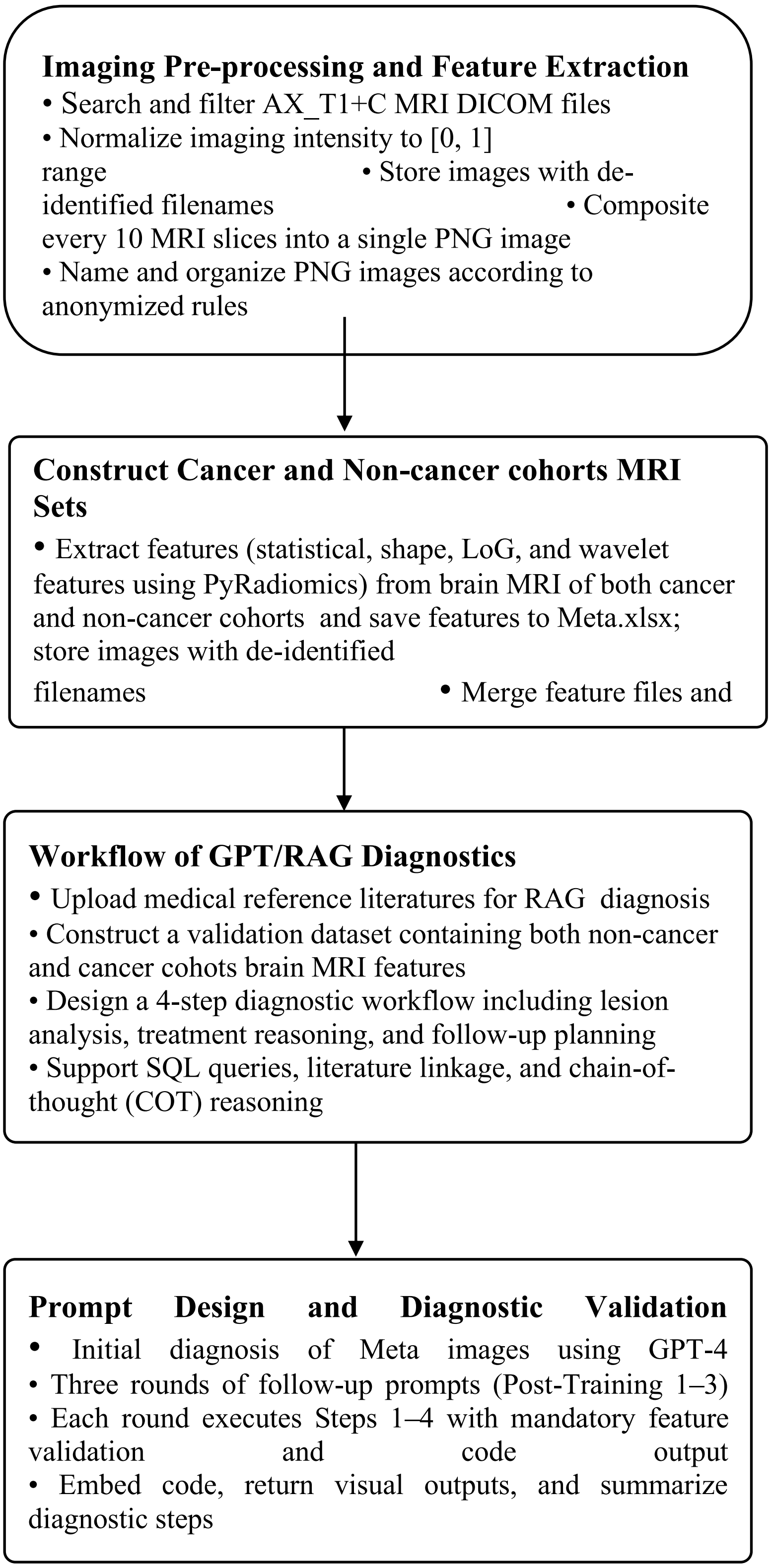

Figure S2. AI-Integrated Workflow for Feature Extraction and Multi-Stage Diagnostic Reasoning

Supplementary File S1. PyRadiomics YAML Configuration File

The following YAML-formatted configuration was used to initialize the PyRadiomics feature extractor. This file specifies image filtering types, LoG sigma values, voxel resampling, intensity normalization, and the selected feature classes, ensuring full reproducibility of the radiomic feature extraction process.


```yaml
imageType:
  Original: {}
  LoG:
    sigma: [3, 5]
  Wavelet: {}

setting:
  binWidth: 25
  resampledPixelSpacing: [1, 1, 1]
  interpolator: 'sitkBSpline'
  voxelArrayShift: 1000
  normalize: true
  normalizeScale: 100

featureClass:
  firstorder:
    - Energy
    - TotalEnergy
    - Entropy
    - Minimum
    - 10Percentile
    - 90Percentile
    - Maximum
    - Mean
    - Median
    - InterquartileRange
    - Range
    - MeanAbsoluteDeviation
    - RobustMeanAbsoluteDeviation
    - RootMeanSquared
    - StandardDeviation
    - Skewness
    - Kurtosis
    - Variance
    - Uniformity
  shape:
    - VoxelVolume
    - MeshVolume
    - SurfaceArea
    - SurfaceVolumeRatio
    - Compactness1
```

- Compactness2
- Sphericity
- SphericalDisproportion
- Maximum3DDiameter
- Maximum2DDiameterSlice
- Maximum2DDiameterColumn
- Maximum2DDiameterRow
- MajorAxisLength
- MinorAxisLength
- LeastAxisLength
- Elongation
- Flatness

Supplementary Table S1. Complete Radiomic Feature Summary

This table lists all 1,051 extracted radiomic features with their respective transformation category and the minimum–maximum value ranges observed in the No_abnormalities and Brain_Meta cohorts.

TYPE	**Category**	No_abnormalities (MIN-MAX)	Brain_Meta (MIN-MAX)
original_shape_VoxelVolume	original	1161288 - 6762600	2123604 - 12903200
original_shape_MeshVolume	original	1161025.66666667 - 6762077.66666667	2123276.16666667 - 12902846.6666667
original_shape_SurfaceArea	original	146702.999437058 - 572013.781541937	183236.82281355 – 569974.953114813
original_shape_SurfaceVolumeRatio	original	0.0489128412628021 - 0.140884142628377	0.0256843921140218 - 0.107470486869656
original_shape_Compactness1	original	0.00540187904409212 - 0.0265092401801502	0.00731725182074433 - 0.0381573634078484
original_shape_Compactness2	original	0.0103679276306154 - 0.249687502928745	0.0190238428155243 - 0.517319635206943
original_shape_Sphericity	original	0.218053963793387 - 0.629697934413258	0.266951736124722 - 0.802761102762934
original_shape_SphericalDisproportion	original	1.5880630145814 – 4.5860207381854	1.24570061573513 - 3.74599549160749
original_shape_Maximum3DDiameter	original	358.906673105976 - 720.975727746781	362.400331125676 - 720.941745219404
original_shape_Maximum2DDiameterSlice	original	358.503835404867 - 720.542156990138	358.503835404867 - 720.542156990138
original_shape_Maximum2DDiameterColumn	original	254.568261965234 - 510.612377444966	259.470614906583 - 510.564393588115
original_shape_Maximum2DDiameterRow	original	254.568261965234 - 510.612377444966	259.470614906583 - 510.564393588115
original_shape_MajorAxisLength	original	293.291663706967 - 588.896142513003	293.291663706967 - 588.896142512988
original_shape_MinorAxisLength	original	293.291663706965 - 588.896142512989	293.291663706963 - 588.896142512977

original_shape_LeastAxisLength	original	17.2819751957538 - 69.2724091299491	24.2212028327788 - 230.937220906448
original_shape_Elongation	original	0.899370018396879 - 0.999999999999999	0.999999999999787 – 1
original_shape_Flatness	original	0.0293463888590047 - 0.236189492242641	0.0411298378172765 - 0.787397834590802
original_firstorder_Energy	original	1176117623366.82 - 6847166104495.24	2140354281691.38 - 13069099825979.7
original_firstorder_TotalEnergy	original	1176117623366.82 - 6847166104495.24	2140354281691.38 - 13069099825979.7
original_firstorder_Entropy	original	2.3168493323015 – 3.76222820297295	2.22044465543238 - 3.44423324300398
original_firstorder_Minimum	original	-169.326047794837 - - 57.7847506893932	-130.632899690855 – -55.4767296774977
original_firstorder_10Percentile	original	-137.657730773214 - - 53.1636550189916	-94.0705809735378 - - 50.0079451619047
original_firstorder_90Percentile	original	108.173870326725 - 173.983836460042	120.641757168834 - 182.490129487186
original_firstorder_Maximum	original	243.894470735715 - 396.549989491342	230.522500027242 - 407.579774233323
original_firstorder_Mean	original	-1.54180700112038 - 3.89181089486698	-0.738447925135116 - 4.32884686812834
original_firstorder_Median	original	-70.3182932623198 - 3.19042129601344	-72.2795563388065 - - 41.8744556749553
original_firstorder_InterquartileRange	original	52.6804906425778 - 193.321494717336	18.4168431954736 - 189.114756735279
original_firstorder_Range	original	320.182079103346 - 529.829256667988	306.522311418432 - 503.269527925206
original_firstorder_MeanAbsoluteDeviation	original	75.4119566150081 – 91.610817315137	72.3096065989584 – 92.612811898892
original_firstorder_RobustMeanAbsoluteDeviation	original	42.3711126266489 - 76.9770863818464	35.9433239177474 - 79.6483852954253
original_firstorder_RootMeanSquared	original	1003.2890380602 – 1009.05020376864	1003.93608722628 – 1009.541690679
original_firstorder_StandardDeviation	original	96.9842180135167 – 102.38521979767	96.7678550974066 - 103.375567148343

original_firstorder_Skewness	original	0.579470430431558 - 1.95530703901419	0.616002353834428 - 2.24509964806163
original_firstorder_Kurtosis	original	1.91916272446236 - 6.68992138808033	1.85606351263105 - 7.59946776538822
original_firstorder_Variance	original	9405.93854369334 - 10482.7332330172	9364.01778015268 - 10686.5078832415
original_firstorder_Uniformity	original	0.0856259315308619 - 0.384213227934292	0.131287103092981 - 0.434105527659276
original_glcm_Autocorrelation	original	25.178737099023 – 68.767309998015	26.563190393441 – 57.2280544119626
original_glcm_ClusterProminence	original	6420.03641724627 - 22132.4026633211	6682.76398331638 - 28494.1642336637
original_glcm_ClusterShade	original	176.718710558175 - 932.618482121937	260.88117536164 – 1073.50535431757
original_glcm_ClusterTendency	original	51.8135573724205 - 66.5537020966514	53.7206727831808 - 66.3984390335829
original_glcm_Contrast	original	2.83409620037368 - 7.12176376148058	2.20968284480191 - 7.76252552651911
original_glcm_Correlation	original	0.765769522154579 - 0.911248487437586	0.757552787063043 - 0.935615469138221
original_glcm_DifferenceAverage	original	0.674135111364912 - 1.51095778820685	0.595975251010788 - 1.45563518277823
original_glcm_DifferenceEntropy	original	1.4493815795613 – 2.36358015214063	1.42800609499446 – 2.2937805302919
original_glcm_DifferenceVariance	original	2.11763025289398 - 5.42014677575948	1.84801471646912 - 5.47554614151508
original_glcm_Id	original	0.594471091461904 - 0.827916245915131	0.648885684069231 - 0.835752509355364
original_glcm_Idm	original	0.554154882806345 - 0.809141291040598	0.615616255938631 - 0.818171020388616
original_glcm_Idmn	original	0.980421919803377 - 0.991175467857148	0.984107555542966 - 0.993318167274016
original_glcm_Idn	original	0.937262381151769 - 0.967080448003279	0.943475974936277 - 0.970714252792876
original_glcm_Imc1	original	-0.569879423475276 - -0.277900478030492	-0.54299780356482 - -0.290722174225502

original_glcm_Imc2	original	0.911386791627443 - 0.98126646303422	0.909555049853078 - 0.977963196353014
original_glcm_InverseVariance	original	0.135608258924826 - 0.394333412600396	0.131661920966776 - 0.336159817287924
original_glcm_JointAverage	original	3.54804135878988 - 7.56703949139099	3.44258230898942 - 6.64890651964767
original_glcm_JointEnergy	original	0.0280522396334947 - 0.351488620919419	0.0567881016359699 - 0.374553293749802
original_glcm_JointEntropy	original	3.53167490671894 - 6.34425651918167	3.45982179845569 - 5.82621456571383
original_glcm_MCC	original	0.871725943035381 - 0.989246196711955	0.859994644492829 - 0.98706521692389
original_glcm_MaximumProbability	original	0.095389021497431 - 0.587322876295848	0.15081676215967 - 0.608149096359714
original_glcm_SumAverage	original	7.09608271757976 – 15.134078982782	6.88516461797884 - 13.2978130392953
original_glcm_SumEntropy	original	2.76538074749236 - 4.58473861889168	2.71336241258579 - 4.32929240843486
original_glcm_SumSquares	original	13.82455598365 – 17.7648814946175	14.5308891923128 - 17.4095052506009
original_gldm_DependenceEntropy	original	4.59809431615594 – 7.6307532644902	4.51534616133828 - 7.57975890812182
original_gldm_DependenceNonUniformity	original	83721.6757173183 - 1721519.34348512	84014.1197539654 – 2637712.7722055
original_gldm_DependenceNonUniformityNormalized	original	0.0394243351007619 - 0.283394905150392	0.0395620462920419 - 0.29194825392677
original_gldm_DependenceVariance	original	39.2694555099901 - 91.4813051243489	59.5134059879528 – 88.569485247858
original_gldm_GrayLevelNonUniformity	original	155213.583033666 - 2438883.98101838	278801.817276667 - 4933246.35024351
original_gldm_GrayLevelVariance	original	14.0937867122848 - 17.5272071492289	14.7162018297337 - 17.2407494815024
original_gldm_HighGrayLevelEmphasis	original	27.2587198903432 - 72.0408786705956	28.6917348298982 - 59.6011996586934
original_gldm_LargeDependenceEmphasis	original	130.207730424712 - 462.842448556575	207.989153344974 - 470.301945056097

original_gldm_LargeDependenceHighGrayLevelEmphasis	original	3280.13688433672 - 6896.81496171344	2437.43261826524 - 7318.71742109678
original_gldm_LargeDependenceLowGrayLevelEmphasis	original	16.3070797599441 - 403.293727497286	17.1486358503488 - 415.325613926553
original_gldm_LowGrayLevelEmphasis	original	0.0595098622117836 - 0.629901164224759	0.0599686415252961 - 0.641154751243459
original_gldm_SmallDependenceEmphasis	original	0.0187645237461625 - 0.077903653762099	0.0202163266012949 - 0.0678619704591989
original_gldm_SmallDependenceHighGrayLevelEmphasis	original	1.21346367433389 - 9.48671643009845	1.13921139698993 - 7.59030286697329
original_gldm_SmallDependenceLowGrayLevelEmphasis	original	0.00101706301488206 - 0.00429506805860035	0.00111529759780843 - 0.010633433897582
original_glrlm_GrayLevelNonUniformity	original	47029.1355815685 - 301758.730522871	92789.1377866244 - 437483.089616457
original_glrlm_GrayLevelNonUniformityNormalized	original	0.0770436966469349 - 0.171680513461111	0.0756079494535149 - 0.151220540382942
original_glrlm_GrayLevelVariance	original	11.5667157095456 - 22.0050322001643	11.6058561881576 - 26.7697051350045
original_glrlm_HighGrayLevelRunEmphasis	original	45.7591442987948 - 83.4359939637112	47.7354081383426 - 79.0905230061611
original_glrlm_LongRunEmphasis	original	4.93928043198576 – 274.9046695645	8.64425361794633 - 313.672814403825
original_glrlm_LongRunHighGrayLevelEmphasis	original	174.417499843359 - 1052.65647820613	146.836428217707 - 967.182634011254
original_glrlm_LongRunLowGrayLevelEmphasis	original	0.899276940710251 - 269.008029662195	0.790014683737756 - 306.278430814031
original_glrlm_LowGrayLevelRunEmphasis	original	0.0409628029071978 - 0.363562485055155	0.046010507848843 - 0.338307674137138
original_glrlm_RunEntropy	original	5.00835509952671 – 5.6722029404745	5.11302038465648 - 5.65360022554478
original_glrlm_RunLengthNonUniformity	original	275121.090164026 - 1596713.79539097	495823.046325769 - 1831672.18710227
original_glrlm_RunLengthNonUniformityNormalized	original	0.414414162848844 - 0.574637464040867	0.392079679692705 - 0.539350586581067
original_glrlm_RunPercentage	original	0.288988194661431 - 0.671686300314747	0.277155155494256 - 0.575160688368236

original_glrlm_RunVariance	original	2.70069443366887 - 257.809371215021	5.54470411480275 - 293.437126383641
original_glrlm_ShortRunEmphasis	original	0.662930998593872 - 0.785829961438685	0.640664813560521 - 0.761019816788225
original_glrlm_ShortRunHighGrayLevelEmphasis	original	37.5204262726867 - 71.0137056432641	39.7103356428312 - 66.8958488463173
original_glrlm_ShortRunLowGrayLevelEmphasis	original	0.0221104124191408 - 0.194208682554	0.0232233153062987 - 0.192924644000085
original_glszm_GrayLevelNonUniformity	original	2832.03604620446 - 13042.9317410637	5986.49368253189 - 27825.8217673736
original_glszm_GrayLevelNonUniformityNormalized	original	0.0584494936216833 - 0.0879234148540601	0.0566829749090315 - 0.0981295863598565
original_glszm_GrayLevelVariance	original	9.75475039837529 – 23.389248343067	8.84876621153373 - 32.1088163701282
original_glszm_HighGrayLevelZoneEmphasis	original	63.5806058332552 – 138.30315333604	55.8421704415838 - 124.056769667459
original_glszm_LargeAreaEmphasis	original	1745371.71711102 - 144196836.668222	4354282.75244206 - 231254831.706315
original_glszm_LargeAreaHighGrayLevelEmphasis	original	51943393.5127374 - 717414293.659461	54508326.2061977 - 886802859.636113
original_glszm_LargeAreaLowGrayLevelEmphasis	original	89928.2973139217 - 138284226.541693	269163.200395208 - 225634205.696344
original_glszm_LowGrayLevelZoneEmphasis	original	0.0203846877835554 - 0.146227864437982	0.0230487165988024 - 0.270709763041847
original_glszm_SizeZoneNonUniformity	original	12407.2732556352 - 47263.2662459454	15460.2604355827 - 104893.975208244
original_glszm_SizeZoneNonUniformityNormalized	original	0.178607141095837 - 0.383357600112121	0.188422571761255 - 0.371660445824501
original_glszm_SmallAreaEmphasis	original	0.418006536767976 - 0.642921368530151	0.432678603917433 - 0.633062356610768
original_glszm_SmallAreaHighGrayLevelEmphasis	original	30.7219495232056 - 87.6847943076844	24.5467900289677 - 76.4702909880633
original_glszm_SmallAreaLowGrayLevelEmphasis	original	0.0103423044688381 - 0.0867497971588889	0.0109677969767382 - 0.171503442636086
original_glszm_ZoneEntropy	original	6.274561364753 – 7.53498814951218	6.18403335663411 – 7.337778173188

original_glszm_ZonePercentage	original	0.0113659082171193 - 0.0677035228922021	0.013715627768584 - 0.0612162154525985
original_glszm_ZoneVariance	original	1745153.55593542 - 144191339.072628	4353939.10036743 - 231252546.656006
original_ngtdm_Busyness	original	492.426952840576 - 11480.6330618919	1553.50352657582 - 16552.2384761472
original_ngtdm_Coarseness	original	2.00744050289882E-06 - 2.13830393922749E-05	1.69052983706658E-06 - 5.73576527112685E-06
original_ngtdm_Complexity	original	34.9217740169191 - 221.373711228602	30.3524991625704 - 204.270806629922
original_ngtdm_Contrast	original	0.0517239538839046 - 0.129765283187867	0.0427359123397895 - 0.114716739426489
original_ngtdm_Strength	original	0.000283491923175294 - 0.00334963276561785	0.000302080833702337 - 0.00174771559671076
log-sigma-3-mm-3D_firstorder_Energy	log-sigma-3-mm-3D	1161696272135.58 – 6761899593877.8	2116351985741.41 – 12909773448975
log-sigma-3-mm-3D_firstorder_TotalEnergy	log-sigma-3-mm-3D	1161696272135.58 – 6761899593877.8	2116351985741.41 – 12909773448975
log-sigma-3-mm-3D_firstorder_Entropy	log-sigma-3-mm-3D	1.88240125285725 - 2.27291775144175	1.77048040952427 - 2.26366153207573
log-sigma-3-mm-3D_firstorder_Minimum	log-sigma-3-mm-3D	-230.942001342773 - -137.29963684082	-255.606506347656 - -132.486923217773
log-sigma-3-mm-3D_firstorder_10Percentile	log-sigma-3-mm-3D	-37.7595378875732 - -20.8831111907959	-40.325297164917 - -17.1429996490478
log-sigma-3-mm-3D_firstorder_90Percentile	log-sigma-3-mm-3D	20.0646762847901 - 35.3801654815674	18.2221569061279 - 37.3962673187256
log-sigma-3-mm-3D_firstorder_Maximum	log-sigma-3-mm-3D	103.418151855469 - 173.427291870117	94.1689071655273 - 214.140472412109
log-sigma-3-mm-3D_firstorder_Mean	log-sigma-3-mm-3D	-3.03763371962302 - 1.94520058628309	-3.91680560533075 – 1.807014871686
log-sigma-3-mm-3D_firstorder_Median	log-sigma-3-mm-3D	-0.446117490530014 - 0.302918910980225	-0.321319699287415 - 0.13147160410881
log-sigma-3-mm-3D_firstorder_InterquartileRange	log-sigma-3-mm-3D	2.28624226152896 - 26.8164191246032	1.43717894703149 - 22.7605922222137
log-sigma-3-mm-3D_firstorder_Range	log-sigma-3-mm-3D	264.903663635253 - 383.567169189453	256.005760192871 - 459.715667724609

log-sigma-3-mm-3D_firstorder_MeanAbsoluteDeviation	log-sigma-3-mm-3D	12.2043721411485 - 20.2778724516411	11.4298277764797 – 19.141804697289
log-sigma-3-mm-3D_firstorder_RobustMeanAbsoluteDeviation	log-sigma-3-mm-3D	4.0288238203483 – 11.7335939725901	2.83277509375286 - 9.96709686058791
log-sigma-3-mm-3D_firstorder_RootMeanSquared	log-sigma-3-mm-3D	997.259131947918 - 1002.41782422558	996.353094076377 - 1002.26593875284
log-sigma-3-mm-3D_firstorder_StandardDeviation	log-sigma-3-mm-3D	21.8367029875749 - 30.9462307016048	19.2313116829397 - 32.0390502972523
log-sigma-3-mm-3D_firstorder_Skewness	log-sigma-3-mm-3D	-1.49664057866711 - 0.021311586238425	-1.9181610183579 - 0.0252450662641198
log-sigma-3-mm-3D_firstorder_Kurtosis	log-sigma-3-mm-3D	4.73048719555723 - 12.1671970335082	5.50335909669989 – 12.204907994136
log-sigma-3-mm-3D_firstorder_Variance	log-sigma-3-mm-3D	476.841597367564 - 957.669194636946	369.843349046374 - 1026.50074394986
log-sigma-3-mm-3D_firstorder_Uniformity	log-sigma-3-mm-3D	0.262669231609756 - 0.344785306045775	0.270331032099434 - 0.369073447184101
log-sigma-3-mm-3D_glcm_Autocorrelation	log-sigma-3-mm-3D	43.0342149952306 - 110.850849667449	42.739858707723 – 133.607830670553
log-sigma-3-mm-3D_glcm_ClusterProminence	log-sigma-3-mm-3D	74.3610717555178 - 337.993152269321	46.1594402813084 - 358.051782913155
log-sigma-3-mm-3D_glcm_ClusterShade	log-sigma-3-mm-3D	-17.6971394483235 - - 0.869872072321687	-23.4207022569577 - - 0.758693450339882
log-sigma-3-mm-3D_glcm_ClusterTendency	log-sigma-3-mm-3D	3.48751023110351 - 6.62954312690451	2.86098613934065 - 7.03318889323535
log-sigma-3-mm-3D_glcm_Contrast	log-sigma-3-mm-3D	0.230748740277906 - 0.36950536146371	0.21031358431259 - 0.364037176020786
log-sigma-3-mm-3D_glcm_Correlation	log-sigma-3-mm-3D	0.868272746470801 - 0.905026589551214	0.863215223770127 - 0.916002813443455
log-sigma-3-mm-3D_glcm_DifferenceAverage	log-sigma-3-mm-3D	0.210488413378712 - 0.306650256137836	0.198922901042149 - 0.303713895371186
log-sigma-3-mm-3D_glcm_DifferenceEntropy	log-sigma-3-mm-3D	0.758714343829965 - 0.940595319123855	0.719231708599434 - 0.949713826613573
log-sigma-3-mm-3D_glcm_DifferenceVariance	log-sigma-3-mm-3D	0.181677742464717 - 0.262557113491251	0.16543679897911 - 0.285294172031458
log-sigma-3-mm-3D_glcm_Id	log-sigma-3-mm-3D	0.853596064425176 - 0.897988861835684	0.856804668412388 - 0.902415824279171

log-sigma-3-mm-3D_glcm_Idm	log-sigma-3-mm-3D	0.850953031581641 - 0.896777510396694	0.853645161734659 - 0.901677581688329
log-sigma-3-mm-3D_glcm_Idmn	log-sigma-3-mm-3D	0.998008086873814 - 0.998929059971845	0.99787437975125 - 0.99913635528283
log-sigma-3-mm-3D_glcm_Idn	log-sigma-3-mm-3D	0.979692767489935 - 0.986456087608116	0.980074723312774 - 0.988006545212541
log-sigma-3-mm-3D_glcm_Imc1	log-sigma-3-mm-3D	-0.549485494151592 – -0.474383502124534	-0.589595789604021 - - 0.492024618563281
log-sigma-3-mm-3D_glcm_Imc2	log-sigma-3-mm-3D	0.921705428509706 - 0.946663428346149	0.911012825326934 - 0.951669029874463
log-sigma-3-mm-3D_glcm_InverseVariance	log-sigma-3-mm-3D	0.192059064494362 - 0.271190896022145	0.177235743922527 - 0.260372771932352
log-sigma-3-mm-3D_glcm_JointAverage	log-sigma-3-mm-3D	6.49023159988528 - 10.4598918591812	6.4738274003193 – 11.4999194485643
log-sigma-3-mm-3D_glcm_JointEnergy	log-sigma-3-mm-3D	0.158975609785775 - 0.250424043037665	0.16794871292467 - 0.269224755420071
log-sigma-3-mm-3D_glcm_JointEntropy	log-sigma-3-mm-3D	2.77076355949999 - 3.44276214844563	2.6128754208248 – 3.43177461463077
log-sigma-3-mm-3D_glcm_MCC	log-sigma-3-mm-3D	0.873169000070514 - 0.914032773607758	0.869838175444907 - 0.923324253538623
log-sigma-3-mm-3D_glcm_MaximumProbability	log-sigma-3-mm-3D	0.275268975824794 - 0.428594064095404	0.28547562812035 - 0.429257908392084
log-sigma-3-mm-3D_glcm_SumAverage	log-sigma-3-mm-3D	12.9804631997706 - 20.9197837183623	12.9476548006386 - 22.9998388971286
log-sigma-3-mm-3D_glcm_SumEntropy	log-sigma-3-mm-3D	2.50830736940553 - 3.03680396931808	2.37731350517857 - 3.01440890386304
log-sigma-3-mm-3D_glcm_SumSquares	log-sigma-3-mm-3D	0.929564742845354 - 1.74013167405063	0.76782493091331 - 1.84930651731403
log-sigma-3-mm-3D_gldm_DependenceEntropy	log-sigma-3-mm-3D	5.18053639974678 - 6.19687403103917	5.09328661919387 - 6.04659165237473
log-sigma-3-mm-3D_gldm_DependenceNonUniformity	log-sigma-3-mm-3D	153718.912273436 – 1130555.9165132	233911.095569607 - 2486947.99319425
log-sigma-3-mm-3D_gldm_DependenceNonUniformityNormalized	log-sigma-3-mm-3D	0.0928504080684099 - 0.197319981946295	0.110148170548561 - 0.227311017212378
log-sigma-3-mm-3D_gldm_DependenceVariance	log-sigma-3-mm-3D	36.3392738959223 - 45.5801629193494	35.1669744719854 - 47.0131578774156

log-sigma-3-mm-3D_gldm_GrayLevelNonUniformity	log-sigma-3-mm-3D	383370.696111559 - 2157420.32688258	574076.061090486 - 4486097.93859988
log-sigma-3-mm-3D_gldm_GrayLevelVariance	log-sigma-3-mm-3D	0.923846584935068 - 1.72104380970704	0.76258392496189 - 1.8310829576903
log-sigma-3-mm-3D_gldm_HighGrayLevelEmphasis	log-sigma-3-mm-3D	43.2941534192535 - 110.887948549216	42.9833164501947 - 133.808596760051
log-sigma-3-mm-3D_gldm_LargeDependenceEmphasis	log-sigma-3-mm-3D	396.466990046563 - 484.112523068051	409.422400786587 - 508.614810434621
log-sigma-3-mm-3D_gldm_LargeDependenceHighGrayLevelEmphasis	log-sigma-3-mm-3D	18578.7504872485 - 52258.6294966971	19726.799894355 - 65317.9523135396
log-sigma-3-mm-3D_gldm_LargeDependenceLowGrayLevelEmphasis	log-sigma-3-mm-3D	4.07540420950426 - 11.7340259053453	3.73932800282215 - 11.9378757324292
log-sigma-3-mm-3D_gldm_LowGrayLevelEmphasis	log-sigma-3-mm-3D	0.00970018658124245 - 0.0263028869698249	0.00795238309794754 - 0.0261780425703804
log-sigma-3-mm-3D_gldm_SmallDependenceEmphasis	log-sigma-3-mm-3D	0.0035185518589871 - 0.00499219376668349	0.00335678203060531 - 0.00504337601419323
log-sigma-3-mm-3D_gldm_SmallDependenceHighGrayLevelEmphasis	log-sigma-3-mm-3D	0.16235330400807 - 0.52586571543252	0.145894524449814 - 0.540804161778335
log-sigma-3-mm-3D_gldm_SmallDependenceLowGrayLevelEmphasis	log-sigma-3-mm-3D	4.47009647984119E-05 - 0.000117697001609816	3.84481271192824E-05 - 0.00010680298158406
log-sigma-3-mm-3D_glrlm_GrayLevelNonUniformity	log-sigma-3-mm-3D	68599.0117936503 - 412643.087869904	119141.282532557 - 549944.907565672
log-sigma-3-mm-3D_glrlm_GrayLevelNonUniformityNormalized	log-sigma-3-mm-3D	0.186379379265048 - 0.262576860199346	0.181048310415751 - 0.280460669043413
log-sigma-3-mm-3D_glrlm_GrayLevelVariance	log-sigma-3-mm-3D	1.50973232761085 - 3.32134503093002	1.23114442752569 - 3.57209547055024
log-sigma-3-mm-3D_glrlm_HighGrayLevelRunEmphasis	log-sigma-3-mm-3D	43.5040465110582 – 109.13027920348	42.4852613552428 - 132.208276116771
log-sigma-3-mm-3D_glrlm_LongRunEmphasis	log-sigma-3-mm-3D	47.5898586648907 - 243.360399072059	68.0325851559946 - 354.159980846345
log-sigma-3-mm-3D_glrlm_LongRunHighGrayLevelEmphasis	log-sigma-3-mm-3D	3474.06119202983 - 22757.7583665419	5120.00890197517 - 40192.6665364764
log-sigma-3-mm-3D_glrlm_LongRunLowGrayLevelEmphasis	log-sigma-3-mm-3D	0.677178291363349 - 3.02733662196035	0.559144554260994 - 3.87246938270954
log-sigma-3-mm-3D_glrlm_LowGrayLevelRunEmphasis	log-sigma-3-mm-3D	0.0102427693523947 - 0.0280904013156971	0.00863419965448442 - 0.027939216089425

log-sigma-3-mm-3D_glrlm_RunEntropy	log-sigma-3-mm-3D	5.2641321260902 – 5.83170626890545	5.41174086803322 - 6.01065408505574
log-sigma-3-mm-3D_glrlm_RunLengthNonUniformity	log-sigma-3-mm-3D	49100.7017427471 – 486749.36389453	111474.094379117 - 426738.499402943
log-sigma-3-mm-3D_glrlm_RunLengthNonUniformityNormalized	log-sigma-3-mm-3D	0.152779881785325 - 0.220566805029063	0.147968856906922 - 0.230937150278979
log-sigma-3-mm-3D_glrlm_RunPercentage	log-sigma-3-mm-3D	0.224886656019874 - 0.313259446563945	0.20362879571913 - 0.303310895425527
log-sigma-3-mm-3D_glrlm_RunVariance	log-sigma-3-mm-3D	32.7545219336994 – 186.30514564955	47.479750840105 – 284.024128021289
log-sigma-3-mm-3D_glrlm_ShortRunEmphasis	log-sigma-3-mm-3D	0.34303513553369 - 0.443302197970915	0.327946216349936 - 0.473023085381903
log-sigma-3-mm-3D_glrlm_ShortRunHighGrayLevelEmphasis	log-sigma-3-mm-3D	15.5846977882979 - 45.7641450511676	13.9180355709005 - 50.6066741160129
log-sigma-3-mm-3D_glrlm_ShortRunLowGrayLevelEmphasis	log-sigma-3-mm-3D	0.0044764865882642 - 0.0112224217886012	0.00376035497774957 - 0.0105991301053096
log-sigma-3-mm-3D_glszm_GrayLevelNonUniformity	log-sigma-3-mm-3D	42.031746031746 – 210.136535348084	50.3572778827977 - 181.056801195815
log-sigma-3-mm-3D_glszm_GrayLevelNonUniformityNormalized	log-sigma-3-mm-3D	0.0886405983127167 - 0.143973948354257	0.0786301642692634 - 0.139442877181007
log-sigma-3-mm-3D_glszm_GrayLevelVariance	log-sigma-3-mm-3D	5.28694576537016 - 12.5540036262439	4.48420358342215 – 15.109457633309
log-sigma-3-mm-3D_glszm_HighGrayLevelZoneEmphasis	log-sigma-3-mm-3D	43.7287958115183 - 110.063985374772	44.8152466367713 - 129.009461426492
log-sigma-3-mm-3D_glszm_LargeAreaEmphasis	log-sigma-3-mm-3D	695020034.592593 - 11082167222.6113	1421326654.32609 - 27967787002.2639
log-sigma-3-mm-3D_glszm_LargeAreaHighGrayLevelEmphasis	log-sigma-3-mm-3D	41514435808.3571 - 1065064557023.42	105025459994.837 - 3275706029649.73
log-sigma-3-mm-3D_glszm_LargeAreaLowGrayLevelEmphasis	log-sigma-3-mm-3D	11864694.6864487 - 193113012.052418	17088590.0312065 - 296825828.225714
log-sigma-3-mm-3D_glszm_LowGrayLevelZoneEmphasis	log-sigma-3-mm-3D	0.0159913960474181 - 0.0530408534480639	0.0153319310781007 - 0.0509972983544031
log-sigma-3-mm-3D_glszm_SizeZoneNonUniformity	log-sigma-3-mm-3D	5.76923076923077 – 26.148407987048	6.85915492957746 – 25.080058224163
log-sigma-3-mm-3D_glszm_SizeZoneNonUniformityNormalized	log-sigma-3-mm-3D	0.0119041457481497 - 0.0180588708407995	0.0113164872942486 - 0.0200527089533508

log-sigma-3-mm-3D_glszm_SmallAreaEmphasis	log-sigma-3-mm-3D	0.0521397659058202 - 0.0899837343785796	0.0507141911659792 - 0.103675664510699
log-sigma-3-mm-3D_glszm_SmallAreaHighGrayLevelEmphasis	log-sigma-3-mm-3D	2.15717153486285 - 8.12817767133037	2.84586752869181 - 11.8951929964775
log-sigma-3-mm-3D_glszm_SmallAreaLowGrayLevelEmphasis	log-sigma-3-mm-3D	0.000996884207203512 - 0.00776617628739194	0.000865187349671467 - 0.00664362770027423
log-sigma-3-mm-3D_glszm_ZoneEntropy	log-sigma-3-mm-3D	8.16131947442829 - 9.54787714517701	8.38654068176147 - 9.72023027694722
log-sigma-3-mm-3D_glszm_ZonePercentage	log-sigma-3-mm-3D	0.000121806298276886 - 0.000415988623535793	0.000116738317430359 - 0.000327744720767149
log-sigma-3-mm-3D_glszm_ZoneVariance	log-sigma-3-mm-3D	685581680.270597 - 11049520242.6193	1410453004.70605 - 27912984538.4985
log-sigma-3-mm-3D_ngtdm_Busyness	log-sigma-3-mm-3D	354.625329422842 - 2334.60580472317	306.209856352929 - 2567.40458949677
log-sigma-3-mm-3D_ngtdm_Coarseness	log-sigma-3-mm-3D	3.27502543152557E-06 - 2.05551425990443E-05	1.93289335101652E-06 - 1.15009112340175E-05
log-sigma-3-mm-3D_ngtdm_Complexity	log-sigma-3-mm-3D	10.5252985174388 - 23.4758063474486	8.85113605260062 - 28.9346470207718
log-sigma-3-mm-3D_ngtdm_Contrast	log-sigma-3-mm-3D	0.00155989788559839 - 0.00355336121788739	0.00134263817159178 - 0.00310733498394115
log-sigma-3-mm-3D_ngtdm_Strength	log-sigma-3-mm-3D	0.000305641960749374 - 0.00223530645270924	0.000252821727984903 - 0.00248355321413662
log-sigma-5-mm-3D_firstorder_Energy	log-sigma-5-mm-3D	1161086066757.75 - 6748043967230.37	2104882295476.22 - 12906184283893.4
log-sigma-5-mm-3D_firstorder_TotalEnergy	log-sigma-5-mm-3D	1161086066757.75 - 6748043967230.37	2104882295476.22 - 12906184283893.4
log-sigma-5-mm-3D_firstorder_Entropy	log-sigma-5-mm-3D	1.81947174950285 - 2.21580556965643	1.71772123258342 - 2.35933924172747
log-sigma-5-mm-3D_firstorder_Minimum	log-sigma-5-mm-3D	-236.414916992187 - -140.158248901367	-241.190490722656 - -119.530075073242
log-sigma-5-mm-3D_firstorder_10Percentile	log-sigma-5-mm-3D	-47.4072971343994 - -14.425518321991	-66.0967704772949 - -23.1075031280518
log-sigma-5-mm-3D_firstorder_90Percentile	log-sigma-5-mm-3D	14.6293376922608 - 37.620333480835	13.8824248313904 - 36.9085010528564
log-sigma-5-mm-3D_firstorder_Maximum	log-sigma-5-mm-3D	82.1269378662109 - 137.54426574707	72.4675369262695 - 233.271759033203

log-sigma-5-mm-3D_firstorder_Mean	log-sigma-5-mm-3D	-7.6764709605625 - 4.85223864257491	-8.66147009266823 - 3.47757052648804
log-sigma-5-mm-3D_firstorder_Median	log-sigma-5-mm-3D	-1.527099609375 - 2.32304835319519	-1.28739672899246 - 0.248809784650803
log-sigma-5-mm-3D_firstorder_InterquartileRange	log-sigma-5-mm-3D	4.41719907522201 - 27.7429683208466	3.42735457420349 - 26.1732156276703
log-sigma-5-mm-3D_firstorder_Range	log-sigma-5-mm-3D	232.849746704102 - 368.726470947265	220.957221984863 – 466.013671875
log-sigma-5-mm-3D_firstorder_MeanAbsoluteDeviation	log-sigma-5-mm-3D	12.8088012477999 - 19.8948213876367	12.6437736116182 - 25.6848324879216
log-sigma-5-mm-3D_firstorder_RobustMeanAbsoluteDeviation	log-sigma-5-mm-3D	4.97976994170582 - 11.7619955201507	4.49730133926584 - 13.0747619146709
log-sigma-5-mm-3D_firstorder_RootMeanSquared	log-sigma-5-mm-3D	992.666932824633 - 1005.24041453755	991.676447863841 - 1003.88756576837
log-sigma-5-mm-3D_firstorder_StandardDeviation	log-sigma-5-mm-3D	19.8591407594485 - 31.1311923637823	18.9036057295933 - 36.2890463637533
log-sigma-5-mm-3D_firstorder_Skewness	log-sigma-5-mm-3D	-2.59208233779987 - 0.0602057505246494	-2.43015412880714 - - 0.443543242054519
log-sigma-5-mm-3D_firstorder_Kurtosis	log-sigma-5-mm-3D	4.53375725871315 - 15.0091307627225	4.25688815761168 - 15.1045371610233
log-sigma-5-mm-3D_firstorder_Variance	log-sigma-5-mm-3D	394.385471703589 - 969.151137990815	357.346309579912 - 1316.89488599063
log-sigma-5-mm-3D_firstorder_Uniformity	log-sigma-5-mm-3D	0.276574486925337 - 0.359857168012945	0.258807908249392 - 0.383411800643546
log-sigma-5-mm-3D_glcm_Autocorrelation	log-sigma-5-mm-3D	39.5002136126866 - 114.528025702176	29.5417467452847 - 111.533799931164
log-sigma-5-mm-3D_glcm_ClusterProminence	log-sigma-5-mm-3D	46.7303512934849 - 383.599055914929	38.8457348315459 - 417.647060001307
log-sigma-5-mm-3D_glcm_ClusterShade	log-sigma-5-mm-3D	-29.1064344623706 - - 0.921352492579478	-35.8034706269342 - - 3.05141485336471
log-sigma-5-mm-3D_glcm_ClusterTendency	log-sigma-5-mm-3D	3.0705402223719 - 6.74926215312829	2.93438123941447 - 8.94874887802496
log-sigma-5-mm-3D_glcm_Contrast	log-sigma-5-mm-3D	0.135258325873285 - 0.189992801058024	0.119973363444767 - 0.187404328897115
log-sigma-5-mm-3D_glcm_Correlation	log-sigma-5-mm-3D	0.913551032211848 - 0.948022867572592	0.914069890890908 - 0.95918191616634

log-sigma-5-mm-3D_glcm_DifferenceAverage	log-sigma-5-mm-3D	0.133179141219459 - 0.186918362750689	0.119815169467101 - 0.17791219647426
log-sigma-5-mm-3D_glcm_DifferenceEntropy	log-sigma-5-mm-3D	0.557264120616827 - 0.689886074880473	0.514228606327115 - 0.682406289589871
log-sigma-5-mm-3D_glcm_DifferenceVariance	log-sigma-5-mm-3D	0.11542423383021 - 0.152617881999838	0.103665565197517 - 0.155970109315165
log-sigma-5-mm-3D_glcm_Id	log-sigma-5-mm-3D	0.907052823310908 - 0.933756825850637	0.912030304109805 - 0.940118780929394
log-sigma-5-mm-3D_glcm_Idm	log-sigma-5-mm-3D	0.906848262455389 - 0.933618347855653	0.91184572988744 - 0.940108234664216
log-sigma-5-mm-3D_glcm_Idmn	log-sigma-5-mm-3D	0.998334026065322 - 0.999367147619525	0.998353007904837 - 0.999534007732857
log-sigma-5-mm-3D_glcm_Idn	log-sigma-5-mm-3D	0.984845430662129 - 0.990853140673545	0.985011470262742 - 0.991814713366991
log-sigma-5-mm-3D_glcm_Imc1	log-sigma-5-mm-3D	-0.661294993777054 - - 0.619104538093417	-0.686776899203647 - - 0.616554678880026
log-sigma-5-mm-3D_glcm_Imc2	log-sigma-5-mm-3D	0.946869527557877 - 0.971045449106011	0.943463566150931 - 0.977606729737797
log-sigma-5-mm-3D_glcm_InverseVariance	log-sigma-5-mm-3D	0.131361123180478 - 0.184232023049083	0.119676749736643 - 0.174439166195116
log-sigma-5-mm-3D_glcm_JointAverage	log-sigma-5-mm-3D	6.21117656157521 - 10.6407360133945	5.35197786111675 - 10.4688346791769
log-sigma-5-mm-3D_glcm_JointEnergy	log-sigma-5-mm-3D	0.202954164617952 - 0.293222733175012	0.203116302706899 - 0.317749068370005
log-sigma-5-mm-3D_glcm_JointEntropy	log-sigma-5-mm-3D	2.48675981598275 - 3.0328955163978	2.30446240353565 - 3.11615148835337
log-sigma-5-mm-3D_glcm_MCC	log-sigma-5-mm-3D	0.918731340416257 - 0.959561245265196	0.91771588918298 - 0.961446814380827
log-sigma-5-mm-3D_glcm_MaximumProbability	log-sigma-5-mm-3D	0.322361188612388 - 0.466141543433501	0.324094881441529 - 0.493436213232694
log-sigma-5-mm-3D_glcm_SumAverage	log-sigma-5-mm-3D	12.4223531231504 - 21.2814720267889	10.7039557222335 - 20.9376693583539
log-sigma-5-mm-3D_glcm_SumEntropy	log-sigma-5-mm-3D	2.3465110195272 - 2.83362706669943	2.18373484506051 - 2.91230291547195
log-sigma-5-mm-3D_glcm_SumSquares	log-sigma-5-mm-3D	0.802348298719461 - 1.73238522792347	0.76358865071481 - 2.28403830173052

log-sigma-5-mm-3D_gldm_DependenceEntropy	log-sigma-5-mm-3D	4.52014007634194 - 5.57366067984076	4.31821187577685 - 5.33464764456174
log-sigma-5-mm-3D_gldm_DependenceNonUniformity	log-sigma-5-mm-3D	289099.719313383 - 1788047.66349417	394003.367703206 - 3920289.62827826
log-sigma-5-mm-3D_gldm_DependenceNonUniformityNormalized	log-sigma-5-mm-3D	0.164886184434738 - 0.308675908310438	0.185535235243109 - 0.313454763099936
log-sigma-5-mm-3D_gldm_DependenceVariance	log-sigma-5-mm-3D	25.369062749718 - 34.9472437133642	24.5671183006574 - 36.0351092512846
log-sigma-5-mm-3D_gldm_GrayLevelNonUniformity	log-sigma-5-mm-3D	382830.175143461 - 2298215.11637832	597225.001637782 - 4244507.79417168
log-sigma-5-mm-3D_gldm_GrayLevelVariance	log-sigma-5-mm-3D	0.797885288277756 - 1.71468856075057	0.759264968692898 - 2.24389639706722
log-sigma-5-mm-3D_gldm_HighGrayLevelEmphasis	log-sigma-5-mm-3D	39.8360918513998 - 114.587369907895	29.782670377614 - 111.718771866115
log-sigma-5-mm-3D_gldm_LargeDependenceEmphasis	log-sigma-5-mm-3D	485.34874321842 - 547.518669742407	498.945546344799 - 560.968219581098
log-sigma-5-mm-3D_gldm_LargeDependenceHighGrayLevelEmphasis	log-sigma-5-mm-3D	20595.1055102552 - 61714.0998433713	15990.5411826555 - 60674.1563288841
log-sigma-5-mm-3D_gldm_LargeDependenceLowGrayLevelEmphasis	log-sigma-5-mm-3D	4.77779838004514 - 14.6319989679925	5.00663680069372 - 20.9761460415502
log-sigma-5-mm-3D_gldm_LowGrayLevelEmphasis	log-sigma-5-mm-3D	0.0094019632360717 - 0.0294753317781265	0.00990398468357575 - 0.0403554308791602
log-sigma-5-mm-3D_gldm_SmallDependenceEmphasis	log-sigma-5-mm-3D	0.002471591311005 - 0.00315950115939928	0.00236707547638727 - 0.00314182255596201
log-sigma-5-mm-3D_gldm_SmallDependenceHighGrayLevelEmphasis	log-sigma-5-mm-3D	0.109513104745535 - 0.308529829685964	0.0713910984364749 - 0.306781129705259
log-sigma-5-mm-3D_gldm_SmallDependenceLowGrayLevelEmphasis	log-sigma-5-mm-3D	2.94604674434739E-05 - 8.48233574823907E-05	2.98653044931888E-05 - 0.000105406067477602
log-sigma-5-mm-3D_glrlm_GrayLevelNonUniformity	log-sigma-5-mm-3D	52460.9652301598 - 302230.335619848	91160.9988211422 - 363240.514097703
log-sigma-5-mm-3D_glrlm_GrayLevelNonUniformityNormalized	log-sigma-5-mm-3D	0.181894339964593 - 0.285093476934922	0.171014744176227 - 0.290853804976594
log-sigma-5-mm-3D_glrlm_GrayLevelVariance	log-sigma-5-mm-3D	1.17268067893683 - 3.1349516467348	1.08354257520712 - 3.69438747042137
log-sigma-5-mm-3D_glrlm_HighGrayLevelRunEmphasis	log-sigma-5-mm-3D	39.5567649494656 - 108.629938754492	28.9597695617309 - 106.35424559934

log-sigma-5-mm-3D_glrlm_LongRunEmphasis	log-sigma-5-mm-3D	95.824640665124 - 439.369816185302	131.11120430309 - 678.384554599425
log-sigma-5-mm-3D_glrlm_LongRunHighGrayLevelEmphasis	log-sigma-5-mm-3D	4153.52565289595 - 34462.5390963942	6719.94979968914 - 76765.2060422844
log-sigma-5-mm-3D_glrlm_LongRunLowGrayLevelEmphasis	log-sigma-5-mm-3D	1.52666088818469 - 6.23524724924366	1.23187915727653 - 12.0357898629478
log-sigma-5-mm-3D_glrlm_LowGrayLevelRunEmphasis	log-sigma-5-mm-3D	0.0107972308374841 - 0.032888869564778	0.0111645297618735 - 0.0438950117779335
log-sigma-5-mm-3D_glrlm_RunEntropy	log-sigma-5-mm-3D	5.76827336912146 - 6.28745595358538	5.84152126107274 - 6.48701984410227
log-sigma-5-mm-3D_glrlm_RunLengthNonUniformity	log-sigma-5-mm-3D	22966.9290792791 - 193594.218390205	52836.7644816722 - 254109.977954785
log-sigma-5-mm-3D_glrlm_RunLengthNonUniformityNormalized	log-sigma-5-mm-3D	0.0988591738163161 - 0.143759768028317	0.0958223333858265 - 0.15871990744229
log-sigma-5-mm-3D_glrlm_RunPercentage	log-sigma-5-mm-3D	0.16080738177624 - 0.217604553804092	0.147679436388628 - 0.205107268297016
log-sigma-5-mm-3D_glrlm_RunVariance	log-sigma-5-mm-3D	60.8094004101176 - 336.721430488436	91.2271270595464 - 522.528138201822
log-sigma-5-mm-3D_glrlm_ShortRunEmphasis	log-sigma-5-mm-3D	0.191708953870635 - 0.351083246834091	0.187295404500949 - 0.351457470351837
log-sigma-5-mm-3D_glrlm_ShortRunHighGrayLevelEmphasis	log-sigma-5-mm-3D	8.7035747893984 - 29.5172817586165	5.4925007880023 - 33.389683341156
log-sigma-5-mm-3D_glrlm_ShortRunLowGrayLevelEmphasis	log-sigma-5-mm-3D	0.00349458478371366 - 0.00972304266797108	0.00343159316580472 - 0.0110820233336452
log-sigma-5-mm-3D_glszm_GrayLevelNonUniformity	log-sigma-5-mm-3D	17.4714285714286 - 84.4545454545455	18.35 - 78
log-sigma-5-mm-3D_glszm_GrayLevelNonUniformityNormalized	log-sigma-5-mm-3D	0.115651612232625 - 0.199868517165814	0.0819987386400058 - 0.193213296398892
log-sigma-5-mm-3D_glszm_GrayLevelVariance	log-sigma-5-mm-3D	4.7237796117478 - 13.237814133918	4.55264154972754 - 16.0859967599812
log-sigma-5-mm-3D_glszm_HighGrayLevelZoneEmphasis	log-sigma-5-mm-3D	40.1300448430493 - 107.2457002457	30.9161676646707 - 124.424124513619
log-sigma-5-mm-3D_glszm_LargeAreaEmphasis	log-sigma-5-mm-3D	2272798385.34752 - 38250102664.6995	4822052013.59447 - 109147325472.849
log-sigma-5-mm-3D_glszm_LargeAreaHighGrayLevelEmphasis	log-sigma-5-mm-3D	136315851760.901 - 3479035144217.08	208541054629.134 - 8607260536297.56

log-sigma-5-mm-3D_glszm_LargeAreaLowGrayLevelEmphasis	log-sigma-5-mm-3D	38573522.3511278 - 684235721.688867	95013807.7734995 - 1402374129.36
log-sigma-5-mm-3D_glszm_LowGrayLevelZoneEmphasis	log-sigma-5-mm-3D	0.0180795250619567 - 0.0791316627474792	0.0203688260369182 - 0.112752452141004
log-sigma-5-mm-3D_glszm_SizeZoneNonUniformity	log-sigma-5-mm-3D	1.28368794326241 - 3.68099173553719	1.37113402061856 - 2.81420765027322
log-sigma-5-mm-3D_glszm_SizeZoneNonUniformityNormalized	log-sigma-5-mm-3D	0.00544370963442007 - 0.0102168260273131	0.00489504891970273 - 0.0111007873245636
log-sigma-5-mm-3D_glszm_SmallAreaEmphasis	log-sigma-5-mm-3D	0.0102110151972388 - 0.0372278291491075	0.0117175587992492 - 0.0456320890807227
log-sigma-5-mm-3D_glszm_SmallAreaHighGrayLevelEmphasis	log-sigma-5-mm-3D	0.276416942825297 - 4.07167452987649	0.363268077662931 - 4.15974159924425
log-sigma-5-mm-3D_glszm_SmallAreaLowGrayLevelEmphasis	log-sigma-5-mm-3D	0.000156114493987597 - 0.00688636960134076	0.000160055036483909 - 0.01237353368592
log-sigma-5-mm-3D_glszm_ZoneEntropy	log-sigma-5-mm-3D	7.08642587408778 - 8.96341358418142	7.10392728083429 - 8.66222515609718
log-sigma-5-mm-3D_glszm_ZonePercentage	log-sigma-5-mm-3D	3.72766327332297E-05 - 0.000136219475842117	2.49903883121876E-05 - 0.000116311704065353
log-sigma-5-mm-3D_glszm_ZoneVariance	log-sigma-5-mm-3D	2204965287.46793 - 37530443848.1136	4726282429.42798 - 108298951365.261
log-sigma-5-mm-3D_ngtdm_Busyness	log-sigma-5-mm-3D	284.603544714983 - 2282.61515327114	387.315608738677 - 2897.1750060562
log-sigma-5-mm-3D_ngtdm_Coarseness	log-sigma-5-mm-3D	4.02748277742213E-06 - 2.56368357235904E-05	3.03309998831491E-06 - 1.48076477519432E-05
log-sigma-5-mm-3D_ngtdm_Complexity	log-sigma-5-mm-3D	6.09754579310015 - 15.750374959984	4.10539330945415 - 23.1738329312249
log-sigma-5-mm-3D_ngtdm_Contrast	log-sigma-5-mm-3D	0.00131649891763396 - 0.00355096346470269	0.00125255935761719 - 0.00411795206515003
log-sigma-5-mm-3D_ngtdm_Strength	log-sigma-5-mm-3D	0.000265858279886577 - 0.00236282889843582	0.000206592973983197 - 0.00192004993175156
wavelet-LLH_firstorder_Energy	wavelet-LLH	1166512817989.22 - 6833706649600.59	2150330759699.64 - 12914106778233.8
wavelet-LLH_firstorder_TotalEnergy	wavelet-LLH	1166512817989.22 - 6833706649600.59	2150330759699.64 - 12914106778233.8
wavelet-LLH_firstorder_Entropy	wavelet-LLH	2.28809657560053 - 3.59988835067815	1.68614073178307 - 3.39272941835723

wavelet-LLH_firstorder_Minimum	wavelet-LLH	-783.510687789161 - -470.2821099413	-794.230732133614 - -444.358575011019
wavelet-LLH_firstorder_10Percentile	wavelet-LLH	-95.4918361285997 - -22.06246058763	-88.3414211335721 - -11.0010486758898
wavelet-LLH_firstorder_90Percentile	wavelet-LLH	26.8846732850131 - 102.924167931895	11.6417286802193 - 105.802402409189
wavelet-LLH_firstorder_Maximum	wavelet-LLH	453.252236490388 - 835.138541878887	454.789877207946 - 780.608392122959
wavelet-LLH_firstorder_Mean	wavelet-LLH	-3.2762002523877 - 4.37932977832144	-1.20238599993185 - 4.11589763232045
wavelet-LLH_firstorder_Median	wavelet-LLH	-4.44089209850063E-14 - 1.54896172925893	-3.73034936274053E-14 - 0.848061157579775
wavelet-LLH_firstorder_InterquartileRange	wavelet-LLH	3.65936256712059 - 62.1378513348177	3.30871309831085 - 28.8309266172861
wavelet-LLH_firstorder_Range	wavelet-LLH	923.534346431688 - 1617.03218629424	899.148452218965 - 1537.35015924689
wavelet-LLH_firstorder_MeanAbsoluteDeviation	wavelet-LLH	21.0262643703205 - 56.5644571250936	9.43602813423517 - 54.8611210672196
wavelet-LLH_firstorder_RobustMeanAbsoluteDeviation	wavelet-LLH	3.07763781135836 - 28.3282851821151	2.0464838066308 - 20.3682883340837
wavelet-LLH_firstorder_RootMeanSquared	wavelet-LLH	999.533704125119 - 1008.52865628745	1000.42254922791 - 1008.92714094891
wavelet-LLH_firstorder_StandardDeviation	wavelet-LLH	52.8615714687457 - 101.663547844175	23.5873728015793 - 100.897567864391
wavelet-LLH_firstorder_Skewness	wavelet-LLH	-1.37943175821351 - 0.336176026769372	-1.4937087064916 - 0.310952080803565
wavelet-LLH_firstorder_Kurtosis	wavelet-LLH	7.51098126642821 - 20.5736956006443	8.34277385538595 - 31.4628815937736
wavelet-LLH_firstorder_Variance	wavelet-LLH	2794.34573814531 - 10335.4769602648	556.364155680684 - 10180.3192009493
wavelet-LLH_firstorder_Uniformity	wavelet-LLH	0.131724276978724 - 0.32629258113107	0.18118279166916 - 0.402352861370771
wavelet-LLH_glcm_Autocorrelation	wavelet-LLH	383.393429422261 - 1064.00037187192	342.363569291218 - 1062.71625116944
wavelet-LLH_glcm_ClusterProminence	wavelet-LLH	1814.90603975254 - 15329.9732014521	159.968960581076 - 14786.4598634878

wavelet-LLH_glcm_ClusterShade	wavelet-LLH	-213.308681382784 - 20.087173574035	-132.177822150043 - 19.2725078031571
wavelet-LLH_glcm_ClusterTendency	wavelet-LLH	10.5654279302389 - 34.4860821253319	2.4940761530391 - 34.236125477954
wavelet-LLH_glcm_Contrast	wavelet-LLH	8.12647058873751 - 31.4093789231351	1.83111469181877 - 30.4085534176834
wavelet-LLH_glcm_Correlation	wavelet-LLH	0.0184525682917568 - 0.146189826232567	0.0262833494025462 - 0.180390476704906
wavelet-LLH_glcm_DifferenceAverage	wavelet-LLH	1.2908672840416 - 2.83746549806654	0.753272519118241 - 2.80504083842533
wavelet-LLH_glcm_DifferenceEntropy	wavelet-LLH	1.99665350030285 - 3.0197182025466	1.51492079535642 - 2.88460055804209
wavelet-LLH_glcm_DifferenceVariance	wavelet-LLH	6.28850418858783 - 22.408943962131	1.23740685628329 - 21.4153693195473
wavelet-LLH_glcm_Id	wavelet-LLH	0.501434196303628 - 0.676416782724108	0.537165907628807 - 0.70509072740716
wavelet-LLH_glcm_Idm	wavelet-LLH	0.444461547891068 - 0.649267424941414	0.496502302180382 - 0.69314354262655
wavelet-LLH_glcm_Idmn	wavelet-LLH	0.990193799808945 - 0.996735788770616	0.991654271020625 - 0.998707357815035
wavelet-LLH_glcm_Idn	wavelet-LLH	0.954904536021734 - 0.975455842197334	0.959581114713443 - 0.981547281651482
wavelet-LLH_glcm_Imc1	wavelet-LLH	-0.256569674680333 - -0.115866393667289	-0.24757516839175 - -0.105121886290746
wavelet-LLH_glcm_Imc2	wavelet-LLH	0.680373437125263 - 0.830623721114556	0.529988702230496 - 0.832685659619084
wavelet-LLH_glcm_InverseVariance	wavelet-LLH	0.280223292843917 - 0.43355711835032	0.271957531149201 - 0.474972713944177
wavelet-LLH_glcm_JointAverage	wavelet-LLH	19.5552311759036 - 32.6083050233718	18.4985898353918 - 32.584636398429
wavelet-LLH_glcm_JointEnergy	wavelet-LLH	0.0373482898628732 - 0.151788195894971	0.0685597661983429 - 0.188418827759565
wavelet-LLH_glcm_JointEntropy	wavelet-LLH	4.17237730418491 - 6.66095468748205	3.19654076705267 - 6.15810828368661
wavelet-LLH_glcm_MCC	wavelet-LLH	0.601964457449912 - 0.777980358443854	0.582063107243434 - 0.787465058063668

wavelet-LLH_glcm_MaximumProbability	wavelet-LLH	0.124661123576703 - 0.345888672596658	0.153838574049015 - 0.323577692035447
wavelet-LLH_glcm_SumAverage	wavelet-LLH	39.1104623518073 - 65.2166100467437	36.9971796707836 - 65.1692727968581
wavelet-LLH_glcm_SumEntropy	wavelet-LLH	2.7923980224911 - 4.0722085708705	2.20350083998506 - 3.87170876846641
wavelet-LLH_glcm_SumSquares	wavelet-LLH	4.67297462974411 - 16.4738652621167	1.08129771121447 - 16.1611697239093
wavelet-LLH_gldm_DependenceEntropy	wavelet-LLH	6.04399716447022 - 7.13698240050829	5.44946316934952 - 6.95260839705432
wavelet-LLH_gldm_DependenceNonUniformity	wavelet-LLH	86599.8874921639 - 417139.558765539	120277.509430195 - 900708.543889578
wavelet-LLH_gldm_DependenceNonUniformityNormalized	wavelet-LLH	0.0467665797732238 - 0.0838757666601754	0.0482285003800511 - 0.080789658732852
wavelet-LLH_gldm_DependenceVariance	wavelet-LLH	22.5900616213275 - 80.5064327609466	19.7790496118764 - 80.6547366630309
wavelet-LLH_gldm_GrayLevelNonUniformity	wavelet-LLH	287848.452947472 - 1685588.40210464	384760.501119794 - 5191639.44083933
wavelet-LLH_gldm_GrayLevelVariance	wavelet-LLH	4.65969956788539 - 16.70179789502	1.08035123069379 - 16.4160567347956
wavelet-LLH_gldm_HighGrayLevelEmphasis	wavelet-LLH	389.913755991285 - 1082.15572677392	343.33046019592 - 1080.94874844839
wavelet-LLH_gldm_LargeDependenceEmphasis	wavelet-LLH	78.4775280624337 - 254.806085693537	101.97138543721 - 249.534508056342
wavelet-LLH_gldm_LargeDependenceHighGrayLevelEmphasis	wavelet-LLH	59485.77612995 - 191624.745100661	62452.8738218426 - 192353.311622453
wavelet-LLH_gldm_LargeDependenceLowGrayLevelEmphasis	wavelet-LLH	0.0742311184974978 - 0.447090619786791	0.0969103172450846 - 0.536969530869842
wavelet-LLH_gldm_LowGrayLevelEmphasis	wavelet-LLH	0.000979932024977883 - 0.00278406550293829	0.00103020945136845 - 0.00295505464691793
wavelet-LLH_gldm_SmallDependenceEmphasis	wavelet-LLH	0.0407844572518271 - 0.116684012730096	0.0228831946906603 - 0.113596367447085

wavelet-LLH_gldm_SmallDependenceHighGrayLevelEmphasis	wavelet-LLH	23.2491171692133 - 122.343348117067	7.98962924207087 - 123.17401526014
wavelet-LLH_gldm_SmallDependenceLowGrayLevelEmphasis	wavelet-LLH	7.12484123137829E-05 - 0.000208607017273984	6.67455043900136E-05 - 0.000184525873300695
wavelet-LLH_glrlm_GrayLevelNonUniformity	wavelet-LLH	106710.844902433 - 720208.772922409	196070.44749201 - 2521987.64329738
wavelet-LLH_glrlm_GrayLevelNonUniformityNormalized	wavelet-LLH	0.0946047068129941 - 0.252818048706202	0.106666006191955 - 0.351127835495617
wavelet-LLH_glrlm_GrayLevelVariance	wavelet-LLH	7.40292695095193 - 23.0175917100294	1.64861104930691 - 21.7606502066938
wavelet-LLH_glrlm_HighGrayLevelRunEmphasis	wavelet-LLH	394.80466635937 - 1090.13302296945	344.082444724711 - 1086.36903762236
wavelet-LLH_glrlm_LongRunEmphasis	wavelet-LLH	2.65093676976493 - 48.9138954819034	3.77932911216728 - 43.5817467279153
wavelet-LLH_glrlm_LongRunHighGrayLevelEmphasis	wavelet-LLH	2822.4235882632 - 33650.8861760484	2620.73449444473 - 31833.7735189338
wavelet-LLH_glrlm_LongRunLowGrayLevelEmphasis	wavelet-LLH	0.00255360512871431 - 0.0848365670413601	0.00370666511168442 - 0.0821874650264671
wavelet-LLH_glrlm_LowGrayLevelRunEmphasis	wavelet-LLH	0.000990872002277202 - 0.00284097803546073	0.0010604699816663 - 0.00297233360999644
wavelet-LLH_glrlm_RunEntropy	wavelet-LLH	4.27170687760114 - 5.06371818755761	3.69291030188398 - 5.0873241398951
wavelet-LLH_glrlm_RunLengthNonUniformity	wavelet-LLH	390861.645164666 - 2572215.0457561	835070.704155393 - 3020892.84993096
wavelet-LLH_glrlm_RunLengthNonUniformityNormalized	wavelet-LLH	0.467625933354056 - 0.703584789537354	0.408485263328506 - 0.610882043708785
wavelet-LLH_glrlm_RunPercentage	wavelet-LLH	0.529542483660131 - 0.750931075858507	0.523117251836975 - 0.710838236826133
wavelet-LLH_glrlm_RunVariance	wavelet-LLH	0.799178502640638 - 44.7657440213978	1.64195388594696 - 39.0157278594611
wavelet-LLH_glrlm_ShortRunEmphasis	wavelet-LLH	0.703630659399124 - 0.858271684918261	0.649671477069015 - 0.80575880502695
wavelet-LLH_glrlm_ShortRunHighGrayLevelEmphasis	wavelet-LLH	309.326983217594 - 888.884416020202	223.845036710281 - 878.559472722939

wavelet-LLH_glrlm_ShortRunLowGrayLevelEmphasis	wavelet-LLH	0.000824615519729081 - 0.00224480691697798	0.000850835544212678 - 0.00194468799502499
wavelet-LLH_glszm_GrayLevelNonUniformity	wavelet-LLH	4409.07342201871 - 26945.408386712	7065.1597021044 - 19060.6396075629
wavelet-LLH_glszm_GrayLevelNonUniformityNormalized	wavelet-LLH	0.0348490857277109 - 0.0581625650238162	0.0353920146282957 - 0.0898621296319576
wavelet-LLH_glszm_GrayLevelVariance	wavelet-LLH	23.178587171344 - 69.1498511321684	15.0150214608685 - 65.6073534434876
wavelet-LLH_glszm_HighGrayLevelZoneEmphasis	wavelet-LLH	395.915325416048 - 1102.95328595877	352.578457105361 - 1089.43117173717
wavelet-LLH_glszm_LargeAreaEmphasis	wavelet-LLH	2143876.72468609 - 39776202.418196	2729516.69064151 - 316552796.915152
wavelet-LLH_glszm_LargeAreaHighGrayLevelEmphasis	wavelet-LLH	2274086957.29807 - 28210396146.5401	2708586104.61115 - 108308811696.488
wavelet-LLH_glszm_LargeAreaLowGrayLevelEmphasis	wavelet-LLH	2023.74229034535 - 73624.8431834084	2578.50801510838 - 927935.527570277
wavelet-LLH_glszm_LowGrayLevelZoneEmphasis	wavelet-LLH	0.00115250396666468 - 0.00343686368269992	0.00131523552109293 - 0.00352172175405619
wavelet-LLH_glszm_SizeZoneNonUniformity	wavelet-LLH	23536.8350767773 - 156819.77660273	43511.6886171085 - 131396.409005263
wavelet-LLH_glszm_SizeZoneNonUniformityNormalized	wavelet-LLH	0.194774521236574 - 0.356271595231766	0.213597158701458 - 0.347108816476548
wavelet-LLH_glszm_SmallAreaEmphasis	wavelet-LLH	0.444251569623574 - 0.618508918807942	0.467147566975189 - 0.610225113307403
wavelet-LLH_glszm_SmallAreaHighGrayLevelEmphasis	wavelet-LLH	186.857462442947 - 669.410811856032	193.339384579931 - 647.720079492577
wavelet-LLH_glszm_SmallAreaLowGrayLevelEmphasis	wavelet-LLH	0.000723868113457928 - 0.00165353926957688	0.000784375482115314 - 0.00197677318510376
wavelet-LLH_glszm_ZoneEntropy	wavelet-LLH	6.9156793833325 - 8.17173856127695	6.46233427943898 - 7.81268867027484
wavelet-LLH_glszm_ZonePercentage	wavelet-LLH	0.0333399741358219 - 0.120677730787304	0.0162987475974952 - 0.120375079346244
wavelet-LLH_glszm_ZoneVariance	wavelet-LLH	2143801.8769138 - 39775482.5815587	2729447.67828977 - 316549032.551846
wavelet-LLH_ngtdm_Busyness	wavelet-LLH	58.9892810041448 - 554.179404617164	86.7232323238969 - 2024.01070549345

wavelet-LLH_ngtdm_Coarseness	wavelet-LLH	1.02771455072697E-06 - 7.68848053941105E-06	3.9017391943509E-07 - 3.39208175054085E-06
wavelet-LLH_ngtdm_Complexity	wavelet-LLH	932.124788845119 - 3686.10651862367	434.010519430338 - 3800.17370321273
wavelet-LLH_ngtdm_Contrast	wavelet-LLH	0.00453505464233653 - 0.0272933741587666	0.00105147209727077 - 0.02051736562005
wavelet-LLH_ngtdm_Strength	wavelet-LLH	0.00147480484712154 - 0.0167504325655479	0.00102040101945944 - 0.00993399854997122
wavelet-LHL_firstorder_Energy	wavelet-LHL	1161998598941.43 - 6762871397128.3	2123925126974.02 - 12912829316704.3
wavelet-LHL_firstorder_TotalEnergy	wavelet-LHL	1161998598941.43 - 6762871397128.3	2123925126974.02 - 12912829316704.3
wavelet-LHL_firstorder_Entropy	wavelet-LHL	1.0524138437602 - 2.03743412534067	1.11571630387518 - 1.82045339908856
wavelet-LHL_firstorder_Minimum	wavelet-LHL	-474.387935654689 - -143.193771139555	-551.68262956228 - -127.282947762468
wavelet-LHL_firstorder_10Percentile	wavelet-LHL	-25.5966848508946 - -3.8981175662865	-20.0136379983406 - -3.8702131879624
wavelet-LHL_firstorder_90Percentile	wavelet-LHL	3.95754189002195 - 26.0772484592361	4.13876663589189 - 20.3763594937522
wavelet-LHL_firstorder_Maximum	wavelet-LHL	116.605343768635 - 410.921654100353	134.959427803259 - 603.395600891481
wavelet-LHL_firstorder_Mean	wavelet-LHL	-0.251017197227259 - 0.0751385851540905	-0.0371558236087264 - 0.0627875983595642
wavelet-LHL_firstorder_Median	wavelet-LHL	-0.0128245021455236 - 0.0770825774841598	-4.08224039257782E-14 - 0.0693996585383753
wavelet-LHL_firstorder_InterquartileRange	wavelet-LHL	1.12587475117201 - 21.1519735964766	1.50260189422505 - 14.7560232956311
wavelet-LHL_firstorder_Range	wavelet-LHL	277.998548916049 - 885.309589755042	279.483244859199 - 1094.34603518511
wavelet-LHL_firstorder_MeanAbsoluteDeviation	wavelet-LHL	2.92967241578783 - 17.2701789446288	3.17211838850463 - 13.5470821208563
wavelet-LHL_firstorder_RobustMeanAbsoluteDeviation	wavelet-LHL	0.818290381380159 - 9.27153957654439	0.832055558409332 - 6.6168243873867
wavelet-LHL_firstorder_RootMeanSquared	wavelet-LHL	999.889324611069 - 1000.3238933024	1000.02398809271 - 1000.37306717901

wavelet-LHL_firstorder_StandardDeviation	wavelet-LHL	5.88932751897498 - 27.1006013087019	7.02573156082922 - 27.3583156052798
wavelet-LHL_firstorder_Skewness	wavelet-LHL	-1.60471508635831 - 0.177126700162212	-1.51508239579374 - 0.252564248054072
wavelet-LHL_firstorder_Kurtosis	wavelet-LHL	11.6167069585687 - 75.3385056951377	15.8281829021448 - 87.5110326826486
wavelet-LHL_firstorder_Variance	wavelet-LHL	34.684178625756 - 734.442591293212	49.3609039648318 - 748.477432758098
wavelet-LHL_firstorder_Uniformity	wavelet-LHL	0.323754034029287 - 0.506134722292511	0.365705328593971 - 0.504257348425695
wavelet-LHL_glcm_Autocorrelation	wavelet-LHL	42.1984318861498 - 380.399332709586	42.1944880303982 - 551.946364745322
wavelet-LHL_glcm_ClusterProminence	wavelet-LHL	1.18213071639426 - 186.634809412067	1.77131367619026 - 378.640471520816
wavelet-LHL_glcm_ClusterShade	wavelet-LHL	-2.37846358159119 - 0.399768386615949	-4.01903613950815 - 0.368239737486219
wavelet-LHL_glcm_ClusterTendency	wavelet-LHL	0.679752403622626 - 2.9599665514154	0.700038158846625 - 3.33217082551527
wavelet-LHL_glcm_Contrast	wavelet-LHL	0.370120064675574 - 2.13999567772836	0.406479730469674 - 2.30738372367549
wavelet-LHL_glcm_Correlation	wavelet-LHL	0.0894225691457765 - 0.385275624442603	0.102253498290874 - 0.335840918864988
wavelet-LHL_glcm_DifferenceAverage	wavelet-LHL	0.358245617099679 - 0.938980150704596	0.365542064740237 - 0.834935798861161
wavelet-LHL_glcm_DifferenceEntropy	wavelet-LHL	0.95654554307537 - 1.77865623480147	1.00319384702205 - 1.62487427961615
wavelet-LHL_glcm_DifferenceVariance	wavelet-LHL	0.236582102398788 - 1.31602639863678	0.265150202227536 - 1.67010200371075
wavelet-LHL_glcm_Id	wavelet-LHL	0.649650806458758 - 0.826958848798486	0.669960140869634 - 0.822660360507773
wavelet-LHL_glcm_Idm	wavelet-LHL	0.625403368066274 - 0.823607778080631	0.651837396565139 - 0.82105602640943
wavelet-LHL_glcm_Idmn	wavelet-LHL	0.996719803232794 - 0.99926132488233	0.99669134686975 - 0.999240601210882
wavelet-LHL_glcm_Idn	wavelet-LHL	0.965877677326937 - 0.985520020185169	0.965889919828756 - 0.9852029760277

wavelet-LHL_glcm_Imc1	wavelet-LHL	-0.134094614380014 - -0.0495451272405381	-0.122197346193976 - -0.050431993592785
wavelet-LHL_glcm_Imc2	wavelet-LHL	0.269021519821733 - 0.520899412853314	0.273292485637381 - 0.582060072406574
wavelet-LHL_glcm_InverseVariance	wavelet-LHL	0.317322385482082 - 0.503526121535385	0.338687956997209 - 0.502261055864996
wavelet-LHL_glcm_JointAverage	wavelet-LHL	6.49204619707374 - 19.4989932658689	6.49150334176029 - 23.4881188991257
wavelet-LHL_glcm_JointEnergy	wavelet-LHL	0.120936714969445 - 0.286598569739787	0.153163170115645 - 0.284047581723885
wavelet-LHL_glcm_JointEntropy	wavelet-LHL	2.01315900486079 - 3.94604826758997	2.13142567383845 - 3.5245591075428
wavelet-LHL_glcm_MCC	wavelet-LHL	0.321159341100689 - 0.646993162101368	0.314082678982969 - 0.641309453383256
wavelet-LHL_glcm_MaximumProbability	wavelet-LHL	0.188505012546871 - 0.452185995730166	0.224663135763808 - 0.430369924885481
wavelet-LHL_glcm_SumAverage	wavelet-LHL	12.9840923941475 - 38.9979865317379	12.9830066835206 - 46.9762377982513
wavelet-LHL_glcm_SumEntropy	wavelet-LHL	1.62428939839062 - 2.59553976453916	1.69473115750491 - 2.35618824481537
wavelet-LHL_glcm_SumSquares	wavelet-LHL	0.26246811707455 - 1.27499055728594	0.295146847876623 - 1.40348845142814
wavelet-LHL_gldm_DependenceEntropy	wavelet-LHL	4.90465641702243 - 5.71474508599187	4.89903640584154 - 5.64227282280941
wavelet-LHL_gldm_DependenceNonUniformity	wavelet-LHL	88233.3105258988 - 479524.245446944	148161.947468549 - 831785.746244962
wavelet-LHL_gldm_DependenceNonUniformityNormalized	wavelet-LHL	0.061327394902473 - 0.0915260497348084	0.0644635242610331 - 0.0867071128921359
wavelet-LHL_gldm_DependenceVariance	wavelet-LHL	14.7642350137541 - 58.2761553072168	14.3492181506396 - 39.3392050238686
wavelet-LHL_gldm_GrayLevelNonUniformity	wavelet-LHL	466351.969282383 - 3281431.95813533	776613.298623472 - 4956121.45916873
wavelet-LHL_gldm_GrayLevelVariance	wavelet-LHL	0.262866823367221 - 1.27830904402253	0.294634920063986 - 1.39134810429195
wavelet-LHL_gldm_HighGrayLevelEmphasis	wavelet-LHL	42.4284221787607 - 381.099892274785	42.4275275395751 - 552.871278252761

wavelet-LHL_gldm_LargeDependenceEmphasis	wavelet-LHL	126.026058846814 - 349.214614037336	136.697795822573 - 335.438380221654
wavelet-LHL_gldm_LargeDependenceHighGrayLevelEmphasis	wavelet-LHL	10617.8776447186 - 72859.6832958749	10505.2580676329 - 107282.234933653
wavelet-LHL_gldm_LargeDependenceLowGrayLevelEmphasis	wavelet-LHL	0.413065918300411 - 6.16901759593512	0.331927446493338 - 6.41899248882777
wavelet-LHL_gldm_LowGrayLevelEmphasis	wavelet-LHL	0.00265464279318623 - 0.0242432266571639	0.00182716775202276 - 0.0242555559338846
wavelet-LHL_gldm_SmallDependenceEmphasis	wavelet-LHL	0.00539179527395861 - 0.0388528598475882	0.00622443971295635 - 0.0329764705687517
wavelet-LHL_gldm_SmallDependenceHighGrayLevelEmphasis	wavelet-LHL	0.303917992951075 - 12.0464168035048	0.327896434125708 - 13.938735052287
wavelet-LHL_gldm_SmallDependenceLowGrayLevelEmphasis	wavelet-LHL	4.51561557624459E-05 - 0.000207872020298664	4.53479589427014E-05 - 0.000213829404373775
wavelet-LHL_glrlm_GrayLevelNonUniformity	wavelet-LHL	168274.962454463 - 1401913.43513047	421962.84417983 - 2313368.11547418
wavelet-LHL_glrlm_GrayLevelNonUniformityNormalized	wavelet-LHL	0.277611289560986 - 0.482246975885017	0.319867414373804 - 0.468436259370091
wavelet-LHL_glrlm_GrayLevelVariance	wavelet-LHL	0.294842697595049 - 1.89960107065696	0.334892073135118 - 2.20823858731979
wavelet-LHL_glrlm_HighGrayLevelRunEmphasis	wavelet-LHL	42.5420866987878 - 381.389535532119	42.5616939244429 - 553.911014117641
wavelet-LHL_glrlm_LongRunEmphasis	wavelet-LHL	4.1109483058053 - 63.6997830352171	4.5378623726158 - 53.3435586866604
wavelet-LHL_glrlm_LongRunHighGrayLevelEmphasis	wavelet-LHL	428.298223067233 - 8137.43661492761	411.205544921426 - 8563.09759048501
wavelet-LHL_glrlm_LongRunLowGrayLevelEmphasis	wavelet-LHL	0.0135339409317533 - 0.81802943488143	0.0109003109888224 - 1.06672981405002
wavelet-LHL_glrlm_LowGrayLevelRunEmphasis	wavelet-LHL	0.00267023920405218 - 0.0242800516333974	0.00183518657528705 - 0.0243193769093272
wavelet-LHL_glrlm_RunEntropy	wavelet-LHL	3.38529620173769 - 3.82684459946424	3.36161883162133 - 3.86643824825336

wavelet-LHL_glrlm_RunLengthNonUniformity	wavelet-LHL	257614.404795355 - 970985.819212299	478457.372436913 - 2968157.08881767
wavelet-LHL_glrlm_RunLengthNonUniformityNormalized	wavelet-LHL	0.274302077620696 - 0.492224734204984	0.286741597723966 - 0.463926968276292
wavelet-LHL_glrlm_RunPercentage	wavelet-LHL	0.363365947350953 - 0.640979686335274	0.371981620839752 - 0.61855386481588
wavelet-LHL_glrlm_RunVariance	wavelet-LHL	1.57380461433045 - 54.2495030555517	1.65053860803931 - 44.5660157824448
wavelet-LHL_glrlm_ShortRunEmphasis	wavelet-LHL	0.480164548543756 - 0.724075075713232	0.50424640295168 - 0.699368834500083
wavelet-LHL_glrlm_ShortRunHighGrayLevelEmphasis	wavelet-LHL	22.1142810463364 - 238.090320718004	22.0093732562291 - 351.971316633714
wavelet-LHL_glrlm_ShortRunLowGrayLevelEmphasis	wavelet-LHL	0.00167772706545115 - 0.012648316052971	0.00117135922124418 - 0.012785940893178
wavelet-LHL_glszm_GrayLevelNonUniformity	wavelet-LHL	2128.47876121171 - 9493.37463709209	2652.79655407379 - 18650.1325973021
wavelet-LHL_glszm_GrayLevelNonUniformityNormalized	wavelet-LHL	0.0846527140105665 - 0.366477197730149	0.0669681120407617 - 0.328336042082732
wavelet-LHL_glszm_GrayLevelVariance	wavelet-LHL	2.84652308761062 - 16.0076334052351	3.13639277801048 - 23.0583339783451
wavelet-LHL_glszm_HighGrayLevelZoneEmphasis	wavelet-LHL	45.9478941684665 - 384.872458942262	45.2886111621638 - 569.68705450159
wavelet-LHL_glszm_LargeAreaEmphasis	wavelet-LHL	16065064.9645374 - 2803677138.02335	29194804.770291 - 1587267221.89565
wavelet-LHL_glszm_LargeAreaHighGrayLevelEmphasis	wavelet-LHL	4754578953.20286 - 153576346845.125	7970614971.06191 - 186905088743.009
wavelet-LHL_glszm_LargeAreaLowGrayLevelEmphasis	wavelet-LHL	52564.1776218179 - 52744066.4161925	69567.1989906109 - 26126806.3410023
wavelet-LHL_glszm_LowGrayLevelZoneEmphasis	wavelet-LHL	0.00324201280472756 - 0.0312107626975804	0.00208325458638222 - 0.0321115467600893
wavelet-LHL_glszm_SizeZoneNonUniformity	wavelet-LHL	836.930275850398 - 14767.6839340988	1388.37739706025 - 65969.0143042106
wavelet-LHL_glszm_SizeZoneNonUniformityNormalized	wavelet-LHL	0.109317654636703 - 0.299465671516665	0.11359949771359 - 0.305901977452422
wavelet-LHL_glszm_SmallAreaEmphasis	wavelet-LHL	0.290623470928089 - 0.560671979100579	0.297566640559705 - 0.568973758112514

wavelet-LHL_glszm_SmallAreaHighGrayLevelEmphasis	wavelet-LHL	17.3489150884395 - 200.392791920217	16.5394215849707 - 309.136446144501
wavelet-LHL_glszm_SmallAreaLowGrayLevelEmphasis	wavelet-LHL	0.00175243385343762 - 0.012561126258144	0.00115683492845256 - 0.01250426193291
wavelet-LHL_glszm_ZoneEntropy	wavelet-LHL	5.2659658787506 - 6.89331029956284	5.34730213232901 - 6.83502778905079
wavelet-LHL_glszm_ZonePercentage	wavelet-LHL	0.00103264478697005 - 0.0319871428361994	0.00179528852539233 - 0.0266476235682359
wavelet-LHL_glszm_ZoneVariance	wavelet-LHL	16064087.6168252 - 2802739364.24269	29193396.5096858 - 1586956957.82236
wavelet-LHL_ngtdm_Busyness	wavelet-LHL	173.012725727051 - 9306.54815685436	322.563667346655 - 9216.49993201803
wavelet-LHL_ngtdm_Coarseness	wavelet-LHL	7.48926645220625E-07 - 6.23006037298475E-06	4.20152263413065E-07 - 2.47414924507995E-06
wavelet-LHL_ngtdm_Complexity	wavelet-LHL	25.3526701420912 - 376.614445139144	30.1651116875378 - 623.674889832361
wavelet-LHL_ngtdm_Contrast	wavelet-LHL	0.000423142212677014 - 0.0022709919332701	0.000423573991961517 - 0.00192506831337787
wavelet-LHL_ngtdm_Strength	wavelet-LHL	0.00011389871375489 - 0.00767827580983584	0.000114474495475196 - 0.00473314773833327
wavelet-LHH_firstorder_Energy	wavelet-LHH	1161702611387.68 - 6762839030818.77	2123979216717.3 - 12905409329935.7
wavelet-LHH_firstorder_TotalEnergy	wavelet-LHH	1161702611387.68 - 6762839030818.77	2123979216717.3 - 12905409329935.7
wavelet-LHH_firstorder_Entropy	wavelet-LHH	1.04409243063975 - 1.88512643234305	1.09489498263595 - 1.75431919304462
wavelet-LHH_firstorder_Minimum	wavelet-LHH	-364.345114066564 - -113.437709400442	-411.370517837409 - -113.13446680045
wavelet-LHH_firstorder_10Percentile	wavelet-LHH	-23.5122263855319 - -3.69944587872103	-19.1867569880698 - -3.79986026866154
wavelet-LHH_firstorder_90Percentile	wavelet-LHH	3.70177668991909 - 23.2453826088383	3.746294251472 - 18.9372733823281
wavelet-LHH_firstorder_Maximum	wavelet-LHH	110.637006724 - 398.912148614046	111.425318487778 - 412.793083279262
wavelet-LHH_firstorder_Mean	wavelet-LHH	-0.00723753678121619 - 0.00856051695457873	-0.00709332894550927 - 0.000653615268836051

wavelet-LHH_firstorder_Median	wavelet-LHH	-0.00764830761008206 - 0.0100171487024957	-0.0121267873829996 - 0.00809411033495147
wavelet-LHH_firstorder_InterquartileRange	wavelet-LHH	1.08251701355846 - 19.553667486808	1.43401589598233 - 14.317886077838
wavelet-LHH_firstorder_Range	wavelet-LHH	226.973065516066 - 732.262387041341	238.960932865846 - 809.039927272611
wavelet-LHH_firstorder_MeanAbsoluteDeviation	wavelet-LHH	2.67837983419891 - 15.1045142437173	2.87849821500156 - 12.801575170453
wavelet-LHH_firstorder_RobustMeanAbsoluteDeviation	wavelet-LHH	0.778254559954191 - 8.49209268232536	0.789657241313655 - 6.36019371994608
wavelet-LHH_firstorder_RootMeanSquared	wavelet-LHH	1000.0159384914 - 1000.25258438002	1000.01915119065 - 1000.237074003
wavelet-LHH_firstorder_StandardDeviation	wavelet-LHH	5.67915632652247 - 22.7970958696011	6.19261048459505 - 21.8324039176163
wavelet-LHH_firstorder_Skewness	wavelet-LHH	-0.000532303238106253 - 0.357605063479793	-0.162549628336962 - 0.350032359014991
wavelet-LHH_firstorder_Kurtosis	wavelet-LHH	10.0317734813256 - 43.0559565053855	14.1265426192682 - 56.9493275748709
wavelet-LHH_firstorder_Variance	wavelet-LHH	32.2528165810802 - 519.707580087783	38.3484246139165 - 476.653860821948
wavelet-LHH_firstorder_Uniformity	wavelet-LHH	0.345524254042284 - 0.508507290478506	0.376822923792014 - 0.504628362343735
wavelet-LHH_glcm_Autocorrelation	wavelet-LHH	29.3130531688093 - 240.214578611668	30.1655789111401 - 306.162553545959
wavelet-LHH_glcm_ClusterProminence	wavelet-LHH	0.758294189107845 - 37.9422776575447	0.848374695891929 - 47.7174866536135
wavelet-LHH_glcm_ClusterShade	wavelet-LHH	-0.185125463566301 - 0.920818839809373	-0.263198904340067 - 0.406041115594284
wavelet-LHH_glcm_ClusterTendency	wavelet-LHH	0.538161772771603 - 1.81269878530989	0.549786741726198 - 1.71061760660644
wavelet-LHH_glcm_Contrast	wavelet-LHH	0.457614607308865 - 1.93086077896675	0.500182327404523 - 1.8536519476574
wavelet-LHH_glcm_Correlation	wavelet-LHH	-0.0411449435566762 - 0.122997725175217	-0.0404014748156671 - 0.120505082394676
wavelet-LHH_glcm_DifferenceAverage	wavelet-LHH	0.435006063948835 - 0.920585469983887	0.438818063687711 - 0.844882637492397

wavelet-LHH_glcm_DifferenceEntropy	wavelet-LHH	1.01601962175167 - 1.74690268735608	1.06323104035794 - 1.64393853126633
wavelet-LHH_glcm_DifferenceVariance	wavelet-LHH	0.258858926147021 - 1.0658170896863	0.272934009595471 - 1.12147142224581
wavelet-LHH_glcm_Id	wavelet-LHH	0.647690712871746 - 0.794997046098374	0.669607448444861 - 0.789541720386674
wavelet-LHH_glcm_Idm	wavelet-LHH	0.624043079414762 - 0.790409611980826	0.651295252394219 - 0.787053297968349
wavelet-LHH_glcm_Idmn	wavelet-LHH	0.994158997782277 - 0.998604388098647	0.99535815222818 - 0.998878689690827
wavelet-LHH_glcm_Idn	wavelet-LHH	0.951310584438419 - 0.978931514174767	0.956026554303543 - 0.980570911216405
wavelet-LHH_glcm_Imc1	wavelet-LHH	-0.0840851493604323 - - 0.0362666392355184	-0.0836408448332102 - - 0.0378787218597156
wavelet-LHH_glcm_Imc2	wavelet-LHH	0.207158032038293 - 0.445461188479646	0.215103905689752 - 0.460324137198547
wavelet-LHH_glcm_InverseVariance	wavelet-LHH	0.370284661879706 - 0.503456254393735	0.387065020632991 - 0.505360587452308
wavelet-LHH_glcm_JointAverage	wavelet-LHH	5.41131304640471 - 15.4998576911023	5.49267686951307 - 17.4977350371656
wavelet-LHH_glcm_JointEnergy	wavelet-LHH	0.133484831912108 - 0.279078272133028	0.160246550817455 - 0.273553521372106
wavelet-LHH_glcm_JointEntropy	wavelet-LHH	2.03241225941954 - 3.67880681503063	2.12770131162113 - 3.40386141099617
wavelet-LHH_glcm_MCC	wavelet-LHH	0.215499065839085 - 0.503185375514775	0.228929689057305 - 0.529374533353397
wavelet-LHH_glcm_MaximumProbability	wavelet-LHH	0.198783325549079 - 0.430636904518025	0.228932001258201 - 0.409245884872071
wavelet-LHH_glcm_SumAverage	wavelet-LHH	10.8226260928094 - 30.9997153822045	10.9853537390261 - 34.9954700743311
wavelet-LHH_glcm_SumEntropy	wavelet-LHH	1.5405405420602 - 2.33679095535941	1.55788772598439 - 2.20427151078102
wavelet-LHH_glcm_SumSquares	wavelet-LHH	0.25955139807457 - 0.93588989106916	0.277862812319384 - 0.891067388565961
wavelet-LHH_gldm_DependenceEntropy	wavelet-LHH	4.56799406390038 - 5.30235010377214	4.55474707192328 - 5.13386447353716

wavelet-LHH_gldm_DependenceNonUniformity	wavelet-LHH	96915.0493520987 - 689104.168715879	177367.450663118 - 1462035.16665153
wavelet-LHH_gldm_DependenceNonUniformityNormalized	wavelet-LHH	0.0735027904368756 - 0.108553608479468	0.0835219045844319 - 0.116390976970307
wavelet-LHH_gldm_DependenceVariance	wavelet-LHH	9.92811043802597 - 54.8423771210534	9.59653714517022 - 42.1504386431523
wavelet-LHH_gldm_GrayLevelNonUniformity	wavelet-LHH	490678.037832131 - 3318181.87073315	800222.668256417 - 5762922.56348394
wavelet-LHH_gldm_GrayLevelVariance	wavelet-LHH	0.260059562455494 - 0.939420375080248	0.278068518793905 - 0.892385124658977
wavelet-LHH_gldm_HighGrayLevelEmphasis	wavelet-LHH	29.5369679493901 - 241.035251393386	30.4427890610635 - 306.847295539829
wavelet-LHH_gldm_LargeDependenceEmphasis	wavelet-LHH	120.44796049513 - 303.032937758982	135.690121133695 - 291.463468899922
wavelet-LHH_gldm_LargeDependenceHighGrayLevelEmphasis	wavelet-LHH	5577.53594303194 - 37908.8689041823	5624.22960851178 - 50753.5583616763
wavelet-LHH_gldm_LargeDependenceLowGrayLevelEmphasis	wavelet-LHH	0.598399333172534 - 9.87426230433898	0.50629103232113 - 6.36430546242907
wavelet-LHH_gldm_LowGrayLevelEmphasis	wavelet-LHH	0.00420916645731523 - 0.0350531139784942	0.00329064154817022 - 0.0341083016780878
wavelet-LHH_gldm_SmallDependenceEmphasis	wavelet-LHH	0.00681681885632287 - 0.0462306821851932	0.00782868632839748 - 0.0373858861343859
wavelet-LHH_gldm_SmallDependenceHighGrayLevelEmphasis	wavelet-LHH	0.210192153405614 - 8.08318489943894	0.285219817122938 - 10.5228205673828
wavelet-LHH_gldm_SmallDependenceLowGrayLevelEmphasis	wavelet-LHH	8.99990278403789E-05 - 0.000380836865239589	5.59916464764972E-05 - 0.00033559793878501
wavelet-LHH_glrlm_GrayLevelNonUniformity	wavelet-LHH	186173.13604343 - 1709633.1580742	433273.540458098 - 2859436.66285515
wavelet-LHH_glrlm_GrayLevelNonUniformityNormalized	wavelet-LHH	0.298714234209851 - 0.484957470359479	0.328856509819857 - 0.478704269663761
wavelet-LHH_glrlm_GrayLevelVariance	wavelet-LHH	0.286859876882908 - 1.23851307702988	0.299732346757382 - 1.24045721400415

wavelet-LHH_glrlm_HighGrayLevelRunEmphasis	wavelet-LHH	30.4592861665874 - 241.363215537172	30.5322658849385 - 307.150324682965
wavelet-LHH_glrlm_LongRunEmphasis	wavelet-LHH	3.54653887137199 - 59.6176666799836	4.22367069253702 - 49.6639990964761
wavelet-LHH_glrlm_LongRunHighGrayLevelEmphasis	wavelet-LHH	225.622719271991 - 4907.75124561658	232.524834210276 - 5550.92758297254
wavelet-LHH_glrlm_LongRunLowGrayLevelEmphasis	wavelet-LHH	0.0189539546932825 - 1.45605300891687	0.0157521627279367 - 0.898843962071509
wavelet-LHH_glrlm_LowGrayLevelRunEmphasis	wavelet-LHH	0.00422847193443456 - 0.0343537307306433	0.0033020705871851 - 0.0341250823815277
wavelet-LHH_glrlm_RunEntropy	wavelet-LHH	3.03850226669563 - 3.60174433373674	3.0481976637198 - 3.47902477811115
wavelet-LHH_glrlm_RunLengthNonUniformity	wavelet-LHH	261563.506589764 - 1348294.76868007	496244.717871888 - 2735586.51772854
wavelet-LHH_glrlm_RunLengthNonUniformityNormalized	wavelet-LHH	0.347228144454807 - 0.496324641995545	0.353094495855155 - 0.463383438987318
wavelet-LHH_glrlm_RunPercentage	wavelet-LHH	0.420113467206893 - 0.647826183384964	0.431167328233674 - 0.617967271313658
wavelet-LHH_glrlm_RunVariance	wavelet-LHH	1.08173379045686 - 51.6423474974517	1.42285964299023 - 42.3987588616596
wavelet-LHH_glrlm_ShortRunEmphasis	wavelet-LHH	0.562359872023079 - 0.72772456797519	0.576111980228083 - 0.700544584883276
wavelet-LHH_glrlm_ShortRunHighGrayLevelEmphasis	wavelet-LHH	17.2217748631174 - 165.529823499604	18.080732422268 - 196.374689378861
wavelet-LHH_glrlm_ShortRunLowGrayLevelEmphasis	wavelet-LHH	0.00290971560820589 - 0.0205104166717653	0.00211778220776974 - 0.0202370522127842
wavelet-LHH_glszm_GrayLevelNonUniformity	wavelet-LHH	2948.20674660693 - 15970.7303955934	3985.01438633298 - 20279.8142389525
wavelet-LHH_glszm_GrayLevelNonUniformityNormalized	wavelet-LHH	0.119330633175996 - 0.421005387984037	0.10699486557908 - 0.402851633293751
wavelet-LHH_glszm_GrayLevelVariance	wavelet-LHH	2.57917019664866 - 10.1889240495632	2.71088752668874 - 12.1345347559134
wavelet-LHH_glszm_HighGrayLevelZoneEmphasis	wavelet-LHH	32.7245976342835 - 253.2286760957	33.1871351351351 - 314.462327145073
wavelet-LHH_glszm_LargeAreaEmphasis	wavelet-LHH	15082913.4219218 - 2193369644.44248	26706199.5080575 - 1234291037.36086

wavelet-LHH_glszm_LargeAreaHighGrayLevelEmphasis	wavelet-LHH	2361159602.99048 - 95856683104.7421	4328426474.7632 - 138952208668.258
wavelet-LHH_glszm_LargeAreaLowGrayLevelEmphasis	wavelet-LHH	97010.0402144158 - 79018283.1446559	98384.1189223601 - 32091228.0458204
wavelet-LHH_glszm_LowGrayLevelZoneEmphasis	wavelet-LHH	0.00471466093091627 - 0.0458724687114843	0.00380997137013268 - 0.0441038007364901
wavelet-LHH_glszm_SizeZoneNonUniformity	wavelet-LHH	1288.43549874819 - 22600.3947859093	2519.22313801888 - 38367.3915294432
wavelet-LHH_glszm_SizeZoneNonUniformityNormalized	wavelet-LHH	0.152732115143732 - 0.37624683335402	0.160586961486822 - 0.367241802146981
wavelet-LHH_glszm_SmallAreaEmphasis	wavelet-LHH	0.344256679975912 - 0.634828488064436	0.358499093490062 - 0.626640968329925
wavelet-LHH_glszm_SmallAreaHighGrayLevelEmphasis	wavelet-LHH	11.9459178892729 - 158.013513729181	14.0658049428545 - 193.02789340038
wavelet-LHH_glszm_SmallAreaLowGrayLevelEmphasis	wavelet-LHH	0.00295795325114119 - 0.0201307162326608	0.00236695890950432 - 0.0189122227292877
wavelet-LHH_glszm_ZoneEntropy	wavelet-LHH	4.34281100036125 - 6.0144974239184	4.4896895960474 - 5.89095943066306
wavelet-LHH_glszm_ZonePercentage	wavelet-LHH	0.0013262381601482 - 0.0366925178245933	0.00233231973716403 - 0.0299514410407967
wavelet-LHH_glszm_ZoneVariance	wavelet-LHH	15082170.6679681 - 2192801109.76372	26705084.7912344 - 1234107204.21252
wavelet-LHH_ngtdm_Busyness	wavelet-LHH	352.37501712263 - 18818.3188069298	444.319239004145 - 14768.5303887758
wavelet-LHH_ngtdm_Coarseness	wavelet-LHH	6.02179857503385E-07 - 5.57181492093041E-06	3.50321663750644E-07 - 2.35185287540557E-06
wavelet-LHH_ngtdm_Complexity	wavelet-LHH	23.1760524268763 - 306.132218438986	30.6896852728542 - 364.714445721675
wavelet-LHH_ngtdm_Contrast	wavelet-LHH	0.000738500701910923 - 0.00340775892602552	0.000601346227584272 - 0.00263710367082537
wavelet-LHH_ngtdm_Strength	wavelet-LHH	4.93151751913945E-05 - 0.00317314148607358	7.49234268413777E-05 - 0.00362467404262789
wavelet-HLL_firstorder_Energy	wavelet-HLL	1162359732688.91 - 6763526019832.61	2124779139851.22 - 12914445786736.7
wavelet-HLL_firstorder_TotalEnergy	wavelet-HLL	1162359732688.91 - 6763526019832.61	2124779139851.22 - 12914445786736.7

wavelet-HLL_firstorder_Entropy	wavelet-HLL	1.04184589488505 - 2.53086928764886	1.07745140248964 - 2.01174361299309
wavelet-HLL_firstorder_Minimum	wavelet-HLL	-634.847271999866 - -123.790277914107	-635.395990517057 - -108.131004612059
wavelet-HLL_firstorder_10Percentile	wavelet-HLL	-37.9110896109396 - -3.22860834298715	-22.6886682658266 - -3.06509743592392
wavelet-HLL_firstorder_90Percentile	wavelet-HLL	3.30156306630704 - 38.8302213052365	3.25738679819022 - 23.3566847546401
wavelet-HLL_firstorder_Maximum	wavelet-HLL	102.05796699233 - 793.61670687803	108.53952700766 - 723.615065131258
wavelet-HLL_firstorder_Mean	wavelet-HLL	-0.0691342096601442 - 2.06147413168439E-07	-0.0416282737514999 - 1.2236693268103E-07
wavelet-HLL_firstorder_Median	wavelet-HLL	-0.0183967369103591 - 0.219603763702954	-3.73034936274053E-14 - 0.067500389343577
wavelet-HLL_firstorder_InterquartileRange	wavelet-HLL	0.884602022383575 - 29.6257056901737	1.28995993748158 - 15.6793398463613
wavelet-HLL_firstorder_Range	wavelet-HLL	225.979792449604 - 1296.73226753157	227.819421161787 - 1359.01105564832
wavelet-HLL_firstorder_MeanAbsoluteDeviation	wavelet-HLL	2.53586149564825 - 25.9072263869567	2.77865655670335 - 16.4051317570814
wavelet-HLL_firstorder_RobustMeanAbsoluteDeviation	wavelet-HLL	0.672377053882502 - 13.1723742985132	0.693386461717569 - 7.21339914547105
wavelet-HLL_firstorder_RootMeanSquared	wavelet-HLL	1000.01342266967 - 1000.86709933002	1000.01814531346 - 1000.54547924313
wavelet-HLL_firstorder_StandardDeviation	wavelet-HLL	5.18126620735135 - 43.2806441849764	6.02419755381699 - 33.4514568487346
wavelet-HLL_firstorder_Skewness	wavelet-HLL	-0.983395087464426 - 0.260837390691503	-1.15871012395352 - 0.163325679495831
wavelet-HLL_firstorder_Kurtosis	wavelet-HLL	15.8278581701916 - 143.949996767161	16.5233300568499 - 55.1692187636338
wavelet-HLL_firstorder_Variance	wavelet-HLL	26.845519511441 - 1873.21416106653	36.2909561674147 - 1118.99996530275
wavelet-HLL_firstorder_Uniformity	wavelet-HLL	0.247881496784748 - 0.507946823092382	0.340836573210816 - 0.504778143613916
wavelet-HLL_glcm_Autocorrelation	wavelet-HLL	29.3844296589518 - 702.247826210726	29.838039041317 - 702.683838231826

wavelet-HLL_glcm_ClusterProminence	wavelet-HLL	1.09313416377269 - 983.749683813981	1.19323524552711 - 740.685181586948
wavelet-HLL_glcm_ClusterShade	wavelet-HLL	-8.6333327887976 - 0.444137867224024	-4.4563513180054 - 0.442464127716588
wavelet-HLL_glcm_ClusterTendency	wavelet-HLL	0.686981083459195 - 6.95383302117059	0.695052689711931 - 4.7005761184782
wavelet-HLL_glcm_Contrast	wavelet-HLL	0.348673656381168 - 5.41122572975964	0.372485543588313 - 3.33114426311267
wavelet-HLL_glcm_Correlation	wavelet-HLL	0.110815319287225 - 0.436663181321681	0.106088290096159 - 0.376761543517704
wavelet-HLL_glcm_DifferenceAverage	wavelet-HLL	0.331780673428776 - 1.39893113209136	0.335101038522898 - 0.973259864579592
wavelet-HLL_glcm_DifferenceEntropy	wavelet-HLL	0.936947423885186 - 2.21586921502129	0.971021085293165 - 1.79039446099814
wavelet-HLL_glcm_DifferenceVariance	wavelet-HLL	0.229627465921166 - 3.36010852855062	0.240412077877505 - 2.53774737233841
wavelet-HLL_glcm_Id	wavelet-HLL	0.580919065661648 - 0.84143863749396	0.652962813337415 - 0.837602328655382
wavelet-HLL_glcm_Idm	wavelet-HLL	0.541043112776729 - 0.839204307342342	0.630948083958768 - 0.836201757256619
wavelet-HLL_glcm_Idmn	wavelet-HLL	0.996556185849788 - 0.999502594441577	0.995974273104315 - 0.999186829904793
wavelet-HLL_glcm_Idn	wavelet-HLL	0.968636941851689 - 0.98831132958282	0.964893400764801 - 0.985125533956605
wavelet-HLL_glcm_Imc1	wavelet-HLL	-0.158140959992656 - - 0.0630385598327331	-0.13385253289667 - - 0.060131887730916
wavelet-HLL_glcm_Imc2	wavelet-HLL	0.321318923273661 - 0.571893802704308	0.30599107574932 - 0.606783068848309
wavelet-HLL_glcm_InverseVariance	wavelet-HLL	0.298231365546137 - 0.499053774310593	0.31273060039183 - 0.495143380705491
wavelet-HLL_glcm_JointAverage	wavelet-HLL	5.41293382825795 - 26.4970616718474	5.45534445430663 - 26.5017259862399
wavelet-HLL_glcm_JointEnergy	wavelet-HLL	0.0780799536420461 - 0.298109971433017	0.1404235505016 - 0.297784131854818
wavelet-HLL_glcm_JointEntropy	wavelet-HLL	1.98416601819318 - 4.86632511956615	2.07352879689603 - 3.85133848460542

wavelet-HLL_glcm_MCC	wavelet-HLL	0.353918441319474 - 0.644608814546062	0.34144134801147 - 0.684514952026213
wavelet-HLL_glcm_MaximumProbability	wavelet-HLL	0.151130790099835 - 0.465938058667101	0.215859139944037 - 0.450684230930912
wavelet-HLL_glcm_SumAverage	wavelet-HLL	10.8258676565159 - 52.9941233436949	10.9106889086133 - 53.0034519724797
wavelet-HLL_glcm_SumEntropy	wavelet-HLL	1.62042455664743 - 3.04152559096527	1.66846607972524 - 2.52719255436051
wavelet-HLL_glcm_SumSquares	wavelet-HLL	0.258913684960091 - 3.09126468773256	0.270270629814665 - 2.00793009539772
wavelet-HLL_gldm_DependenceEntropy	wavelet-HLL	4.94774965664046 - 6.11304390647709	4.8911564939616 - 5.69120915876221
wavelet-HLL_gldm_DependenceNonUniformity	wavelet-HLL	87504.0433501423 - 475463.105959887	139990.563014573 - 825327.34255456
wavelet-HLL_gldm_DependenceNonUniformityNormalized	wavelet-HLL	0.0553820707760636 - 0.0909637568297702	0.0632710385832613 - 0.0866767518847981
wavelet-HLL_gldm_DependenceVariance	wavelet-HLL	16.0513241112956 - 60.7194285399008	17.1554261510026 - 38.0971735248542
wavelet-HLL_gldm_GrayLevelNonUniformity	wavelet-HLL	405797.874652427 - 3097471.23263617	723801.910216782 - 4865945.57566712
wavelet-HLL_gldm_GrayLevelVariance	wavelet-HLL	0.258998351644481 - 3.09201785833038	0.270188223026484 - 1.9887286064084
wavelet-HLL_gldm_HighGrayLevelEmphasis	wavelet-HLL	29.5540136660725 - 703.491621790127	30.0265685438711 - 704.332304672546
wavelet-HLL_gldm_LargeDependenceEmphasis	wavelet-HLL	92.3358781330479 - 369.480715963945	129.844960736559 - 362.763122544841
wavelet-HLL_gldm_LargeDependenceHighGrayLevelEmphasis	wavelet-HLL	9790.03512757331 - 149151.617822625	8910.68476195053 - 123233.535048769
wavelet-HLL_gldm_LargeDependenceLowGrayLevelEmphasis	wavelet-HLL	0.187155908919248 - 12.3177090009457	0.249896060471022 - 10.7637376227078
wavelet-HLL_gldm_LowGrayLevelEmphasis	wavelet-HLL	0.00143448274653783 - 0.0350348128311314	0.00143939140907354 - 0.0346283799834789
wavelet-HLL_gldm_SmallDependenceEmphasis	wavelet-HLL	0.00496995834284509 - 0.0700703218564783	0.00546388792054045 - 0.0404961046708104

wavelet-HLL_gldm_SmallDependenceHighGrayLevelEmphasis	wavelet-HLL	0.15066397336495 - 32.7631255381958	0.166843574506187 - 20.3973986671873
wavelet-HLL_gldm_SmallDependenceLowGrayLevelEmphasis	wavelet-HLL	2.44126845030704E-05 - 0.000214152616371517	0.000045871191690904 - 0.000225318726921124
wavelet-HLL_glrlm_GrayLevelNonUniformity	wavelet-HLL	155639.40835795 - 1356101.94787478	393406.180563238 - 2261350.37195746
wavelet-HLL_glrlm_GrayLevelNonUniformityNormalized	wavelet-HLL	0.208194845732323 - 0.484737808722781	0.29070824419346 - 0.481252194525682
wavelet-HLL_glrlm_GrayLevelVariance	wavelet-HLL	0.287392348909476 - 3.95110146442485	0.294125059085089 - 3.2168395987066
wavelet-HLL_glrlm_HighGrayLevelRunEmphasis	wavelet-HLL	30.4114776456272 - 703.70677796444	30.4571760841652 - 705.361122961012
wavelet-HLL_glrlm_LongRunEmphasis	wavelet-HLL	3.14693848322362 - 91.1144320388765	4.34418445028666 - 81.1887280619299
wavelet-HLL_glrlm_LongRunHighGrayLevelEmphasis	wavelet-HLL	513.098001946963 - 18534.9603432342	449.443836769535 - 10035.3491851201
wavelet-HLL_glrlm_LongRunLowGrayLevelEmphasis	wavelet-HLL	0.00625586168000899 - 2.32951618378527	0.00867282361974353 - 1.8760104466246
wavelet-HLL_glrlm_LowGrayLevelRunEmphasis	wavelet-HLL	0.00143943442979513 - 0.0344364664622994	0.00144988414205609 - 0.0344028056479706
wavelet-HLL_glrlm_RunEntropy	wavelet-HLL	3.48990071805862 - 3.96649444646511	3.46309135497825 - 3.92327301866022
wavelet-HLL_glrlm_RunLengthNonUniformity	wavelet-HLL	279214.747893165 - 1034049.55485107	555216.289349198 - 2996891.92441848
wavelet-HLL_glrlm_RunLengthNonUniformityNormalized	wavelet-HLL	0.257311875438347 - 0.581040056150494	0.265287205580509 - 0.484298728985154
wavelet-HLL_glrlm_RunPercentage	wavelet-HLL	0.338579458262273 - 0.711284911938032	0.342597714539944 - 0.633723866813934
wavelet-HLL_glrlm_RunVariance	wavelet-HLL	1.09771274175387 - 80.6824865192356	1.5654354285588 - 71.1890774171868
wavelet-HLL_glrlm_ShortRunEmphasis	wavelet-HLL	0.462409396043898 - 0.788055065148645	0.473348725485919 - 0.716575092599304
wavelet-HLL_glrlm_ShortRunHighGrayLevelEmphasis	wavelet-HLL	14.1343952644168 - 501.071545375252	14.4497251143528 - 454.654927899556

wavelet-HLL_glrlm_ShortRunLowGrayLevelEmphasis	wavelet-HLL	0.00102800107389265 - 0.0169156723531593	0.00094055070664277 - 0.0167513715318986
wavelet-HLL_glszm_GrayLevelNonUniformity	wavelet-HLL	1246.87151266957 - 9815.92753427172	1253.62021024076 - 18060.7125582103
wavelet-HLL_glszm_GrayLevelNonUniformityNormalized	wavelet-HLL	0.0723350356389635 - 0.319137832779516	0.0567479890289902 - 0.336919563374902
wavelet-HLL_glszm_GrayLevelVariance	wavelet-HLL	2.7732046770286 - 18.6145176533531	2.89998291670296 - 28.8777142008602
wavelet-HLL_glszm_HighGrayLevelZoneEmphasis	wavelet-HLL	31.3424622472485 - 704.579742519546	31.5789640257411 - 722.302804963027
wavelet-HLL_glszm_LargeAreaEmphasis	wavelet-HLL	6036757.46081093 - 4254919967.87817	21017001.5767832 - 3051680532.71007
wavelet-HLL_glszm_LargeAreaHighGrayLevelEmphasis	wavelet-HLL	2798005723.10685 - 435014324486.292	7294758941.63242 - 192607100657.335
wavelet-HLL_glszm_LargeAreaLowGrayLevelEmphasis	wavelet-HLL	13060.7833196811 - 153004638.923335	45023.9702007931 - 106649471.316206
wavelet-HLL_glszm_LowGrayLevelZoneEmphasis	wavelet-HLL	0.00156956262221362 - 0.0558252754959539	0.00169648314426512 - 0.0512571889496696
wavelet-HLL_glszm_SizeZoneNonUniformity	wavelet-HLL	570.136421807013 - 33049.3463400025	916.531705662937 - 66433.7558470575
wavelet-HLL_glszm_SizeZoneNonUniformityNormalized	wavelet-HLL	0.115240309539941 - 0.319283422438219	0.129424398931168 - 0.315067885200674
wavelet-HLL_glszm_SmallAreaEmphasis	wavelet-HLL	0.316843309204902 - 0.580759494310564	0.329783005983772 - 0.578530835514421
wavelet-HLL_glszm_SmallAreaHighGrayLevelEmphasis	wavelet-HLL	10.9392868424023 - 408.606864040219	11.63070139266 - 417.771724274228
wavelet-HLL_glszm_SmallAreaLowGrayLevelEmphasis	wavelet-HLL	0.000929726391488545 - 0.0217200460221432	0.00100046498469583 - 0.0199884465831304
wavelet-HLL_glszm_ZoneEntropy	wavelet-HLL	5.67121519741632 - 7.10011814865096	5.45503835529724 - 6.90147184749573
wavelet-HLL_glszm_ZonePercentage	wavelet-HLL	0.00068277935059942 - 0.0632296599277731	0.000968022332547682 - 0.0337826638111437
wavelet-HLL_glszm_ZoneVariance	wavelet-HLL	6036507.33511618 - 4252774908.87026	21016125.3586888 - 3050613373.42709
wavelet-HLL_ngtdm_Busyness	wavelet-HLL	73.117443355248 - 10551.0132477329	201.390285293822 - 12369.4629565843

wavelet-HLL_ngtdm_Coarseness	wavelet-HLL	7.83789210703772E-07 - 6.68073565598756E-06	4.25755538182139E-07 - 2.56707496401276E-06
wavelet-HLL_ngtdm_Complexity	wavelet-HLL	16.1747243598274 - 1485.34635768148	17.8860272594059 - 1045.48090836735
wavelet-HLL_ngtdm_Contrast	wavelet-HLL	0.000285179299170338 - 0.00253103464367535	0.000465634811486576 - 0.002421991751559
wavelet-HLL_ngtdm_Strength	wavelet-HLL	8.98470043836871E-05 - 0.021426256257106	7.43974085814946E-05 - 0.0107304940462501
wavelet-HLH_firstorder_Energy	wavelet-HLH	1161786465330.36 - 6763235152916.24	2124468196598.92 - 12904989405262.2
wavelet-HLH_firstorder_TotalEnergy	wavelet-HLH	1161786465330.36 - 6763235152916.24	2124468196598.92 - 12904989405262.2
wavelet-HLH_firstorder_Entropy	wavelet-HLH	1.0193820336696 - 2.30793313947554	1.05657338000718 - 1.89844329035555
wavelet-HLH_firstorder_Minimum	wavelet-HLH	-611.565293575022 - -90.9317622461552	-579.359045588179 - -111.866525698679
wavelet-HLH_firstorder_10Percentile	wavelet-HLH	-33.3303350534082 - -3.05117053518325	-21.1816032751571 - -2.95880662864929
wavelet-HLH_firstorder_90Percentile	wavelet-HLH	3.00331831409274 - 33.0041965579929	2.90473937558592 - 20.9953251046306
wavelet-HLH_firstorder_Maximum	wavelet-HLH	89.3067463442285 - 514.791474447342	117.876257385827 - 534.225653444918
wavelet-HLH_firstorder_Mean	wavelet-HLH	-0.00551945604214208 - 0.00183223701480854	-0.00307535931044648 - 0.00070116497622664
wavelet-HLH_firstorder_Median	wavelet-HLH	-0.060959204260334 - 0.0133881529721434	-0.0146179045410263 - 0.00985755741854985
wavelet-HLH_firstorder_InterquartileRange	wavelet-HLH	0.827274947807509 - 26.9715667614493	1.22902414941981 - 14.9673675244109
wavelet-HLH_firstorder_Range	wavelet-HLH	180.238508590384 - 1107.76475278543	237.029129584685 - 1082.66915575149
wavelet-HLH_firstorder_MeanAbsoluteDeviation	wavelet-HLH	2.34346752465084 - 21.5109239887767	2.41303720087068 - 14.6354563260526
wavelet-HLH_firstorder_RobustMeanAbsoluteDeviation	wavelet-HLH	0.627202839277709 - 11.7899989275658	0.651356808514083 - 6.79305431362508
wavelet-HLH_firstorder_RootMeanSquared	wavelet-HLH	1000.01059509559 - 1000.55804796003	1000.01490876893 - 1000.34767652665

wavelet-HLH_firstorder_StandardDeviation	wavelet-HLH	4.60329267307704 - 33.5774659436496	5.46056408557683 - 26.4881981535369
wavelet-HLH_firstorder_Skewness	wavelet-HLH	-0.128471986213616 - 0.435978274155505	-0.161760093004688 - 0.57262251967815
wavelet-HLH_firstorder_Kurtosis	wavelet-HLH	13.1864153255406 - 141.167061698209	15.237833593572 - 52.4281431683463
wavelet-HLH_firstorder_Variance	wavelet-HLH	21.1903034340048 - 1127.44621919695	29.8177601326915 - 701.624641421036
wavelet-HLH_firstorder_Uniformity	wavelet-HLH	0.273575595685829 - 0.511353511238005	0.35617512540063 - 0.512227564936967
wavelet-HLH_glcm_Autocorrelation	wavelet-HLH	19.441093990471 - 650.195430723788	29.1676110260832 - 600.207515489404
wavelet-HLH_glcm_ClusterProminence	wavelet-HLH	0.681121453293058 - 209.196679580624	0.723102873292441 - 120.457497620868
wavelet-HLH_glcm_ClusterShade	wavelet-HLH	-0.158292952519288 - 0.965642959179592	-0.026767127934903 - 0.384483486719113
wavelet-HLH_glcm_ClusterTendency	wavelet-HLH	0.567875041841511 - 3.68913889006605	0.553096442816034 - 2.45350992384969
wavelet-HLH_glcm_Contrast	wavelet-HLH	0.439455292146006 - 3.91811039854231	0.455845115193916 - 2.62727270424918
wavelet-HLH_glcm_Correlation	wavelet-HLH	-0.047126262128125 - 0.155975199002432	-0.0389214332348245 - 0.142636238783369
wavelet-HLH_glcm_DifferenceAverage	wavelet-HLH	0.414345259174505 - 1.25777792290917	0.4206329352695 - 0.945019495518144
wavelet-HLH_glcm_DifferenceEntropy	wavelet-HLH	0.991209850116126 - 2.10225027557923	1.02404493633198 - 1.76595097941556
wavelet-HLH_glcm_DifferenceVariance	wavelet-HLH	0.24670583824029 - 2.28915342009276	0.257863149193523 - 1.70481088116849
wavelet-HLH_glcm_Id	wavelet-HLH	0.593068994280552 - 0.802371589247206	0.656804946730049 - 0.796664709672964
wavelet-HLH_glcm_Idm	wavelet-HLH	0.556014115649083 - 0.799348869748618	0.635523869175622 - 0.79455204614933
wavelet-HLH_glcm_Idmn	wavelet-HLH	0.993261791153955 - 0.999040933868204	0.994472291530189 - 0.998961663852496
wavelet-HLH_glcm_Idn	wavelet-HLH	0.95191986704708 - 0.981886714935424	0.952438794120086 - 0.981163534005553

wavelet-HLH_glcm_Imc1	wavelet-HLH	-0.0904660763811516 - -0.0420438288072168	-0.0859018490876043 - -0.039752827331616
wavelet-HLH_glcm_Imc2	wavelet-HLH	0.229210784929859 - 0.498690901290851	0.222572330477215 - 0.499617171251171
wavelet-HLH_glcm_InverseVariance	wavelet-HLH	0.36979858279397 - 0.502394153628741	0.388756515074115 - 0.503930097565641
wavelet-HLH_glcm_JointAverage	wavelet-HLH	4.40516120636267 - 25.4997204320806	5.39719246663924 - 24.4999370112015
wavelet-HLH_glcm_JointEnergy	wavelet-HLH	0.0897752487358199 - 0.2886391688038	0.149067265155457 - 0.283443057622326
wavelet-HLH_glcm_JointEntropy	wavelet-HLH	1.98747997848375 - 4.48195524851308	2.05821170086273 - 3.65265304946257
wavelet-HLH_glcm_MCC	wavelet-HLH	0.255161024438617 - 0.534157260840687	0.244537777568255 - 0.535777659234791
wavelet-HLH_glcm_MaximumProbability	wavelet-HLH	0.162004701368309 - 0.434300142525033	0.221900379782482 - 0.414787664389428
wavelet-HLH_glcm_SumAverage	wavelet-HLH	8.81032241272534 - 50.9994408641611	10.7943849332785 - 48.9998740224029
wavelet-HLH_glcm_SumEntropy	wavelet-HLH	1.5274775028826 - 2.75461081051524	1.54248285736556 - 2.34272710042485
wavelet-HLH_glcm_SumSquares	wavelet-HLH	0.251832583496879 - 1.90181232215209	0.263450011369345 - 1.27019565702472
wavelet-HLH_gldm_DependenceEntropy	wavelet-HLH	4.56414239728354 - 5.71896645468422	4.5007784803536 - 5.28693876239626
wavelet-HLH_gldm_DependenceNonUniformity	wavelet-HLH	93881.9986049972 - 659078.781905184	168503.019233341 - 1523785.28869629
wavelet-HLH_gldm_DependenceNonUniformityNormalized	wavelet-HLH	0.0646649890792753 - 0.10663621367439	0.0793476652112828 - 0.118093596061155
wavelet-HLH_gldm_DependenceVariance	wavelet-HLH	10.8301067761241 - 56.438053906946	10.3470996840722 - 39.9817354186452
wavelet-HLH_gldm_GrayLevelNonUniformity	wavelet-HLH	447860.758975825 - 3160648.8998225	756374.921001279 - 5890342.12833266
wavelet-HLH_gldm_GrayLevelVariance	wavelet-HLH	0.251990368956664 - 1.90085656634867	0.263410997849101 - 1.259269146581
wavelet-HLH_gldm_HighGrayLevelEmphasis	wavelet-HLH	19.6684378385936 - 651.43798272633	29.3988002866031 - 601.438092506889

wavelet-HLH_gldm_LargeDependenceEmphasis	wavelet-HLH	94.6837631271594 - 309.628844461532	130.694998690905 - 298.854246694415
wavelet-HLH_gldm_LargeDependenceHighGrayLevelEmphasis	wavelet-HLH	5049.69908723917 - 90278.0876130554	5693.79637982047 - 81250.3130536578
wavelet-HLH_gldm_LargeDependenceLowGrayLevelEmphasis	wavelet-HLH	0.171967902525743 - 15.9090390959828	0.21812998156474 - 10.6692595057222
wavelet-HLH_gldm_LowGrayLevelEmphasis	wavelet-HLH	0.00154820780974322 - 0.0535908391774328	0.00167602281474841 - 0.0352480850094323
wavelet-HLH_gldm_SmallDependenceEmphasis	wavelet-HLH	0.00638098345243482 - 0.0681783609800427	0.00694127755127823 - 0.0432863100997244
wavelet-HLH_gldm_SmallDependenceHighGrayLevelEmphasis	wavelet-HLH	0.132287211948828 - 38.5766140424146	0.218895803035138 - 26.5229229592512
wavelet-HLH_gldm_SmallDependenceLowGrayLevelEmphasis	wavelet-HLH	4.95599623800613E-05 - 0.000407641870297655	5.90295712861575E-05 - 0.000323281134678331
wavelet-HLH_glrlm_GrayLevelNonUniformity	wavelet-HLH	180261.625010024 - 1615826.04878499	409383.13416952 - 2921763.57002137
wavelet-HLH_glrlm_GrayLevelNonUniformityNormalized	wavelet-HLH	0.231515420663733 - 0.491067551910653	0.304754274065203 - 0.487231122046239
wavelet-HLH_glrlm_GrayLevelVariance	wavelet-HLH	0.270136431147655 - 2.45600364220846	0.278500233311883 - 1.79183456575251
wavelet-HLH_glrlm_HighGrayLevelRunEmphasis	wavelet-HLH	20.4645544302021 - 651.998413346264	30.4941460080235 - 602.002782224737
wavelet-HLH_glrlm_LongRunEmphasis	wavelet-HLH	2.91644606347643 - 87.4834606256305	4.03494561585179 - 75.4035222010919
wavelet-HLH_glrlm_LongRunHighGrayLevelEmphasis	wavelet-HLH	235.83536557535 - 8648.37724202115	241.032119060212 - 8290.95792944833
wavelet-HLH_glrlm_LongRunLowGrayLevelEmphasis	wavelet-HLH	0.00532437507530975 - 3.66222674675054	0.00675051170633584 - 2.52638122942281
wavelet-HLH_glrlm_LowGrayLevelRunEmphasis	wavelet-HLH	0.00155297926945431 - 0.05182040739989	0.00168034757782932 - 0.0342006106104421
wavelet-HLH_glrlm_RunEntropy	wavelet-HLH	3.09557002134513 - 3.68714414400212	3.05834882972835 - 3.61052554195778

wavelet-HLH_glrlm_RunLengthNonUniformity	wavelet-HLH	271522.265859869 - 1421470.40767264	552415.332922752 - 2641756.90396276
wavelet-HLH_glrlm_RunLengthNonUniformityNormalized	wavelet-HLH	0.343887304908031 - 0.565627062287839	0.347533096698322 - 0.479126888338426
wavelet-HLH_glrlm_RunPercentage	wavelet-HLH	0.410140149909469 - 0.703196181606922	0.419019929304268 - 0.62948165911854
wavelet-HLH_glrlm_RunVariance	wavelet-HLH	0.835889671904289 - 78.9897205144475	1.34249752067875 - 67.7184008787599
wavelet-HLH_glrlm_ShortRunEmphasis	wavelet-HLH	0.55841679648429 - 0.778587981676738	0.566370105697639 - 0.713788671150344
wavelet-HLH_glrlm_ShortRunHighGrayLevelEmphasis	wavelet-HLH	11.4935184272241 - 456.252063960267	17.3603748168462 - 430.103069531363
wavelet-HLH_glrlm_ShortRunLowGrayLevelEmphasis	wavelet-HLH	0.00108913186003041 - 0.0294513292542226	0.00118575348424619 - 0.0200925738757641
wavelet-HLH_glszm_GrayLevelNonUniformity	wavelet-HLH	1852.53992642285 - 10699.9193850609	2241.45248753038 - 21805.7600409021
wavelet-HLH_glszm_GrayLevelNonUniformityNormalized	wavelet-HLH	0.103549039796944 - 0.428299478715729	0.101244884512469 - 0.397759115721898
wavelet-HLH_glszm_GrayLevelVariance	wavelet-HLH	2.51070998220195 - 13.9250340603478	2.68548564382747 - 12.9850130439926
wavelet-HLH_glszm_HighGrayLevelZoneEmphasis	wavelet-HLH	23.8849384098544 - 671.034824879468	32.8243564108973 - 618.192638251014
wavelet-HLH_glszm_LargeAreaEmphasis	wavelet-HLH	7515834.44572438 - 3620852874.92491	21290279.8144925 - 2450954828.84163
wavelet-HLH_glszm_LargeAreaHighGrayLevelEmphasis	wavelet-HLH	4149201249.7818 - 230083349308.269	9019562729.08883 - 198605128036.114
wavelet-HLH_glszm_LargeAreaLowGrayLevelEmphasis	wavelet-HLH	13641.7630099811 - 199566326.488684	35519.5027908872 - 66562340.1881999
wavelet-HLH_glszm_LowGrayLevelZoneEmphasis	wavelet-HLH	0.00165490773209908 - 0.0796412557570454	0.00178085613588135 - 0.0454650196715592
wavelet-HLH_glszm_SizeZoneNonUniformity	wavelet-HLH	836.175935944601 - 35021.2116088919	1366.25623707788 - 40044.8313290918
wavelet-HLH_glszm_SizeZoneNonUniformityNormalized	wavelet-HLH	0.149266356804881 - 0.372851669458435	0.171507160642155 - 0.370802754739603
wavelet-HLH_glszm_SmallAreaEmphasis	wavelet-HLH	0.35363886537307 - 0.631796751631322	0.381016872302832 - 0.630199668502289

wavelet-HLH_glszm_SmallAreaHighGrayLevelEmphasis	wavelet-HLH	8.95599458652582 - 420.673174236835	12.7490233969853 - 390.473060308681
wavelet-HLH_glszm_SmallAreaLowGrayLevelEmphasis	wavelet-HLH	0.00103799446639044 - 0.0332569271608798	0.00113771602260929 - 0.0193351983457189
wavelet-HLH_glszm_ZoneEntropy	wavelet-HLH	4.63867678047741 - 5.98510242014943	4.72105239379249 - 5.9592596988891
wavelet-HLH_glszm_ZonePercentage	wavelet-HLH	0.000807556534200133 - 0.0573758875645668	0.00121274426224028 - 0.0350658597365611
wavelet-HLH_glszm_ZoneVariance	wavelet-HLH	7515530.67832274 - 3619319479.62057	21289466.5514865 - 2450274903.00726
wavelet-HLH_ngtdm_Busyness	wavelet-HLH	142.456814009854 - 22107.2790812007	218.253881646922 - 16502.4861051356
wavelet-HLH_ngtdm_Coarseness	wavelet-HLH	6.33263819486871E-07 - 5.72869149048358E-06	3.43517457824128E-07 - 2.46374860224572E-06
wavelet-HLH_ngtdm_Complexity	wavelet-HLH	13.0085819013452 - 944.941746630429	20.0010014698777 - 674.441016864467
wavelet-HLH_ngtdm_Contrast	wavelet-HLH	0.000489115223346649 - 0.00379001185898554	0.000534752649082504 - 0.00317959761259173
wavelet-HLH_ngtdm_Strength	wavelet-HLH	3.65820183801032E-05 - 0.00885164543830091	5.56042231055897E-05 - 0.00821101486087896
wavelet-HHL_firstorder_Energy	wavelet-HHL	1161431838440.56 - 6762625125709.19	2123745255939.25 - 12905108065387.6
wavelet-HHL_firstorder_TotalEnergy	wavelet-HHL	1161431838440.56 - 6762625125709.19	2123745255939.25 - 12905108065387.6
wavelet-HHL_firstorder_Entropy	wavelet-HHL	0.953044964262795 - 1.65262422713456	0.962282001529877 - 1.3827783179403
wavelet-HHL_firstorder_Minimum	wavelet-HHL	-249.338464705695 - -34.3313262016229	-374.652448682453 - -36.4619560960744
wavelet-HHL_firstorder_10Percentile	wavelet-HHL	-19.628875734292 - -0.891322197307603	-13.7078455855258 - -0.906982318081287
wavelet-HHL_firstorder_90Percentile	wavelet-HHL	0.877735045122462 - 19.5900164221374	0.885382528629486 - 13.7225684250575
wavelet-HHL_firstorder_Maximum	wavelet-HHL	47.6201429365833 - 215.496078443206	48.558442610284 - 296.267513778077
wavelet-HHL_firstorder_Mean	wavelet-HHL	-2.97458588378009E-05 - 5.66398696249662E-05	-2.26588258790045E-05 - 9.63619849791438E-05

wavelet-HHL_firstorder_Median	wavelet-HHL	-0.0129304441335953 - 0.00227253039121582	-0.0123744412280866 - 1.61503897571088E-05
wavelet-HHL_firstorder_InterquartileRange	wavelet-HHL	0.352021936807722 - 17.0807334518814	0.522345244277052 - 11.8206125461218
wavelet-HHL_firstorder_Range	wavelet-HHL	85.9654940689953 - 462.036484863703	86.1813164703345 - 670.91996246053
wavelet-HHL_firstorder_MeanAbsoluteDeviation	wavelet-HHL	0.643337297218146 - 12.3952498946409	0.6820654795516 - 8.88726251032842
wavelet-HHL_firstorder_RobustMeanAbsoluteDeviation	wavelet-HHL	0.202374484844692 - 7.33535521729058	0.240804154594407 - 5.08438382499642
wavelet-HHL_firstorder_RootMeanSquared	wavelet-HHL	1000.0007533376 - 1000.16016306739	1000.00122559901 - 1000.10002024448
wavelet-HHL_firstorder_StandardDeviation	wavelet-HHL	1.22746721880272 - 17.8983487726883	1.56563070744169 - 14.1433770689767
wavelet-HHL_firstorder_Skewness	wavelet-HHL	-0.0352755210360772 - 2.06556180371997	-0.320859042459966 - 2.12751662078349
wavelet-HHL_firstorder_Kurtosis	wavelet-HHL	8.32251765405355 - 113.900264105123	8.75504485584946 - 122.259381020753
wavelet-HHL_firstorder_Variance	wavelet-HHL	1.50667577323529 - 320.350888788793	2.45119951208438 - 200.035114915256
wavelet-HHL_firstorder_Uniformity	wavelet-HHL	0.385453284199431 - 0.534273552747233	0.439704839618797 - 0.527815198156919
wavelet-HHL_glcm_Autocorrelation	wavelet-HHL	5.81849053153547 - 110.242428056944	5.79538705444598 - 240.198742688051
wavelet-HHL_glcm_ClusterProminence	wavelet-HHL	0.533810953887705 - 11.1160656116715	0.531143044640827 - 24.3085450106586
wavelet-HHL_glcm_ClusterShade	wavelet-HHL	-0.158800040391164 - 0.354999155246106	-0.158882489721359 - 0.192656785208864
wavelet-HHL_glcm_ClusterTendency	wavelet-HHL	0.52139461458333 - 1.2409237635645	0.526210882154786 - 1.067439264152
wavelet-HHL_glcm_Contrast	wavelet-HHL	0.330993988415545 - 1.27282606293271	0.360922193078106 - 1.06706512577716
wavelet-HHL_glcm_Correlation	wavelet-HHL	-0.0201185803040368 - 0.291440889893305	-0.0184910730974479 - 0.237317330909645
wavelet-HHL_glcm_DifferenceAverage	wavelet-HHL	0.330427110690153 - 0.775273816823133	0.360362674134352 - 0.639930649718712

wavelet-HHL_glcm_DifferenceEntropy	wavelet-HHL	0.91467814044859 - 1.54478994589852	0.941530192987107 - 1.31333835119789
wavelet-HHL_glcm_DifferenceVariance	wavelet-HHL	0.220797828253404 - 0.661184498939756	0.229740042239806 - 0.65180552484284
wavelet-HHL_glcm_Id	wavelet-HHL	0.674657230117406 - 0.834876874786563	0.708816163865526 - 0.819909082866372
wavelet-HHL_glcm_Idm	wavelet-HHL	0.657750111691417 - 0.834843015591297	0.700380838344035 - 0.819874522261369
wavelet-HHL_glcm_Idmn	wavelet-HHL	0.97248113770847 - 0.998312445421834	0.972511348165378 - 0.998602384971307
wavelet-HHL_glcm_Idn	wavelet-HHL	0.906463309974832 - 0.977022303567505	0.90656976167186 - 0.977694894836889
wavelet-HHL_glcm_Imc1	wavelet-HHL	-0.0680604218135581 - - 0.00992063936039838	-0.0692237457241846 - - 0.00973630750724681
wavelet-HHL_glcm_Imc2	wavelet-HHL	0.0819664149939544 - 0.338126418206627	0.0854088047724414 - 0.408484599013005
wavelet-HHL_glcm_InverseVariance	wavelet-HHL	0.329968516769669 - 0.51181493315983	0.359899202161485 - 0.511377430025728
wavelet-HHL_glcm_JointAverage	wavelet-HHL	2.40066749352133 - 10.4999610768688	2.39556418248976 - 15.4983434322221
wavelet-HHL_glcm_JointEnergy	wavelet-HHL	0.15835082749496 - 0.313754018061142	0.204194343992346 - 0.298626604809493
wavelet-HHL_glcm_JointEntropy	wavelet-HHL	1.84245183121068 - 3.2582588786521	1.87976183125363 - 2.72035863490587
wavelet-HHL_glcm_MCC	wavelet-HHL	0.0910770476945996 - 0.450683651113516	0.085725562890396 - 0.526301876169437
wavelet-HHL_glcm_MaximumProbability	wavelet-HHL	0.21559187205112 - 0.465419754333906	0.256370634028059 - 0.438076307072033
wavelet-HHL_glcm_SumAverage	wavelet-HHL	4.80133498704267 - 20.9999221537375	4.79112836497952 - 30.9966868644442
wavelet-HHL_glcm_SumEntropy	wavelet-HHL	1.50934710507956 - 2.12530577239096	1.51672797963436 - 1.84834678977316
wavelet-HHL_glcm_SumSquares	wavelet-HHL	0.233561766617572 - 0.628437456624301	0.23661140177675 - 0.53362609748229
wavelet-HHL_gldm_DependenceEntropy	wavelet-HHL	4.32173135037464 - 5.04572730771887	4.35613956990344 - 4.69285340828995

wavelet-HHL_gldm_DependenceNonUniformity	wavelet-HHL	115343.778354723 - 662123.63774081	225212.536787461 - 1434809.33156907
wavelet-HHL_gldm_DependenceNonUniformityNormalized	wavelet-HHL	0.0781363633087313 - 0.122936767817906	0.0901187473348434 - 0.12650897374172
wavelet-HHL_gldm_DependenceVariance	wavelet-HHL	6.10035782219585 - 41.1559028071832	7.07038817757541 - 32.4403102136022
wavelet-HHL_gldm_GrayLevelNonUniformity	wavelet-HHL	570222.269738428 - 3381562.16451099	933758.956233836 - 5787456.29657635
wavelet-HHL_gldm_GrayLevelVariance	wavelet-HHL	0.23334256978821 - 0.624707182923945	0.23653520320606 - 0.530526111439625
wavelet-HHL_gldm_HighGrayLevelEmphasis	wavelet-HHL	6.00456747404844 - 110.686602116025	5.97935968683374 - 240.722228865093
wavelet-HHL_gldm_LargeDependenceEmphasis	wavelet-HHL	133.48109908959 - 347.377731641676	155.864471907192 - 324.585210704913
wavelet-HHL_gldm_LargeDependenceHighGrayLevelEmphasis	wavelet-HHL	1388.96390000501 - 29498.1496881049	1381.75630827608 - 41857.7531964508
wavelet-HHL_gldm_LargeDependenceLowGrayLevelEmphasis	wavelet-HHL	1.42465106343223 - 65.9021577427971	0.727385798002702 - 67.2863756519928
wavelet-HHL_gldm_LowGrayLevelEmphasis	wavelet-HHL	0.00917753236033278 - 0.194347825865343	0.00419403785045296 - 0.195032505320409
wavelet-HHL_gldm_SmallDependenceEmphasis	wavelet-HHL	0.00461582200996557 - 0.0366435223672232	0.00477240353664871 - 0.0229680309327125
wavelet-HHL_gldm_SmallDependenceHighGrayLevelEmphasis	wavelet-HHL	0.031075041686861 - 3.42255517535243	0.0306496879402967 - 3.7656715741899
wavelet-HHL_gldm_SmallDependenceLowGrayLevelEmphasis	wavelet-HHL	0.000102821747165754 - 0.00107886128667057	0.000070297128654751 - 0.00104470928018623
wavelet-HHL_glrlm_GrayLevelNonUniformity	wavelet-HHL	219752.633842206 - 1633670.71274844	498951.969073019 - 2854112.40673937
wavelet-HHL_glrlm_GrayLevelNonUniformityNormalized	wavelet-HHL	0.342368538670698 - 0.500071347409003	0.407166069462303 - 0.49997883844345
wavelet-HHL_glrlm_GrayLevelVariance	wavelet-HHL	0.250057375838991 - 0.806135930515612	0.250310764235144 - 0.728197827394629

wavelet-HHL_glrlm_HighGrayLevelRunEmphasis	wavelet-HHL	6.46174038839295 - 110.8212224015	6.45944765814412 - 240.915384680999
wavelet-HHL_glrlm_LongRunEmphasis	wavelet-HHL	3.83632188963142 - 57.6774391315433	4.68998112460465 - 47.8234885280244
wavelet-HHL_glrlm_LongRunHighGrayLevelEmphasis	wavelet-HHL	45.6156565264647 - 2450.28066171529	46.5210484359736 - 1456.53755135981
wavelet-HHL_glrlm_LongRunLowGrayLevelEmphasis	wavelet-HHL	0.0431742640196439 - 9.18723370691857	0.0228418286704672 - 9.83220986346852
wavelet-HHL_glrlm_LowGrayLevelRunEmphasis	wavelet-HHL	0.00918522897314082 - 0.181690858860296	0.00420677116013956 - 0.181835664006207
wavelet-HHL_glrlm_RunEntropy	wavelet-HHL	2.95890868785375 - 3.35278679072785	3.02016083934946 - 3.34643876035909
wavelet-HHL_glrlm_RunLengthNonUniformity	wavelet-HHL	193367.537192796 - 1065877.37846661	438019.254829506 - 2642831.14432533
wavelet-HHL_glrlm_RunLengthNonUniformityNormalized	wavelet-HHL	0.285954594570542 - 0.46027543205639	0.304135449195014 - 0.408799643212614
wavelet-HHL_glrlm_RunPercentage	wavelet-HHL	0.357543367606805 - 0.619965518006157	0.381067430972651 - 0.575745579394565
wavelet-HHL_glrlm_RunVariance	wavelet-HHL	1.15706633444311 - 49.5556541436635	1.4820559992619 - 40.6746486868253
wavelet-HHL_glrlm_ShortRunEmphasis	wavelet-HHL	0.513126457578343 - 0.698536604792002	0.529969789911959 - 0.650865364035602
wavelet-HHL_glrlm_ShortRunHighGrayLevelEmphasis	wavelet-HHL	3.44407397934777 - 72.2083427565158	3.46639804529255 - 150.028803172963
wavelet-HHL_glrlm_ShortRunLowGrayLevelEmphasis	wavelet-HHL	0.00554910549878815 - 0.100911504724742	0.00262738175400277 - 0.10113038302603
wavelet-HHL_glszm_GrayLevelNonUniformity	wavelet-HHL	27.6111111111111 - 12020.1568956125	147.199203187251 - 17738.5983866815
wavelet-HHL_glszm_GrayLevelNonUniformityNormalized	wavelet-HHL	0.210605937333781 - 0.635979902234447	0.121129435140893 - 0.671833590443416
wavelet-HHL_glszm_GrayLevelVariance	wavelet-HHL	0.996039944903581 - 5.48269497556682	0.818272725829749 - 9.93511527443458
wavelet-HHL_glszm_HighGrayLevelZoneEmphasis	wavelet-HHL	10.2552083333333 - 116.669863315697	12.1728624535316 - 246.26321217872
wavelet-HHL_glszm_LargeAreaEmphasis	wavelet-HHL	21347860.5180824 - 127849191869.288	54157398.6331341 - 68039504165.0438

wavelet-HHL_glszm_LargeAreaHighGrayLevelEmphasis	wavelet-HHL	1932625141.57725 - 1444873517349.95	3924586441.21054 - 613076687411.92
wavelet-HHL_glszm_LargeAreaLowGrayLevelEmphasis	wavelet-HHL	238490.72492284 - 20979182437.9559	446280.691216202 - 14174709473.8783
wavelet-HHL_glszm_LowGrayLevelZoneEmphasis	wavelet-HHL	0.0103112070114933 - 0.376608222490931	0.00492771430300457 - 0.301966129698472
wavelet-HHL_glszm_SizeZoneNonUniformity	wavelet-HHL	22.1944444444444 - 18458.4175920882	80.1474103585657 - 27836.9141851884
wavelet-HHL_glszm_SizeZoneNonUniformityNormalized	wavelet-HHL	0.17791551882461 - 0.500993018160971	0.293805814375073 - 0.52337890625
wavelet-HHL_glszm_SmallAreaEmphasis	wavelet-HHL	0.378322972623641 - 0.734723130373408	0.555364521507617 - 0.750875122534635
wavelet-HHL_glszm_SmallAreaHighGrayLevelEmphasis	wavelet-HHL	5.88106798079348 - 73.9341680146996	7.6369785014807 - 146.399651930712
wavelet-HHL_glszm_SmallAreaLowGrayLevelEmphasis	wavelet-HHL	0.00604606314893061 - 0.263346314625246	0.00296268051370766 - 0.217209359432272
wavelet-HHL_glszm_ZoneEntropy	wavelet-HHL	2.79522366908709 - 5.27882919926148	2.40101229139254 - 5.77443554987759
wavelet-HHL_glszm_ZonePercentage	wavelet-HHL	1.84544405997693E-05 - 0.0291534112288829	4.38642480164971E-05 - 0.0171792857802114
wavelet-HHL_glszm_ZoneVariance	wavelet-HHL	21346683.9386762 - 125969969369.288	54154010.2712212 - 67519773163.3137
wavelet-HHL_ngtdm_Busyness	wavelet-HHL	585.786249875363 - 145067.759677403	1194.17137279541 - 127928.515183419
wavelet-HHL_ngtdm_Coarseness	wavelet-HHL	6.32910114602784E-07 - 4.83299413667839E-06	3.52097089206715E-07 - 2.07494115220934E-06
wavelet-HHL_ngtdm_Complexity	wavelet-HHL	3.27139327487986 - 105.082803908406	3.267148187474 - 214.309880823299
wavelet-HHL_ngtdm_Contrast	wavelet-HHL	0.000900468147824599 - 0.0194838458764643	0.000872293352225107 - 0.0194603683555847
wavelet-HHL_ngtdm_Strength	wavelet-HHL	3.79663163630114E-06 - 0.00230462093896388	4.29754212577215E-06 - 0.00113008604518203
wavelet-HHH_firstorder_Energy	wavelet-HHH	1161406124824.68 - 6762622738556.85	2123734500857.92 - 12903801709679.1
wavelet-HHH_firstorder_TotalEnergy	wavelet-HHH	1161406124824.68 - 6762622738556.85	2123734500857.92 - 12903801709679.1

wavelet-HHH_firstorder_Entropy	wavelet-HHH	0.95050043516414 - 1.60498831197605	0.962324348211965 - 1.35983820458483
wavelet-HHH_firstorder_Minimum	wavelet-HHH	-236.70662401541 - -38.8510436061392	-252.307066757299 - -53.1565033115802
wavelet-HHH_firstorder_10Percentile	wavelet-HHH	-18.9710385301605 - -0.850524792173821	-13.3796591844888 - -0.858377963642662
wavelet-HHH_firstorder_90Percentile	wavelet-HHH	0.856892792879725 - 18.9496383400634	0.867053196509109 - 13.3757576414287
wavelet-HHH_firstorder_Maximum	wavelet-HHH	28.9867919478364 - 254.926548389824	36.9316500543924 - 276.493492060381
wavelet-HHH_firstorder_Mean	wavelet-HHH	-2.34056080153747E-06 - 1.39707884198429E-05	-2.90081108605849E-06 - 4.41682569774659E-06
wavelet-HHH_firstorder_Median	wavelet-HHH	-0.0129520002244166 - 0.00866801389863592	-0.00075725457006879 - 0.00553399712082267
wavelet-HHH_firstorder_InterquartileRange	wavelet-HHH	0.342106595164899 - 16.6397173883581	0.514516357273503 - 11.6013479840735
wavelet-HHH_firstorder_Range	wavelet-HHH	67.8378355539756 - 491.633172405234	90.0881533659726 - 526.469260928292
wavelet-HHH_firstorder_MeanAbsoluteDeviation	wavelet-HHH	0.611384458852693 - 11.8946817548928	0.65624452631129 - 8.6527733207732
wavelet-HHH_firstorder_RobustMeanAbsoluteDeviation	wavelet-HHH	0.195603677476549 - 7.13047631730516	0.234191544031904 - 4.98400840057501
wavelet-HHH_firstorder_RootMeanSquared	wavelet-HHH	1000.00073046261 - 1000.14246076887	1000.00118238595 - 1000.08155121121
wavelet-HHH_firstorder_StandardDeviation	wavelet-HHH	1.20868761839132 - 16.8802515002828	1.53778193963472 - 12.7716433809455
wavelet-HHH_firstorder_Skewness	wavelet-HHH	-1.25142678659144 - 0.12138030122	-1.96490126181898 - 0.125567228754738
wavelet-HHH_firstorder_Kurtosis	wavelet-HHH	6.78472806995817 - 184.566192918083	9.03651203904277 - 128.543255032309
wavelet-HHH_firstorder_Variance	wavelet-HHH	1.46092575885247 - 284.9428907128	2.36477329386672 - 163.114874650049
wavelet-HHH_firstorder_Uniformity	wavelet-HHH	0.393187025645457 - 0.535568267620623	0.443630390731783 - 0.527677109792114
wavelet-HHH_glcm_Autocorrelation	wavelet-HHH	6.01482016039353 - 107.888418865707	11.4768138118163 - 132.241273763976

wavelet-HHH_glcm_ClusterProminence	wavelet-HHH	0.493330512632088 - 6.59120925961544	0.49442879151193 - 5.85314577427661
wavelet-HHH_glcm_ClusterShade	wavelet-HHH	-0.140576681346507 - 0.293316586409688	-0.139214720013553 - 0.165892111727576
wavelet-HHH_glcm_ClusterTendency	wavelet-HHH	0.492275522843617 - 1.10526046171008	0.4914344589346 - 0.779810825473608
wavelet-HHH_glcm_Contrast	wavelet-HHH	0.379516613007059 - 1.17918606906922	0.39568898017628 - 0.830803279352988
wavelet-HHH_glcm_Correlation	wavelet-HHH	-0.0324754829953693 - 0.184519218744739	-0.031693311309195 - 0.163581486363381
wavelet-HHH_glcm_DifferenceAverage	wavelet-HHH	0.379044952164689 - 0.756673116321564	0.395170995762345 - 0.633990676198645
wavelet-HHH_glcm_DifferenceEntropy	wavelet-HHH	0.953479251142843 - 1.51213814071012	0.965956035863647 - 1.29843998883853
wavelet-HHH_glcm_DifferenceVariance	wavelet-HHH	0.233754858577252 - 0.598181608031695	0.237876645225546 - 0.42523257668844
wavelet-HHH_glcm_Id	wavelet-HHH	0.677289704451067 - 0.810552756871876	0.709672546473711 - 0.802498314680151
wavelet-HHH_glcm_Idm	wavelet-HHH	0.661418701410803 - 0.810524499627983	0.70172544746274 - 0.802466218074638
wavelet-HHH_glcm_Idmn	wavelet-HHH	0.972868317046055 - 0.998710807917193	0.980424499353106 - 0.99841162487129
wavelet-HHH_glcm_Idn	wavelet-HHH	0.907756019259551 - 0.97942672236235	0.91523831152415 - 0.974630274869657
wavelet-HHH_glcm_Imc1	wavelet-HHH	-0.0423584457137325 - - 0.00697695188087712	-0.0462787257217955 - - 0.00784235845605438
wavelet-HHH_glcm_Imc2	wavelet-HHH	0.0729041395216252 - 0.30544832094368	0.0743733930465397 - 0.322910620067905
wavelet-HHH_glcm_InverseVariance	wavelet-HHH	0.37866280545742 - 0.514540076217121	0.39474096253221 - 0.5140327425423
wavelet-HHH_glcm_JointAverage	wavelet-HHH	2.44908451391429 - 10.385229990359	3.38202834160615 - 11.5000848351512
wavelet-HHH_glcm_JointEnergy	wavelet-HHH	0.163705327479955 - 0.301959671807797	0.207548700459037 - 0.290255891701509
wavelet-HHH_glcm_JointEntropy	wavelet-HHH	1.87051330560132 - 3.16808481189473	1.90029154571184 - 2.67311717314076

wavelet-HHH_glcm_MCC	wavelet-HHH	0.100153028687274 - 0.426979786611514	0.0951880707211347 - 0.424613584045765
wavelet-HHH_glcm_MaximumProbability	wavelet-HHH	0.219466376184533 - 0.443507042454825	0.258023035915093 - 0.420302426813015
wavelet-HHH_glcm_SumAverage	wavelet-HHH	4.89816902782858 - 20.770459980718	6.7640566832123 - 23.0001696703024
wavelet-HHH_glcm_SumEntropy	wavelet-HHH	1.48527060302039 - 2.06470504404429	1.4853834018223 - 1.81420951441377
wavelet-HHH_glcm_SumSquares	wavelet-HHH	0.232703892029155 - 0.571111632694825	0.236542455761523 - 0.402653526206649
wavelet-HHH_gldm_DependenceEntropy	wavelet-HHH	4.00985555600644 - 4.87768030433981	4.07935672546342 - 4.49050679409552
wavelet-HHH_gldm_DependenceNonUniformity	wavelet-HHH	130110.104552876 - 927750.491446189	255817.490799603 - 2001761.37259455
wavelet-HHH_gldm_DependenceNonUniformityNormalized	wavelet-HHH	0.0915748162905845 - 0.155450138726774	0.113802428015977 - 0.156229446013038
wavelet-HHH_gldm_DependenceVariance	wavelet-HHH	4.33696516111968 - 39.5938434078489	5.24960104981393 - 34.5972829137868
wavelet-HHH_gldm_GrayLevelNonUniformity	wavelet-HHH	578762.041679583 - 3382780.31499068	942095.272279578 - 6268696.69372667
wavelet-HHH_gldm_GrayLevelVariance	wavelet-HHH	0.23249236300642 - 0.568989349752173	0.236455196177743 - 0.402417251998323
wavelet-HHH_gldm_HighGrayLevelEmphasis	wavelet-HHH	6.24134666154043 - 108.146991960651	11.6721998228106 - 132.624919055394
wavelet-HHH_gldm_LargeDependenceEmphasis	wavelet-HHH	133.339119301383 - 309.690373787859	155.073625779571 - 297.906290557144
wavelet-HHH_gldm_LargeDependenceHighGrayLevelEmphasis	wavelet-HHH	1299.2743842112 - 29196.2593465187	2266.2578590652 - 23064.4487361078
wavelet-HHH_gldm_LargeDependenceLowGrayLevelEmphasis	wavelet-HHH	1.49184524000975 - 45.405364994945	1.32384006485993 - 29.7902393733737
wavelet-HHH_gldm_LowGrayLevelEmphasis	wavelet-HHH	0.00936025236552445 - 0.187747013257009	0.00763021275136167 - 0.0925766568785324
wavelet-HHH_gldm_SmallDependenceEmphasis	wavelet-HHH	0.00529647857995773 - 0.0372748058151054	0.00530077208873438 - 0.023655963494991

wavelet-HHH_gldm_SmallDependenceHighGrayLevelEmphasis	wavelet-HHH	0.0392137183052975 - 3.48204975744544	0.0646904086839768 - 2.43848460139633
wavelet-HHH_gldm_SmallDependenceLowGrayLevelEmphasis	wavelet-HHH	0.000142643533505174 - 0.00111334054631476	0.0001070179898379 - 0.000575594264512805
wavelet-HHH_glrlm_GrayLevelNonUniformity	wavelet-HHH	228055.605078859 - 1755818.88759187	504831.961368713 - 3134500.06668762
wavelet-HHH_glrlm_GrayLevelNonUniformityNormalized	wavelet-HHH	0.351078709369016 - 0.500080336109801	0.412179625172997 - 0.499929990431349
wavelet-HHH_glrlm_GrayLevelVariance	wavelet-HHH	0.249992392026395 - 0.728353181961124	0.250198951947633 - 0.499071548851631
wavelet-HHH_glrlm_HighGrayLevelRunEmphasis	wavelet-HHH	6.46707631808537 - 110.369358127636	12.4286079565802 - 132.714201720462
wavelet-HHH_glrlm_LongRunEmphasis	wavelet-HHH	3.81270239995092 - 54.5017889007784	4.64418722922882 - 45.50965373626
wavelet-HHH_glrlm_LongRunHighGrayLevelEmphasis	wavelet-HHH	72.9799109897132 - 2426.65238468983	74.9629168114278 - 1323.70359785552
wavelet-HHH_glrlm_LongRunLowGrayLevelEmphasis	wavelet-HHH	0.0428567060491023 - 5.92715428794854	0.041008704550724 - 4.92935310566407
wavelet-HHH_glrlm_LowGrayLevelRunEmphasis	wavelet-HHH	0.00921424128384194 - 0.181483607556534	0.00764885338967752 - 0.0873539037678068
wavelet-HHH_glrlm_RunEntropy	wavelet-HHH	2.88648869233371 - 3.2664820282413	2.88623316484046 - 3.18405654283097
wavelet-HHH_glrlm_RunLengthNonUniformity	wavelet-HHH	193551.686644186 - 1259413.55172428	440916.692872916 - 2396145.75162694
wavelet-HHH_glrlm_RunLengthNonUniformityNormalized	wavelet-HHH	0.33967589163425 - 0.456936172489882	0.34873062862489 - 0.407484271619865
wavelet-HHH_glrlm_RunPercentage	wavelet-HHH	0.403463395078159 - 0.618390606598154	0.414353882414662 - 0.575489266215503
wavelet-HHH_glrlm_RunVariance	wavelet-HHH	1.14635580393831 - 47.9552195528574	1.47467736629925 - 39.4908065613464
wavelet-HHH_glrlm_ShortRunEmphasis	wavelet-HHH	0.57555057349239 - 0.696895035618994	0.581893757711809 - 0.651526196415538
wavelet-HHH_glrlm_ShortRunHighGrayLevelEmphasis	wavelet-HHH	3.83183020964454 - 68.5048395607224	7.29149365819221 - 82.0092181799623

wavelet-HHH_glrlm_ShortRunLowGrayLevelEmphasis	wavelet-HHH	0.00570175869595512 - 0.106165917806126	0.00473699289308302 - 0.051942929308988
wavelet-HHH_glszm_GrayLevelNonUniformity	wavelet-HHH	19.35 - 11471.5301993723	131.73786407767 - 19190.6704289822
wavelet-HHH_glszm_GrayLevelNonUniformityNormalized	wavelet-HHH	0.259015335622216 - 0.65539737654321	0.178107857226605 - 0.6469140625
wavelet-HHH_glszm_GrayLevelVariance	wavelet-HHH	0.909923384512072 - 4.14239603714325	1.37459851436518 - 6.36261327131392
wavelet-HHH_glszm_HighGrayLevelZoneEmphasis	wavelet-HHH	4.375 - 113.908040071924	7.93215339233038 - 139.856565090297
wavelet-HHH_glszm_LargeAreaEmphasis	wavelet-HHH	23544694.4033507 - 192207661425.7	57335250.7943772 - 86936597483.301
wavelet-HHH_glszm_LargeAreaHighGrayLevelEmphasis	wavelet-HHH	2130181040.22956 - 1721974512276.98	3239786478.80634 - 1067984199064.98
wavelet-HHH_glszm_LargeAreaLowGrayLevelEmphasis	wavelet-HHH	263228.28220248 - 37444430994.8424	603112.64778189 - 7676602347.72996
wavelet-HHH_glszm_LowGrayLevelZoneEmphasis	wavelet-HHH	0.0102522266883353 - 0.6875	0.00885072572978668 - 0.238448049819731
wavelet-HHH_glszm_SizeZoneNonUniformity	wavelet-HHH	13.8 - 21682.6605026079	98.8640776699029 - 25521.832495452
wavelet-HHH_glszm_SizeZoneNonUniformityNormalized	wavelet-HHH	0.22030612244898 - 0.549515235457064	0.310808060248321 - 0.55876472278081
wavelet-HHH_glszm_SmallAreaEmphasis	wavelet-HHH	0.461526490494601 - 0.76559190559975	0.572704488504694 - 0.775671541664895
wavelet-HHH_glszm_SmallAreaHighGrayLevelEmphasis	wavelet-HHH	2.37789294303475 - 87.0709507115808	4.87036493819745 - 96.9905743590581
wavelet-HHH_glszm_SmallAreaLowGrayLevelEmphasis	wavelet-HHH	0.00778290221460223 - 0.450695866237741	0.00613065356759801 - 0.165856193134756
wavelet-HHH_glszm_ZoneEntropy	wavelet-HHH	2.4676970009058 - 4.23616510986049	2.36329337085137 - 4.79844997621937
wavelet-HHH_glszm_ZonePercentage	wavelet-HHH	1.02524669998718E-05 - 0.0270539209218454	3.44349163365261E-05 - 0.0163811143697224
wavelet-HHH_glszm_ZoneVariance	wavelet-HHH	23543328.1238024 - 182694097519.45	57331524.1914993 - 86093258966.7113
wavelet-HHH_ngtdm_Busyness	wavelet-HHH	544.388739327686 - 87036.8777050745	1401.87199864364 - 69310.2735979322
wavelet-HHH_ngtdm_Coarseness	wavelet-HHH	5.86686560843851E-07 - 4.64311842456309E-06	3.21656490097281E-07 - 2.04570434535446E-06

wavelet-HHH_ngtdm_Complexity	wavelet-HHH	3.20939883679547 - 94.9261766742056	4.87998322605792 - 134.224547608235
wavelet-HHH_ngtdm_Contrast	wavelet-HHH	0.000668454393358334 - 0.0189010628934133	0.000919503417887516 - 0.0127046184000963
wavelet-HHH_ngtdm_Strength	wavelet-HHH	6.70844040815273E-06 - 0.00312457690441029	8.23800399588088E-06 - 0.00119818070843586
wavelet-LLL_firstorder_Energy	wavelet-LLL	1256771330295.63 - 7307559781166.93	2250158746196.7 - 14016731703099.4
wavelet-LLL_firstorder_TotalEnergy	wavelet-LLL	1256771330295.63 - 7307559781166.93	2250158746196.7 - 14016731703099.4
wavelet-LLL_firstorder_Entropy	wavelet-LLL	2.95745628820842 - 5.07275257185012	2.9243370028248 - 4.8552338790929
wavelet-LLL_firstorder_Minimum	wavelet-LLL	-552.250139205897 - -228.744593412175	-397.412817980195 - -214.894926038388
wavelet-LLL_firstorder_10Percentile	wavelet-LLL	-389.354859649966 - -150.980192727135	-266.071782866187 - -138.019901631305
wavelet-LLL_firstorder_90Percentile	wavelet-LLL	303.383865693661 - 475.655244990006	335.607834156117 - 505.615765042728
wavelet-LLL_firstorder_Maximum	wavelet-LLL	797.413529723026 - 1267.62771907007	750.186652850021 - 1323.91815650287
wavelet-LLL_firstorder_Mean	wavelet-LLL	-5.00789735216881 - 10.8331169001966	-2.54983492225044 - 12.0133448701851
wavelet-LLL_firstorder_Median	wavelet-LLL	-199.312853703264 - 23.7225208633709	-206.068060356111 - -115.536644411944
wavelet-LLL_firstorder_InterquartileRange	wavelet-LLL	192.639511848366 - 528.37626260723	67.6408723229535 - 527.689768239102
wavelet-LLL_firstorder_Range	wavelet-LLL	1100.65083864329 - 1735.14776598135	1037.49048631421 - 1672.95020694124
wavelet-LLL_firstorder_MeanAbsoluteDeviation	wavelet-LLL	203.888648152838 - 253.434893710991	194.850028225335 - 257.375678748287
wavelet-LLL_firstorder_RobustMeanAbsoluteDeviation	wavelet-LLL	125.425059754621 - 217.595611467686	107.146623793739 - 223.08422040618
wavelet-LLL_firstorder_RootMeanSquared	wavelet-LLL	1028.81130278415 - 1047.82668921166	1029.36598187746 - 1050.27867648807
wavelet-LLL_firstorder_StandardDeviation	wavelet-LLL	255.134021159417 - 279.480430677641	254.337360277474 - 283.297637059527

wavelet-LLL_firstorder_Skewness	wavelet-LLL	0.387621357396454 - 1.85492154970004	0.533705888778856 - 2.12108521810084
wavelet-LLL_firstorder_Kurtosis	wavelet-LLL	1.709453772202 - 6.02187677104684	1.71791076031969 - 7.03521803639768
wavelet-LLL_firstorder_Variance	wavelet-LLL	65093.368752974 - 78109.3111317598	64687.4928329136 - 80257.5511635114
wavelet-LLL_firstorder_Uniformity	wavelet-LLL	0.0394006909026285 - 0.365390821944224	0.0517458082279103 - 0.368996059683358
wavelet-LLL_glcm_Autocorrelation	wavelet-LLL	236.111680326664 - 652.649251856932	216.02693475614 - 389.979475536607
wavelet-LLL_glcm_ClusterProminence	wavelet-LLL	363663.709770461 - 1245940.71927595	355502.753173749 - 1569623.91478333
wavelet-LLL_glcm_ClusterShade	wavelet-LLL	2800.29759205338 - 19289.6838251846	4922.22493367277 - 21894.5892155493
wavelet-LLL_glcm_ClusterTendency	wavelet-LLL	388.145290092495 - 488.495232783405	387.681649700136 - 519.164204889671
wavelet-LLL_glcm_Contrast	wavelet-LLL	10.3709303090029 - 21.4382663200014	9.47977885309289 - 23.0988862326203
wavelet-LLL_glcm_Correlation	wavelet-LLL	0.899871100411928 - 0.957559161913145	0.892067138673346 - 0.962709131681972
wavelet-LLL_glcm_DifferenceAverage	wavelet-LLL	1.40827067139967 - 2.62309334893654	1.17709199831523 - 2.54839508868587
wavelet-LLL_glcm_DifferenceEntropy	wavelet-LLL	1.88531518997916 - 2.980927558027	1.88877582157208 - 2.87855956079403
wavelet-LLL_glcm_DifferenceVariance	wavelet-LLL	7.88366371399221 - 16.0224012898169	7.01317189801763 - 15.9260018949415
wavelet-LLL_glcm_Id	wavelet-LLL	0.51780420838098 - 0.790489028579573	0.585188581043126 - 0.781790443731636
wavelet-LLL_glcm_Idm	wavelet-LLL	0.461928146411685 - 0.767369292762796	0.541044086222051 - 0.76211796839157
wavelet-LLL_glcm_Idmn	wavelet-LLL	0.993423417102041 - 0.996863923218534	0.994190593418394 - 0.997084512862772
wavelet-LLL_glcm_Idn	wavelet-LLL	0.960353661793718 - 0.97910809157515	0.964364197094341 - 0.981928199393063
wavelet-LLL_glcm_Imc1	wavelet-LLL	-0.508427243324375 - -0.344692661504645	-0.504819786668385 - -0.377342097586339

wavelet-LLL_glcm_Imc2	wavelet-LLL	0.965085663023519 - 0.990392991061974	0.960640644897213 - 0.990591262403381
wavelet-LLL_glcm_InverseVariance	wavelet-LLL	0.109376655674111 - 0.338681249089435	0.120954221423479 - 0.263765257844761
wavelet-LLL_glcm_JointAverage	wavelet-LLL	10.8679053410399 - 23.6778378818859	9.68176027903978 - 16.9276788951609
wavelet-LLL_glcm_JointEnergy	wavelet-LLL	0.00971833829849194 - 0.339725973437255	0.0237748051665151 - 0.323031786787748
wavelet-LLL_glcm_JointEntropy	wavelet-LLL	4.51854941641402 - 8.37101858792247	4.55872183237041 - 7.80947267056015
wavelet-LLL_glcm_MCC	wavelet-LLL	0.943316194985112 - 0.990077739812981	0.938130768854547 - 0.990377299778626
wavelet-LLL_glcm_MaximumProbability	wavelet-LLL	0.0449511798895542 - 0.57629251186861	0.075007914108032 - 0.562294445873972
wavelet-LLL_glcm_SumAverage	wavelet-LLL	21.7358106820798 - 47.3556757637718	19.3635205580796 - 33.8553577903218
wavelet-LLL_glcm_SumEntropy	wavelet-LLL	3.41874767844062 - 6.00497466288251	3.50537920362331 - 5.72626132008003
wavelet-LLL_glcm_SumSquares	wavelet-LLL	102.065279341813 - 125.717717846827	101.977467712716 - 132.724272696634
wavelet-LLL_gldm_DependenceEntropy	wavelet-LLL	4.96456646195908 - 8.82459102232962	5.23155463668902 - 8.62220321320214
wavelet-LLL_gldm_DependenceNonUniformity	wavelet-LLL	99430.0768947506 - 1311904.20838173	105549.234600236 - 2561225.43296485
wavelet-LLL_gldm_DependenceNonUniformityNormalized	wavelet-LLL	0.0468213833156985 - 0.320205766292925	0.0497028799155757 - 0.276606844876003
wavelet-LLL_gldm_DependenceVariance	wavelet-LLL	39.581344974043 - 110.586535538004	79.1315626433896 - 110.334427633975
wavelet-LLL_gldm_GrayLevelNonUniformity	wavelet-LLL	64501.4526518206 - 1831767.79255771	109887.605336023 - 3860452.27777513
wavelet-LLL_gldm_GrayLevelVariance	wavelet-LLL	104.241599482366 - 124.184291481692	103.582411460536 - 130.273941548457
wavelet-LLL_gldm_HighGrayLevelEmphasis	wavelet-LLL	240.523912156158 - 661.588264722699	219.027528985058 - 393.908182033938
wavelet-LLL_gldm_LargeDependenceEmphasis	wavelet-LLL	105.017251616308 - 469.071642693285	204.899717649807 - 453.524143307546

wavelet-LLL_gldm_LargeDependenceHighGrayLevelEmphasis	wavelet-LLL	17278.9446044257 - 44088.4513821172	11351.7624886333 - 31605.1868176575
wavelet-LLL_gldm_LargeDependenceLowGrayLevelEmphasis	wavelet-LLL	0.57598568034328 - 29.8374286274831	3.08561829904218 - 48.1173583566114
wavelet-LLL_gldm_LowGrayLevelEmphasis	wavelet-LLL	0.00391724773355178 - 0.0519134862136608	0.0109875875473109 - 0.0836590589434066
wavelet-LLL_gldm_SmallDependenceEmphasis	wavelet-LLL	0.0511793340440442 - 0.122807164487664	0.0493781988228837 - 0.118971824614798
wavelet-LLL_gldm_SmallDependenceHighGrayLevelEmphasis	wavelet-LLL	30.3405192345865 - 130.238756507729	25.079817825223 - 85.4704626029467
wavelet-LLL_gldm_SmallDependenceLowGrayLevelEmphasis	wavelet-LLL	0.000207661375903361 - 0.000507188596733602	0.00023779745779564 - 0.000530869974774124
wavelet-LLL_glrlm_GrayLevelNonUniformity	wavelet-LLL	22128.7541228542 - 159329.951109185	38007.2147095341 - 159851.072766624
wavelet-LLL_glrlm_GrayLevelNonUniformityNormalized	wavelet-LLL	0.0301182442766455 - 0.0557765770905129	0.0308424963053839 - 0.0578903891184892
wavelet-LLL_glrlm_GrayLevelVariance	wavelet-LLL	90.8968491244502 - 168.642736912731	88.932056744141 - 197.221735120366
wavelet-LLL_glrlm_HighGrayLevelRunEmphasis	wavelet-LLL	450.056572020364 - 756.346514738561	427.453717668251 - 664.158782986979
wavelet-LLL_glrlm_LongRunEmphasis	wavelet-LLL	3.65049874749107 - 126.631286388792	9.15417807048861 - 124.446547866994
wavelet-LLL_glrlm_LongRunHighGrayLevelEmphasis	wavelet-LLL	1169.28824820694 - 5821.49142475245	1224.68610387189 - 4576.61416616478
wavelet-LLL_glrlm_LongRunLowGrayLevelEmphasis	wavelet-LLL	0.0363291982047563 - 7.59436197800703	0.20541120773786 - 11.3243171903865
wavelet-LLL_glrlm_LowGrayLevelRunEmphasis	wavelet-LLL	0.00344005667501303 - 0.0190321350948987	0.00760952304516184 - 0.0245073457849296
wavelet-LLL_glrlm_RunEntropy	wavelet-LLL	6.0374885656633 - 6.60391614770537	6.06786803473127 - 6.65178459802245
wavelet-LLL_glrlm_RunLengthNonUniformity	wavelet-LLL	382662.4766516 - 2344445.60748306	671943.125565046 - 2453282.64941274
wavelet-LLL_glrlm_RunLengthNonUniformityNormalized	wavelet-LLL	0.5185534901674 - 0.694427109741976	0.515366537270564 - 0.646931824747549

wavelet-LLL_glrlm_RunPercentage	wavelet-LLL	0.305232174566913 - 0.727336678534066	0.320540928118893 - 0.610728706047325
wavelet-LLL_glrlm_RunVariance	wavelet-LLL	1.72871270134022 - 118.84852627329	6.16516232095588 - 117.004986271955
wavelet-LLL_glrlm_ShortRunEmphasis	wavelet-LLL	0.742977017103737 - 0.855421239074901	0.738005033659547 - 0.822949432688447
wavelet-LLL_glrlm_ShortRunHighGrayLevelEmphasis	wavelet-LLL	385.035904492633 - 672.274323433103	364.134337049077 - 610.50959982546
wavelet-LLL_glrlm_ShortRunLowGrayLevelEmphasis	wavelet-LLL	0.00278462885617191 - 0.0102757474321064	0.00455866324455664 - 0.0114899977720114
wavelet-LLL_glszm_GrayLevelNonUniformity	wavelet-LLL	2888.07001258989 - 17028.4028637688	5211.90400419491 - 19439.3829555243
wavelet-LLL_glszm_GrayLevelNonUniformityNormalized	wavelet-LLL	0.0233876102192276 - 0.0365631505335124	0.0220291596153737 - 0.0365342465602508
wavelet-LLL_glszm_GrayLevelVariance	wavelet-LLL	55.6689590142619 - 152.145987225261	55.5427192740002 - 159.945161315689
wavelet-LLL_glszm_HighGrayLevelZoneEmphasis	wavelet-LLL	482.901626466563 - 1123.40486199698	458.821189339584 - 907.877842400675
wavelet-LLL_glszm_LargeAreaEmphasis	wavelet-LLL	243227.065145541 - 27500534.5165386	625633.277315268 - 63719425.0631159
wavelet-LLL_glszm_LargeAreaHighGrayLevelEmphasis	wavelet-LLL	66294684.4118174 - 1173054838.56622	59707357.4994296 - 1116099354.75041
wavelet-LLL_glszm_LargeAreaLowGrayLevelEmphasis	wavelet-LLL	1054.30471292767 - 1375296.93104684	7429.75649201096 - 6904065.77457628
wavelet-LLL_glszm_LowGrayLevelZoneEmphasis	wavelet-LLL	0.00163658691351062 - 0.00610465939520761	0.00271427042659625 - 0.00569103536716218
wavelet-LLL_glszm_SizeZoneNonUniformity	wavelet-LLL	31333.9217079572 - 168961.758366866	70352.9453872595 - 289901.758801594
wavelet-LLL_glszm_SizeZoneNonUniformityNormalized	wavelet-LLL	0.264873186624148 - 0.390893522254622	0.259654670445957 - 0.410746157908271
wavelet-LLL_glszm_SmallAreaEmphasis	wavelet-LLL	0.527837006344335 - 0.649582923572	0.523038534097627 - 0.666387669394397
wavelet-LLL_glszm_SmallAreaHighGrayLevelEmphasis	wavelet-LLL	261.558922532693 - 711.618956669192	241.800423994467 - 606.581113427708
wavelet-LLL_glszm_SmallAreaLowGrayLevelEmphasis	wavelet-LLL	0.000909445559191762 - 0.00307293795021107	0.00147948991299835 - 0.00306150158616198

wavelet-LLL_glszm_ZoneEntropy	wavelet-LLL	7.63243573877517 - 8.25786422845121	7.6538106753455 - 8.25564107987491
wavelet-LLL_glszm_ZonePercentage	wavelet-LLL	0.0464109957708574 - 0.126416560378825	0.0461464122190766 - 0.127549674986485
wavelet-LLL_glszm_ZoneVariance	wavelet-LLL	243164.491413105 - 27500271.7895027	625571.810418556 - 63719143.1949151
wavelet-LLL_ngtdm_Busyness	wavelet-LLL	22.2705380347224 - 394.495497068394	69.4775021067121 - 865.341711041666
wavelet-LLL_ngtdm_Coarseness	wavelet-LLL	3.43732322765029E-06 - 3.95475948605724E-05	2.62745699428801E-06 - 0.000013691250472354
wavelet-LLL_ngtdm_Complexity	wavelet-LLL	725.71940888149 - 3173.11131960361	641.966239974465 - 2859.92075346661
wavelet-LLL_ngtdm_Contrast	wavelet-LLL	0.052834728950242 - 0.115200825319385	0.0461478848221922 - 0.117366391538274
wavelet-LLL_ngtdm_Strength	wavelet-LLL	0.00619175265676072 - 0.0755457830542407	0.00607964017292878 - 0.0365090586592004

Supplementary Table S2. PyRadiomics Feature Extraction Settings

This table summarizes the key parameter settings used in the radiomic feature extraction pipeline, including filter types, voxel resampling, normalization methods, and selected feature classes.

Parameter	Setting
Resampling Spacing	[1, 1, 1] mm
Interpolator	sitkBSpline
Voxel Array Shift	1000
Normalization	True
Normalization Scale	100
Discretization (Bin Width)	25
Enabled Image Types	Original, LoG, Wavelet
LoG σ Values	[3, 5] mm
Wavelet Basis Function	Haar (default)
Wavelet Decomposition Level	1
Enabled First-Order Features	Energy, TotalEnergy, Entropy, Minimum, 10Percentile, 90Percentile, Maximum, Mean, Median, InterquartileRange, Range, MeanAbsoluteDeviation, RobustMeanAbsoluteDeviation, RootMeanSquared, StandardDeviation, Skewness, Kurtosis, Variance, Uniformity
Enabled Shape Features	VoxelVolume, MeshVolume, SurfaceArea, SurfaceVolumeRatio, Compactness1, Compactness2, Sphericity, SphericalDisproportion, Maximum3DDiameter, Maximum2DDiameterSlice, Maximum2DDiameterColumn, Maximum2DDiameterRow, MajorAxisLength, MinorAxisLength, LeastAxisLength, Elongation, Flatness

Supplementary Table S3. Comparison of GPT-4o Diagnostic Outputs Before and After ARAMP Integration

This table presents a structured comparison between pre-training and post-training GPT-4o responses applied to brain MRI interpretation, emphasizing lesion analysis and diagnostic reasoning.

GPT Version	Response Summary	Commentary/Interpretation Improvement
Before Training (GPT-4o)	Step 1: Image Definition and Assessment<br>1-a. Define the imaging type:<br>The images are MRI scans, displayed in sequential axial slices.<br>1-b. Specify the organ being scanned:<br>The scans are of the brain.<br>1-c. Set MRI scan parameters:<br>Based on the provided images, it appears they are T1-weighted axial MRI scans (potentially with contrast), as the anatomical structures and contrast appear consistent with this type of imaging.<br>Step 2: Lesion Analysis and Differential Diagnosis<br>2-a. Characterize lesions:<br>Step 3: Treatment Strategy for Brain Metastases (Post-Imaging)<br>From the preliminary analysis:<br>No obvious evidence of lesions or metastases is observed in these slices.<br>If brain metastases were suspected clinically, additional sequences such as T2, FLAIR, or DWI, or post-contrast imaging, may help confirm subtle findings not visible here.<br>Step 4: Conclusion and Follow-Up<br>Diagnosis:<br>The current axial slices do not reveal any clear pathological findings, such as mass effect, intracranial hemorrhage, or significant lesions.<br>Recommended Treatment Plan:<br>Clinical Correlation: Verify symptoms like headaches, seizures, or neurological deficits. If these persist, further imaging or alternative modalities (e.g., PET scans or MR spectroscopy) could be useful.<br>Follow-Up Imaging:<br>If metastatic disease is suspected, contrast-enhanced studies or additional planes (e.g., sagittal or coronal) are recommended.	General image description with normal findings. Did not identify metastatic lesions despite radiologic evidence. No structured plan or stepwise lesion characterization.

	For subtle demyelinating diseases, FLAIR or DWI sequences might provide more clarity.<br>Conclusion:<br>No immediate interventions are warranted based on this imaging alone. Further evaluation depends on clinical suspicion or progression of symptoms.<br>Hypo- or hyperintense regions that suggest lesions or masses.<br>Structural abnormalities or distortions indicating edema, mass effect, or ischemia.<br>Vascular abnormalities or signs of hemorrhage.<br>2-b. Develop a differential diagnosis:<br>Potential findings may include:<br>Tumors or metastases (hyperintense areas with surrounding edema).<br>Vascular lesions (e.g., stroke or hemorrhage).<br>Infections (e.g., abscess with central necrosis).<br>Demyelinating diseases (e.g., multiple sclerosis with plaque formation).<br>Observations and Preliminary Analysis:<br>Image 1 (Base to Midbrain Axial Slices):<br>The lower brain and cerebellum are well-defined.<br>No significant midline shift or distortion of the brainstem and cerebellum is visible.<br>Basal cisterns appear open, indicating no acute mass effect.<br>No clear hyperintense lesions or abnormal contrast uptake is noted.<br>Image 2 (Midbrain to Corpus Callosum Axial Slices):<br>Ventricular system (lateral and third ventricles) appears symmetric and uncompressed.<br>Periventricular areas do not show hyperintense signals that suggest edema or demyelination.<br>Cortical structures appear intact; no focal cortical lesions are identified.<br>Image 3 (High Parietal Axial Slices):<br>Cortical sulci and gyri are normal in appearance for this resolution, with no evidence of brain atrophy.<br>No hyperintense or hypointense signals indicative of ischemic stroke, hemorrhage, or cortical lesions.	
Post-Training 1	Step 1: Image Definition and Assessment<br>1-a. Define the Imaging Type:<br>Imaging Modality: Magnetic Resonance Imaging (MRI).<br>1-b. Specify the Organ Being Scanned:<br>Focus: Brain. The axial MRI images cover the cerebrum, cerebellum, brainstem, and ventricles.	Introduced structured MRI modality description, lesion localization, multi-sequence interpretation (T1, FLAIR, DWI), and differential diagnosis. Clear improvement in

	1-c. Set MRI Scan Parameters:<br>Sequences:<br>T1-weighted: Provides structural details, useful for identifying mass effects or lesions.<br>FLAIR: Highlights subtle hyperintensities from edema or demyelination.<br>DWI (Diffusion-Weighted Imaging): Detects restricted diffusion (e.g., abscess, cellular tumors).<br>Imaging Planes: Axial views provided; complementary coronal and sagittal views may aid in surgical planning.<br>Contrast: Post-contrast imaging is recommended to assess lesion enhancement, helping differentiate between metastases, gliomas, or abscesses.<br>Step 2: Lesion Analysis and Differential Diagnosis<br>2-a. Characterize Lesions:<br>Lesion Features:<br>Multiple hyperintense regions observed on T1-weighted or FLAIR sequences, indicative of potential lesions.<br>Edema or mass effect suspected in surrounding structures.<br>No significant midline shift or ventricular compression noted.<br>Additional Features (if post-contrast imaging is available):<br>Ring-enhancing lesions or nodular patterns could indicate metastatic involvement.<br>2-b. Differential Diagnosis:<br>Brain Metastases:<br>Features: Discrete, often multiple lesions with possible surrounding edema.<br>Common in patients with known primary cancers like lung, breast, or melanoma.<br>High-Grade Gliomas:<br>Infiltrative masses with heterogeneous signal patterns and contrast enhancement.<br>Abscess (Less Likely):<br>Characterized by restricted diffusion on DWI and ring enhancement post-contrast.<br>Vascular Lesions (e.g., cavernomas):<br>May present as focal hyperintensities but lack typical metastatic or glioma patterns.<br>Step 3: Treatment Strategy for Brain Metastases (Post-Imaging)<br>Treatment Plan:<br>Surgical Resection:<br>Indicated for accessible lesions causing significant mass effect.<br>Suitable for limited metastases (1–3 lesions).<br>Radiotherapy:<br>Whole-Brain Radiotherapy (WBRT):	diagnostic accuracy and treatment recommendations.

	Used for widespread metastases.<br>Stereotactic Radiosurgery (SRS):<br>Preferred for small, localized lesions (<3 cm).<br>Systemic Therapy:<br>Targeted therapies or immunotherapy based on primary tumor histology (e.g., EGFR, HER2 mutations).<br>Supportive Care:<br>Corticosteroids to manage vasogenic edema.<br>Antiepileptic drugs for seizure control, if necessary.<br>Step 4: Conclusion and Follow-Up<br>Summary of Diagnosis:<br>Imaging findings strongly suggest brain metastases, consistent with multiple hyperintense lesions and potential edema.<br>Differential includes high-grade glioma, but metastatic disease is the leading consideration given the lesion characteristics.<br>Recommended Treatment Plan:<br>Surgical resection for symptomatic or accessible lesions.<br>WBRT or SRS for smaller or multiple lesions.<br>Systemic therapy tailored to the primary cancer profile.<br>Follow-Up Requirements:<br>Perform post-treatment MRI at 6–12 weeks to monitor therapeutic response.<br>Schedule follow-up imaging every 3–6 months for recurrence or progression.<br>Adjust management based on clinical symptoms or imaging changes.	
Post-Training 2	Step 1: Image Definition and Assessment<br>1-a. Define the Imaging Type:<br>Imaging Modality: Magnetic Resonance Imaging (MRI).<br>1-b. Specify the Organ Being Scanned:<br>Target Organ: Brain.<br>The provided images are sequential axial slices, covering the cerebrum, cerebellum, brainstem, and ventricular system.<br>1-c. Set MRI Scan Parameters:<br>Likely Imaging Sequences:<br>T1-weighted: High anatomical resolution, optimal for structural abnormalities.<br>FLAIR: Highlights subtle hyperintensities and edema, useful for lesion visualization.<br>DWI (if available): Detects areas of restricted diffusion, important for distinguishing between tumors and abscesses.	Improved clarity and specificity of lesion localization and MRI sequence interpretation. Stronger linkage to radiologic findings and differential logic. Shows advancement in edema characterization and integration of clinical context.

	Imaging Planes: Axial views included. For complete diagnostic objectives, complementary sagittal and coronal planes are recommended.<br>Contrast:<br>Pre-contrast: Evaluates baseline tissue characteristics.<br>Post-contrast: Essential for assessing lesion enhancement patterns indicative of metastases or other pathologies.<br>Step 2: Lesion Analysis and Differential Diagnosis<br>2-a. Lesion Characterization:<br>Observed Lesions:<br>Hyperintense regions seen in the provided axial slices, potentially indicative of pathologic processes.<br>No midline shift or significant ventricular compression observed in the provided images.<br>Surrounding vasogenic edema is likely based on lesion intensity gradients.<br>Enhancement patterns (if available) would further clarify lesion etiology.<br>Sequence-Based Findings:<br>T1-weighted: Regions of structural abnormality, possibly hypointense centrally if necrotic.<br>FLAIR: Hyperintense regions consistent with edema or tumor.<br>DWI (if analyzed): Restricted diffusion, if present, may suggest high cellularity (e.g., metastasis or abscess).<br>2-b. Differential Diagnosis: Based on the lesion characteristics and sequence-specific observations:<br>Brain Metastases (Most Likely):<br>Typically well-defined lesions with potential ring enhancement post-contrast.<br>Associated with surrounding edema.<br>Likely in patients with a known primary malignancy.<br>High-Grade Glioma:<br>Often exhibits infiltrative margins compared to metastatic lesions.<br>Enhancement pattern may vary; frequently less well-circumscribed.<br>Abscess:<br>Possible if diffusion restriction is observed on DWI and clinical history supports infection.<br>Vascular Lesions:<br>Less likely but may present as focal hyperintense regions (e.g., cavernoma).<br>Step 3: Treatment Strategy for Brain Metastases (Post-Imaging)	

	Proposed Treatment Plan:<br>Surgical Resection:<br>Indicated for symptomatic lesions larger than 3 cm or causing mass effect.<br>May provide diagnostic confirmation if pathology is uncertain.<br>Radiotherapy:<br>Whole-Brain Radiotherapy (WBRT): Suitable for multiple metastases.<br>Stereotactic Radiosurgery (SRS): Optimal for small, localized lesions in non-eloquent brain regions.<br>Systemic Therapy:<br>Targeted therapy or immunotherapy, guided by the molecular profile of the primary tumor.<br>Examples include EGFR inhibitors for lung cancer or HER2-targeted agents for breast cancer.<br>Supportive Care:<br>Corticosteroids: Reduce perilesional edema and improve neurological symptoms.<br>Antiepileptic Drugs: Administered if seizures are reported.<br>Step 4: Conclusion and Follow-Up<br>Diagnosis:<br>Imaging findings are most consistent with brain metastases, pending further clinical correlation and histopathological confirmation.<br>Recommended Treatment Plan:<br>Multimodal approach including resection (if feasible), radiotherapy, and systemic therapy tailored to the patient's primary malignancy and overall condition.<br>Follow-Up Requirements:<br>Imaging:<br>Post-treatment MRI in 6–12 weeks to evaluate therapeutic response.<br>Routine imaging every 3–6 months to monitor for recurrence or progression.<br>Clinical Monitoring:<br>Neurological symptom assessment.<br>Tumor marker evaluation based on primary cancer.	

Post-Training 3	Step 1: Image Definition and Assessment<br>1-a. Define the Imaging Type:<br>Imaging Modality: Magnetic Resonance Imaging (MRI).<br>1-b. Specify the Organ Being Scanned:<br>Target Organ: Brain.<br>Provided axial slices cover the cerebral hemispheres, cerebellum, brainstem, and ventricular system.<br>1-c. Set MRI Scan Parameters:<br>Likely Imaging Sequences:<br>T1-weighted: High-resolution structural imaging.<br>FLAIR: Enhanced visualization of edema and subtle hyperintensities.<br>DWI: Detects restricted diffusion indicative of cellular lesions or ischemia.<br>Imaging Planes: Axial slices provided; complementary sagittal and coronal planes are recommended for complete analysis.<br>Contrast:<br>Pre-contrast: Baseline tissue differentiation.<br>Post-contrast: Identifies enhancement patterns critical for characterizing tumors or metastases.<br>Step 2: Lesion Analysis and Differential Diagnosis<br>2-a. Lesion Characterization:<br>Observations:<br>Hyperintense regions in the axial slices, with apparent structural distortion suggestive of pathological lesions.<br>Surrounding areas likely indicate vasogenic edema.<br>No gross midline shift or ventricular obstruction observed.<br>Sequence-Based Findings:<br>T1-weighted: Likely demonstrates hypo- to isointense lesions; post-contrast could show ring enhancement.<br>FLAIR: Hyperintense lesions with distinct boundaries, possibly indicating edema or active tumor regions.<br>DWI: Expected restricted diffusion if the lesions are highly cellular (e.g., metastases).<br>2-b. Differential Diagnosis:<br>Brain Metastases (Most Likely):<br>Common in patients with a history of systemic malignancy.<br>Features: Well-defined lesions, potential ring enhancement on post-contrast, perilesional edema.	Most comprehensive interpretation with refined language, consistent terminology, and actionable multimodal recommendations. Provides optimal radiologic insight and clinical reasoning.

	High-Grade Glioma:<br>May exhibit infiltrative margins with irregular enhancement patterns.<br>Frequently associated with localized edema.<br>Abscess:<br>Unlikely but possible if diffusion restriction and clinical signs of infection are present.<br>Vascular Lesions:<br>May mimic neoplasms but generally have distinct imaging features like hemosiderin deposition.<br>Step 3: Treatment Strategy for Brain Metastases (Post-Imaging)<br>Proposed Treatment Plan:<br>Surgical Resection:<br>Indicated for symptomatic lesions (>3 cm) or significant mass effect.<br>Allows for histopathological confirmation and symptomatic relief.<br>Radiotherapy:<br>Whole-Brain Radiotherapy (WBRT):<br>Suitable for multiple lesions or extensive metastases.<br>Stereotactic Radiosurgery (SRS):<br>Recommended for small, localized lesions, particularly in non-eloquent brain regions.<br>Systemic Therapy:<br>Targeted therapies or immunotherapies based on the molecular profile of the primary malignancy.<br>Examples:<br>EGFR inhibitors for lung adenocarcinoma.<br>HER2-targeted agents for breast cancer.<br>Supportive Care:<br>Corticosteroids: To reduce edema and alleviate symptoms.<br>Antiepileptic medications: If seizures are reported.<br>Step 4: Conclusion and Follow-Up<br>Diagnosis:<br>Imaging findings strongly suggest brain metastases, pending further clinical correlation and histological confirmation.<br>Recommended Treatment Plan:<br>A multimodal approach is advised, including resection for symptomatic relief, radiotherapy for disease control, and systemic therapy tailored to the primary malignancy.<br>Follow-Up Requirements:	

	Imaging:<br>Post-treatment MRI at 6–12 weeks to evaluate response.<br>Routine surveillance imaging every 3–6 months to monitor for progression or recurrence.<br>Clinical Monitoring:<br>Regular neurological exams.<br>Tumor marker evaluation and systemic imaging for primary cancer assessment.	

Supplementary Table S4. Comparative Overview of ARAMP and Traditional AI Models

Comparison Aspect	ARAMP (This Study)	Traditional Deep Learning Models (e.g., 3D ResNet, CNN, Transformer)
Functional Role	Interpretive support, structured reasoning	Image-level classification or segmentation
Input Type	Extracted radiomic features + image context + clinical descriptors	Raw DICOM image volumes
Training Requirement	None (zero-shot via GUI prompting)	Requires labeled datasets and training/fine-tuning
Architecture	RAG-based prompting with LLM (GPT-4o)	CNNs / Transformers with trainable layers
Multimodal Reasoning	Yes (text + imaging metadata + reference integration)	Limited or absent (unless explicitly designed)
Real-time Citation & Reference	Yes (retrieves and cites source literature)	No (unless manually programmed or post-processed)
Knowledge Update Mechanism	Version-controlled, prompt-updated external knowledge base	Static weights post-training
Explainability	High (narrative explanation, source-backed)	Low to moderate (via Grad-CAM, SHAP, etc.)
Inference Mode	Zero-shot prompting via GUI (ChatGPT Pro)	Batch inference via scripts or APIs

Note:

This comparison table was constructed by the authors based on key differences between the ARAMP framework and representative traditional deep learning models used in brain metastasis detection, including 3D CNNs and Transformer-based architectures (Grøvik et al., 2019; Liu et al., 2022; Hatamizadeh et al., 2022; Xing et al., 2022).

References:

1. Grøvik, E., Yi, D., Iv, M., Tong, E., Rubin, D. L., & Zaharchuk, G. (2019). Deep learning enables automatic detection and segmentation of brain metastases on multi-sequence MRI. arXiv preprint arXiv:1903.07988.
2. Liu, Y., Mu, F., Shi, Y., Cheng, J., Li, C., & Chen, X. (2022). Brain tumor segmentation in multimodal MRI via pixel-level and feature-level image fusion. Frontiers in Neuroscience, 16, 1000587.
3. Hatamizadeh, A., Nath, V., Tang, Y., Yang, D., Roth, H., & Xu, D. (2022). Swin UNETR: Swin Transformers for semantic segmentation of brain tumors in MRI images. arXiv preprint arXiv:2201.01266.

4. Xing, Z., Yu, L., Wan, L., Han, T., & Zhu, L. (2022). NestedFormer: Nested modality-aware transformer for brain tumor segmentation. arXiv preprint arXiv:2208.14876.

Reference Cross-Check Table: Supplementary Table 5 vs. Cited Literature

Comparison Aspect	Traditional Model Feature	Matching Literature	Reference Suggestion
Functional Role	Image-level classification or segmentation	Voxel-wise segmentation tasks	Grøvik et al. 2019, Hatamizadeh et al. 2022
Input Type	Raw DICOM image volumes	Used as input in deep CNN/Transformer segmentation	All listed
Training Requirement	Requires labeled datasets and training	Supervised training required in all	All listed
Architecture	CNNs / Transformers	Swin UNETR (Transformer); NestedFormer	Hatamizadeh et al. 2022, Xing et al. 2022
Multimodal Reasoning	Limited or absent	Pixel-level fusion, modality-aware input	Liu et al. 2022, Xing et al. 2022
Explainability	Low to moderate (Grad-CAM, SHAP)	Generally requires post-hoc explanation	Selvaraju et al. 2017 (Grad-CAM)*

Supplementary Table S5. Expert Radiologist Scoring for Pre- vs. Post-Training Outputs and Inter-Rater Agreement

Description:

This table summarizes the individual and average scores assigned by five board-certified radiologists to AI-generated MRI interpretations before and after the ARAMP training process. Each row corresponds to one case instance. Ratings were provided on a 5-point scale (converted to 0–100%) across clinical relevance, accuracy, and interpretive clarity dimensions. The table includes mean scores across raters, intra-inference agreement levels, and calculated changes following training. These ratings were used in the inferential statistics reported in Table 3 of the main manuscript.

6-1.5-point scale

ImageNo/User	Pre_Dr.A	Post_Dr.A	Inter_Dr.A	Pre_Dr.B	Post_Dr.B	Inter_Dr.B	Post_Dr.C	Inter_Dr.C	Pre_Gemini	Post_Gemini
MRI1	-	4	2	1	5	2	5	2	5	5
MRI2	-	3	1	2	4	2	4	2	3	2
MRI3	-	4	1	3	3	2	3	1	3	2
MRI4	-	2	1	3	4	2	5	2	3	2
MRI5	-	3	2	3	3	3	4	2	4	4
MRI6	-	2	2	4	4	2	4	2	3	2
MRI7	-	2	1	3	3	2	4	1	3	4
MRI8	-	3	2	3	4	3	4	2	4	5
MRI9	-	3	2	2	4	2	3	1	3	4
MRI10	-	5	1	1	5	1	5	1	5	5
MRI11	-	3	1	3	3	2	3	2	3	4
MRI12	-	5	1	1	5	2	5	1	5	5
MRI13	-	2	1	2	4	2	4	2	3	2
MRI14	-	3	1	2	4	2	4	3	3	2
MRI15	-	4	1	2	5	3	4	2	4	4
MRI16	-	2	1	3	3	2	5	2	3	2

MRI17	-	3	1	1	5	2	4	1	3	4
MRI18	-	5	1	2	5	1	3	1	3	2
MRI19	-	2	1	3	3	2	4	2	3	2
MRI20	-	2	2	2	4	2	4	2	3	4
MRI21	-	4	1	1	4	2	3	3	4	4
MRI22	-	4	1	1	4	3	5	2	5	5
MRI23	-	4	1	1	4	2	5	2	5	5
MRI24	-	1	2	3	3	2	5	2	3	2
MRI25	-	4	1	4	2	2	5	2	4	4
MRI26	-	4	2	4	4	3	3	2	3	4
MRI27	-	2	1	2	4	2	3	1	3	4
MRI28	-	2	1	3	3	1	5	2	4	5
MRI29	-	1	1	4	4	3	2	2	2	4
MRI30	-	4	1	4	2	2	5	2	5	5
MRI31	-	2	1	4	4	2	5	2	3	4
MRI32	-	1	1	3	3	1	4	1	3	3
MRI33	-	1	1	4	4	2	4	1	3	2
MRI34	-	1	1	3	4	2	3	1	3	4
MRI35	-	3	3	3	3	2	3	1	3	2
MRI36	-	3	1	3	4	1	4	1	3	4
MRI37	-	1	1	4	4	1	3	2	2	4
MRI38	-	1	1	3	4	2	3	2	2	4
MRI39	-	1	1	3	3	2	4	2	3	4
MRI40	-	4	1	2	4	2	4	3	3	4
MRI41	-	4	1	3	4	3	4	2	3	4
MRI42	-	1	1	4	4	2	3	2	3	2
MRI43	-	4	1	3	3	2	4	3	3	4
MRI44	-	4	1	3	4	3	4	2	2	3
MRI45	-	4	1	2	4	2	4	2	5	5

MRI46	-	3	3	2	4	2	4	2	3	2
MRI47	-	2	1	3	4	2	4	2	3	2
MRI48	-	4	1	1	4	2	5	2	5	5
MRI49	-	4	1	3	3	3	4	2	3	2
MRI50	-	2	1	2	4	2	4	1	3	2
MRI51	-	1	1	3	4	1	3	1	3	2
MRI52	-	1	1	3	3	1	4	2	3	2
MRI53	-	4	1	2	4	2	5	3	5	5
MRI54	-	4	1	4	2	3	4	1	4	5
MRI55	-	1	1	4	4	1	4	2	3	2
MRI56	-	3	1	2	4	3	5	3	3	2
MRI57	-	2	1	3	3	3	4	2	3	4
MRI58	-	3	1	3	4	3	4	2	3	4
MRI59	-	3	1	3	4	3	4	2	3	4
MRI60	-	2	1	3	4	2	4	2	3	4
MRI61	-	2	1	2	4	2	4	2	3	2
MRI62	-	4	1	2	4	2	4	2	3	2
MRI63	-	5	1	3	4	2	5	2	5	5
MRI64	-	1	1	3	4	2	4	2	3	2
MRI65	-	1	1	3	3	2	5	3	3	4
MRI66	-	4	1	4	2	3	3	2	4	4
MRI67	-	3	1	3	3	2	4	2	2	4
MRI68	-	4	1	3	4	2	3	2	3	3
MRI69	-	2	1	3	4	4	4	3	2	2
MRI70	-	2	1	3	4	2	4	2	4	2
MRI71	-	5	1	3	4	2	3	2	3	4
MRI72	-	5	1	3	3	3	3	2	3	4
MRI73	-	4	1	4	2	3	2	2	4	4
MRI74	-	4	1	3	3	2	4	2	4	4

MRI75	-	2	1	3	4	2	4	2	3	3
MRI76	-	4	1	2	4	2	5	2	5	5
MRI77	-	3	1	3	4	2	3	2	3	4
MRI78	-	1	1	3	3	3	3	2	3	4
MRI79	-	3	1	3	3	2	4	1	3	4
MRI80	-	2	1	3	4	3	2	1	3	3
MRI81	-	1	1	1	1	1	1	1	3	4
MRI82	-	1	1	1	1	1	1	1	3	2
MRI83	-	2	1	3	4	1	4	1	3	2
MRI84	-	3	1	3	5	1	2	1	3	2
MRI85	-	1	1	1	1	1	1	1	3	2
MRI86	-	2	1	3	4	2	4	1	3	2
MRI87	-	2	1	3	5	1	4	2	3	4
MRI88	-	4	3	3	4	2	4	2	4	2
MRI89	-	2	1	3	4	2	4	2	3	2
MRI90	-	1	1	1	1	1	1	1	3	4
MRI91	-	2	1	3	4	2	4	2	3	2
MRI92	-	2	1	3	4	2	3	2	1	3
MRI93	-	2	1	3	4	2	4	1	3	3
MRI94	-	5	1	3	4	2	4	2	5	5
MRI95	-	5	1	2	5	1	5	1	5	5
MRI96	-	3	1	2	4	2	4	2	3	4
MRI97	-	3	1	2	4	1	4	1	3	4
MRI98	-	4	1	2	5	1	3	1	3	4
MRI99	-	4	1	3	3	3	4	1	3	2
MRI100	-	5	1	2	4	1	4	1	5	5

6-2 .converted to 0–100%

ImageNo/User	**Pre_Dr.A**	**Pre_Dr.A**	**Pre_Dr.A**	**Pre_Dr.B**	**Post_Dr.B**	**Inter_Dr.B**	**Post_Dr.C**	**Inter_Dr.C**	**Pre_Gemini**	**Post_Gemini**	**Inter_Gemini**
MRI1	-	80	40	20	100	40	100	40	100	100	20
MRI2	-	60	20	40	80	40	80	40	60	40	20
MRI3	-	80	20	60	60	40	60	20	60	40	20
MRI4	-	40	20	60	80	40	100	40	60	40	40
MRI5	-	60	40	60	60	60	80	40	80	80	40
MRI6	-	40	40	80	80	40	80	40	60	40	20
MRI7	-	40	20	60	60	40	80	20	60	80	40
MRI8	-	60	40	60	80	60	80	40	80	100	20
MRI9	-	60	40	40	80	40	60	20	60	80	20
MRI10	-	100	20	20	100	20	100	20	100	100	20
MRI11	-	60	20	60	60	40	60	40	60	80	40
MRI12	-	100	20	20	100	40	100	20	100	100	20
MRI13	-	40	20	40	80	40	80	40	60	40	20
MRI14	-	60	20	40	80	40	80	60	60	40	20
MRI15	-	80	20	40	100	60	80	40	80	80	20
MRI16	-	40	20	60	60	40	100	40	60	40	40
MRI17	-	60	20	20	100	40	80	20	60	80	20
MRI18	-	100	20	40	100	20	60	20	60	40	20
MRI19	-	40	20	60	60	40	80	40	60	40	20
MRI20	-	40	40	40	80	40	80	40	60	80	40
MRI21	-	80	20	20	80	40	60	60	80	80	20
MRI22	-	80	20	20	80	60	100	40	100	100	20
MRI23	-	80	20	20	80	40	100	40	100	100	20
MRI24	-	20	40	60	60	40	100	40	60	40	20
MRI25	-	80	20	80	40	40	100	40	80	80	20
MRI26	-	80	40	80	80	60	60	40	60	80	20
MRI27	-	40	20	40	80	40	60	20	60	80	20
MRI28	-	40	20	60	60	20	100	40	80	100	20
MRI29	-	20	20	80	80	60	40	40	40	80	20
MRI30	-	80	20	80	40	40	100	40	100	100	20
MRI31	-	40	20	80	80	40	100	40	60	80	20

MRI32	-	20	20	60	60	20	80	20	60	60	20
MRI33	-	20	20	80	80	40	80	20	60	40	20
MRI34	-	20	20	60	80	40	60	20	60	80	40
MRI35	-	60	60	60	60	40	60	20	60	40	40
MRI36	-	60	20	60	80	20	80	20	60	80	20
MRI37	-	20	20	80	80	20	60	40	40	80	20
MRI38	-	20	20	60	80	40	60	40	40	80	20
MRI39	-	20	20	60	60	40	80	40	60	80	40
MRI40	-	80	20	40	80	40	80	60	60	80	20
MRI41	-	80	20	60	80	60	80	40	60	80	20
MRI42	-	20	20	80	80	40	60	40	60	40	20
MRI43	-	80	20	60	60	40	80	60	60	80	20
MRI44	-	80	20	60	80	60	80	40	40	60	20
MRI45	-	80	20	40	80	40	80	40	100	100	20
MRI46	-	60	60	40	80	40	80	40	60	40	20
MRI47	-	40	20	60	80	40	80	40	60	40	20
MRI48	-	80	20	20	80	40	100	40	100	100	20
MRI49	-	80	20	60	60	60	80	40	60	40	20
MRI50	-	40	20	40	80	40	80	20	60	40	20
MRI51	-	20	20	60	80	20	60	20	60	40	20
MRI52	-	20	20	60	60	20	80	40	60	40	20
MRI53	-	80	20	40	80	40	100	60	100	100	20
MRI54	-	80	20	80	40	60	80	20	80	100	20
MRI55	-	20	20	80	80	20	80	40	60	40	20
MRI56	-	60	20	40	80	60	100	60	60	40	20
MRI57	-	40	20	60	60	60	80	40	60	80	20
MRI58	-	60	20	60	80	60	80	40	60	80	20
MRI59	-	60	20	60	80	60	80	40	60	80	20
MRI60	-	40	20	60	80	40	80	40	60	80	20
MRI61	-	40	20	40	80	40	80	40	60	40	20
MRI62	-	80	20	40	80	40	80	40	60	40	20
MRI63	-	100	20	60	80	40	100	40	100	100	20

MRI64	-	20	20	60	80	40	80	40	60	40	20
MRI65	-	20	20	60	60	40	100	60	60	80	20
MRI66	-	80	20	80	40	60	60	40	80	80	20
MRI67	-	60	20	60	60	40	80	40	40	80	20
MRI68	-	80	20	60	80	40	60	40	60	60	20
MRI69	-	40	20	60	80	80	80	60	40	40	20
MRI70	-	40	20	60	80	40	80	40	80	40	20
MRI71	-	100	20	60	80	40	60	40	60	80	20
MRI72	-	100	20	60	60	60	60	40	60	80	20
MRI73	-	80	20	80	40	60	40	40	80	80	20
MRI74	-	80	20	60	60	40	80	40	80	80	20
MRI75	-	40	20	60	80	40	80	40	60	60	20
MRI76	-	80	20	40	80	40	100	40	100	100	20
MRI77	-	60	20	60	80	40	60	40	60	80	20
MRI78	-	20	20	60	60	60	60	40	60	80	20
MRI79	-	60	20	60	60	40	80	20	60	80	20
MRI80	-	40	20	60	80	60	40	20	60	60	40
MRI81	-	20	20	20	20	20	20	20	60	80	20
MRI82	-	20	20	20	20	20	20	20	60	40	20
MRI83	-	40	20	60	80	20	80	20	60	40	20
MRI84	-	60	20	60	100	20	40	20	60	40	20
MRI85	-	20	20	20	20	20	20	20	60	40	20
MRI86	-	40	20	60	80	40	80	20	60	40	20
MRI87	-	40	20	60	100	20	80	40	60	80	20
MRI88	-	80	60	60	80	40	80	40	80	40	20
MRI89	-	40	20	60	80	40	80	40	60	40	20
MRI90	-	20	20	20	20	20	20	20	60	80	20
MRI91	-	40	20	60	80	40	80	40	60	40	20
MRI92	-	40	20	60	80	40	60	40	20	60	20
MRI93	-	40	20	60	80	40	80	20	60	60	20
MRI94	-	100	20	60	80	40	80	40	100	100	20
MRI95	-	100	20	40	100	20	100	20	100	100	20

MRI96	-	60	20	40	80	40	80	40	60	80	20
MRI97	-	60	20	40	80	20	80	20	60	80	20
MRI98	-	80	20	40	100	20	60	20	60	80	20
MRI99	-	80	20	60	60	60	80	20	60	40	20
MRI100	-	100	20	40	80	20	80	20	100	100	20

Supplementary Table S6. CLAIM 2024 Checklist with Study Mapping

Section/Topic	Item No.	Checklist Item	This Study Mapping
TITLE/ABSTRACT	1	Identification as a study of AI methodology	The title identifies the methodology as an AI-integrated interpretive framework (ARAMP) using GPT-4o.
TITLE/ABSTRACT	2	Summary of study design, methods, results, and conclusions	Abstract outlines a retrospective study with GPT-4o zero-shot inference and radiomic validation.
INTRODUCTION	3	Scientific and/or clinical background, including the intended use and role of the AI approach	Describes unmet needs in diagnostic interpretation of brain metastases and ARAMP's role in structured reporting.
INTRODUCTION	4	Study aims, objectives, and hypotheses	The goal is to evaluate whether ARAMP improves interpretive performance through structured reasoning.
METHODS - Study Design	5	Prospective or retrospective study	This is a retrospective, single-center study.
METHODS - Study Design	6	Study goal	Evaluate interpretive enhancement of GPT-4o responses after integrating structured radiomic features and retrieval-augmented generation (RAG) with literature-informed prompts.
METHODS - Data	7	Data sources	Imaging data from institutional PACS (2010–2022), specifically contrast-enhanced axial T1-weighted brain MRI (T1+C), linked with cancer registry.
METHODS - Data	8	Inclusion and exclusion criteria	Inclusion: Age ≥20 years, received contrast-enhanced axial T1-weighted brain MRI (T1+C); cancer group required imaging-confirmed brain metastasis; control group had no cancer diagnosis within −60 days to +1 year of scan. Exclusion: incomplete or poor-quality MRI.<br>This item is further supported by detailed sampling procedures and exclusion criteria described in Supplementary Table 1 and Figure 1. Stratification by cancer type ensured epidemiological relevance to East Asian populations.
METHODS - Data	9	Data preprocessing	Images were normalized to [0,1] and features extracted with PyRadiomics. Radiomic features were extracted and

			organized into a structured dataset (No_abnormalities & Brain_Meta.xlsx). The dataset included a "TYPE" column specifying feature categories (e.g., original_shape_VoxelVolume), and separate columns for "No_abnormalities" and "Brain_Meta" groups. This structure enabled efficient cross-group comparison and validation.
METHODS - Data	10	Selection of data subsets	Stratified sampling by cancer type and matched control group by age and gender. The structured dataset facilitated transparent stratification by feature type and group. Feature subsets could be directly accessed for analysis based on the 'TYPE' and group-specific columns.<br>This stratified sampling approach minimized demographic bias and enhanced cohort representativeness, in line with CLAIM standards.
METHODS - Data	11	De-identification methods	All DICOMs were anonymized, filenames stripped of identifiers.
METHODS - Data	12	How missing data were handled	No missing data were present in the final dataset.
METHODS - Data	13	Image acquisition protocol	All brain MRI images were acquired as axial T1-weighted post-contrast (T1+C) sequences with gadolinium enhancement, using standardized parameters across all patients.<br>See Supplementary Table 3 for image enhancement parameters (resampling, normalization, voxel shift) and preprocessing consistency.
METHODS - Reference Standard	14	Definition of reference standard	Consensus from board-certified radiologists based on original clinical reports and visual review.
METHODS - Reference Standard	15	Rationale for choosing the reference standard	Reflects real-world radiological practice; avoids annotation ambiguity.
METHODS - Reference Standard	16	Source of reference standard annotations	Based on original radiology reports and expert confirmation.<br>Labeling was based on radiologist consensus and confirmed MRI reports. Meta and No_abnormalities groups were structured accordingly.
METHODS - Reference Standard	17	Annotation of test set	Not applicable; no segmentation or pixel-level labeling performed.

METHODS - Reference Standard	18	Inter-/intra-rater variability	Three board-certified radiologists independently rated all outputs. Inter-rater agreement was assessed using Intraclass Correlation Coefficient (ICC), with results reported in Table 2.<br>The full workflow for image conversion, layout standardization, and interpretive preparation is visualized in Supplementary Figure 2.
METHODS - Data Partitions	19	How data were assigned to partitions	The cancer and control groups each included 100 patients, totaling 200 imaging cases**.** Only the 100 cancer **cases** underwent two diagnostic workflows: **(1)** radiomics-based prompting with RAG integration**, and (2)** radiomics-based prompting without RAG support**,** enabling within-subject comparison.
METHODS - Data Partitions	20	Level at which partitions are disjoint	Not disjoint; the same patients were evaluated under two conditions (with and without RAG) to enable within-subject comparison.
METHODS - Testing Data	21	Intended sample size	A total of 200 cases were selected, consisting of 100 patients with confirmed brain metastases (cancer group) and 100 matched individuals without any known malignancy (control group). From a total of 2,447 eligible cancer patients who received AX T1+C brain MRI between 2010–2022, 100 cancer cases were sampled using stratified random sampling based on cancer type. The control group was matched one-to-one by age and sex to mirror the demographic profile of the cancer group, and all control subjects had no cancer diagnosis recorded from 60 days prior to 1 year after their MRI.<br>Demographic metadata for all subjects—including age, sex, and cancer type (for the cancer group)—were independently cross-verified using the institutional registry. A complete breakdown is provided in Table 1.
METHODS - Model	22	Detailed model description	The system utilized GPT-4o (May 2024 version) via GUI-based zero-shot prompting. Input prompts were structured using patient-specific radiomic features (PyRadiomics) and optionally combined with dynamic literature retrieval (RAG). Outputs included stepwise diagnostic reasoning, citation of relevant studies, and

			natural language explanation. No model training or fine-tuning was performed. The sampled cancer types reflect national epidemiological trends in East Asia, ensuring external relevance.
METHODS - Model	23	Software libraries, frameworks, and packages	The study used ChatGPT (GPT-4o, May 2024) via OpenAI GUI for zero-shot inference. Radiomic features were extracted using PyRadiomics within a dedicated Anaconda environment (Python 3.7.16). Data preprocessing and result formatting were performed using Pandas and NumPy.
METHODS - Model	24	Initialization of model parameters	Not applicable. The model (GPT-4o, May 2024) is pre-initialized by OpenAI and used in inference-only mode via GUI. No parameters were configured or exposed for initialization or fine-tuning.<br>Image format, PNG structure, metadata linkage, and folder hierarchy follow CLAIM compatibility and interpretability guidelines (see Supplementary Figure 2). The diagnostic workflow followed a standardized 4-step reasoning process using GPT-4o. Composite images (up to 10 MR slices per case) were analyzed to define scan type, assess lesions, determine treatment strategies, and recommend follow-up, with dynamic reference integration at each step (see Section 2.5.3).
METHODS - Training	25	Details of training approach	Not applicable. No model training, optimization, or fine-tuning was performed. GPT-4o was used as a proprietary pre-trained model via GUI interface. The study involved zero-shot inference only, without access to model weights or training pipeline.
METHODS - Training	26	Method of selecting the final model	Not applicable. The system used a single pre-trained GPT-4o model with no model selection or tuning performed.
METHODS - Training	27	Ensembling techniques	None used. The study involved single-pass inference using one GPT-4o model per case.
METHODS - Evaluation	28	Metrics of model performance	Sensitivity, plausibility, consistency, helpfulness.
METHODS - Evaluation	29	Statistical significance and uncertainty	Model performance was assessed using four qualitative criteria: sensitivity (from TP/FN counts), plausibility, consistency, and helpfulness. The latter three were rated

			by three physicians on a 5-point Likert scale; scores were converted to 0–100% scale for analysis.
METHODS - Evaluation	30	Robustness or sensitivity analysis	Robustness was evaluated by applying the same cases across three post-training rounds with refined prompts and structured inputs. Consistency and performance improvement were assessed longitudinally.
METHODS - Evaluation	31	Explainability or interpretability	Model outputs included stepwise diagnostic reasoning, citation of relevant studies (via RAG), and natural language explanation, allowing physician review and plausibility scoring.<br>Explainability was supported by structured prompting across four diagnostic stages: image assessment, lesion characterization, treatment planning, and follow-up. Each stage incorporated real-time literature retrieval and visual cues from composite MR images.
METHODS - Evaluation	32	Evaluation on internal data	Yes, entire dataset is internal.
METHODS - Evaluation	33	Testing on external data	No. The study was limited to retrospective internal data, and generalizability requires further external validation.
METHODS - Evaluation	34	Clinical trial registration	Not applicable. This study was retrospective, observational, and did not involve any patient intervention, thus not requiring clinical trial registration. This study was approved by the Institutional Review Board of China Medical University Hospital (IRB No. CMUH111-REC1-206(CR-2)). The requirement for informed consent was waived due to the retrospective design and use of de-identified data.
RESULTS - Data	35	Inclusion and exclusion count	From 2,447 eligible cancer patients, 100 were selected using stratified random sampling by cancer type. An additional 100 matched non-cancer controls were selected using the same imaging protocol. Inclusion and exclusion criteria and patient flow are detailed in Figure 1.<br>Feature distributions across cancer and control groups were stored in the No_abnormalities & Brain_Meta.xlsx file. The labeled format enabled direct comparison and inclusion count tracking.

RESULTS - Data	36	Demographic and clinical characteristics	Demographic data including gender, age (mean ± SD), and primary cancer type distribution were reported. Detailed breakdown is presented in Table 1. Expert ratings were converted to a 0–100% scale. Full scoring matrices (radiologists vs. Gemini) are detailed in Supplementary Table 6.
RESULTS - Model Performance	37	Model performance and uncertainty	Model sensitivity improved from 0.85 (pre-training) to 0.98 (after RAG integration), with standard deviation and 95% confidence intervals reported in Table 4.<br>Supplementary Table 6 confirms post-training gains in interpretive clarity and inter-rater agreement, with statistical significance ($p = 0.01$).
RESULTS - Model Performance	38	Diagnostic performance estimates	Specificity and overall accuracy were not reported due to lack of expert-verified negative metastasis labels in the control group, which may otherwise introduce mislabeling bias. The evaluation focused on interpretive quality rather than binary classification.
RESULTS - Model Performance	39	Error analysis	Representative examples of incorrect or ambiguous GPT responses were analyzed and discussed in the text. These include hallucinated findings, inconsistent logic, and vague conclusions. Selected cases are shown in Supplementary Table 3.
DISCUSSION	40	Study limitations	Limitations include single-center retrospective design, lack of segmentation labels, and absence of external validation. These factors may limit the generalizability of findings to broader clinical settings.
DISCUSSION	41	Clinical implications	The AI system is intended to assist radiologists in structured interpretation and differential reasoning for ambiguous brain MRI findings. It is not designed for screening or primary diagnosis.<br>The system provides a stepwise interpretive framework for ambiguous brain lesions, supporting clinical decision-making for brain metastases through structured GPT-4o outputs aligned with standard diagnostic and treatment pathways.
OTHER INFORMATION	42	Availability of full protocol or	The full YAML configuration file (Supplementary File 1) and radiomic

		technical appendix	feature extraction parameters (Supplementary Table 2) are provided to enable workflow reproducibility.
OTHER INFORMATION	43	Availability of code, models, and data	De-identified MRI data, radiomic feature tables, and GPT prompt templates are available upon reasonable request to the corresponding author. Pre-trained GPT-4o model is proprietary and not included. Dataset versioning and standardized image structures support full traceability. Folder layout and metadata are described in Supplementary Figure 2.
OTHER INFORMATION	44	Funding source and role of funders	This study was supported by an internal research grant from China Medical University Hospital (CMUH). The funder had no role in study design, data collection, model development, analysis, interpretation, or manuscript writing.

Note: ARAMP Execution Workflow Summary

The ARAMP (Adaptive RAG Assistant MRI Platform) system was executed using a multi-stage, prompt-driven framework built around GPT-4o. Key components of the workflow included:

1. Image Preprocessing & Feature Extraction: DICOM brain MRIs (AX_T1+C) were normalized and converted to PNG composites (up to 10 slices per case). A total of 1,051 radiomic features were extracted using PyRadiomics, spanning first-order statistics, shape descriptors, LoG, and wavelet features.
2. Prompt Construction with Structured Inputs: Each patient case was converted into a multimodal input, combining radiomic feature vectors with composite images. Prompts were designed to guide GPT-4o through four diagnostic stages: image type identification, lesion analysis, treatment reasoning, and follow-up planning.
3. Retrieval-Augmented Generation (RAG) Integration: During the diagnostic process, the system dynamically retrieved relevant literature passages based on extracted feature cues, supporting GPT inference with evidence-based reasoning.
4. Zero-Shot Inference across Multiple Rounds: For each case, the model generated one baseline report (pre-training) and three enhanced reports (post-training), incorporating refined prompts and RAG integration—without any fine-tuning or parameter updates. This simulated a form of prompt-based "training" behavior.
5. Expert Evaluation & Statistical Analysis: Outputs were scored by three radiologists across plausibility, helpfulness, and consistency. Post-intervention sensitivity rose from 0.84 to 0.98 (Table 4), with statistically significant inter-rater agreement confirmed via ICC analysis (Table 2 & Supplementary Table 6).
